# Integration of crop modeling and sensing into molecular breeding for nutritional quality and stress tolerance

**DOI:** 10.1007/s00122-025-04984-y

**Published:** 2025-08-08

**Authors:** Jonathan Berlingeri, Abelina Fuentes, Earl Ranario, Heesup Yun, Ellen Y. Rim, Oscar Garrett, Alexander Howard, Mary-Francis LaPorte, Sassoum Lo, Duke Pauli, Jenna Hershberger, Mason Earles, Allen Van Deynze, Edward Charles Brummer, Richard Michelmore, Christopher Y. S. Wong, Troy S. Magney, Pamela C. Ronald, Daniel E. Runcie, Brian N. Bailey, Christine H. Diepenbrock

**Affiliations:** 1 Department of Plant Sciences, University of California, Davis, USA; 2 Department of Biological & Agricultural Engineering, University of California, Davis, USA; 3 Department of Plant Pathology, University of California, Davis, USA; 4 School of Plant Sciences, University of Arizona, Tucson, USA; 5 Department of Plant and Environmental Sciences, Pee Dee Research and Education Center, Clemson University, Florence, SC USA; 6 Genome Center, University of California, Davis, USA; 7 Faculty of Forestry and Environmental Management, University of New Brunswick, Fredericton, NB Canada

## Abstract

Integrating innovative technologies into plant breeding is critical to bolster food and nutritional security under biotic and abiotic stresses in changing climates. While breeding efforts have focused primarily on yield and stress tolerance, emerging evidence highlights the need to also prioritize nutritional quality. Advanced molecular breeding approaches have enhanced our ability to develop improved crop varieties and could be substantially informed by the routine integration of crop modeling and remote sensing technologies. This review article discusses the potential of combining crop modeling and sensing with molecular breeding to address the dual challenge of nutritional quality and stress tolerance. We provide overviews of stress response strategies, challenges in breeding for quality traits, and the use of environmental data in genomic prediction. We also describe the status of crop modeling and sensing technologies in grain legumes, rice, and leafy greens, alongside the status of -omics tools in these crops and the use of AI with directed evolution to identify novel resistance genes. We describe the pairwise and three-way integration of AI-enabled sensing and biophysically and empirically constrained crop modeling into breeding to enable prediction of phenotypic and breeding values and dissection of genotype-by-environment-by-management interactions with increasing fidelity, efficiency, and temporal/spatial resolution to inform selection decisions. This article highlights current initiatives and future trends that focus on leveraging these advancements to develop more climate-resilient and nutritionally dense crops, ultimately enhancing the effectiveness of molecular breeding.

## Introduction

Increased crop yields and stress tolerance have been consistent primary targets in plant breeding due to their importance for food, nutritional, and economic security. Biotic (living or viral) stresses and abiotic (non-living) stresses continue to constrain our collective efforts to achieve an adequate and nutritious food supply (Newton et al. 2011; Myers et al. 2017; Fanzo et al. 2018). Alongside their impacts on productivity, these stresses can also affect the quality and, in turn, the marketability and end-usability of the edible portion of crops. For example, common bacterial blight (caused by *Xanthomonas phaseoli* pv. *phaseoli* and *X*. *citri* pv. *fuscans)* is a major bacterial disease in common bean (*Phaseolus vulgaris* L.) worldwide and can result in discolored, shriveled, and smaller seeds (Singh and Miklas 2015). *Magnaporthe oryzae* is the causative agent of rice blast, a destructive fungal disease responsible for between 10 and 30% of all rice yield lost annually (Fernandez and Orth 2018). Downy mildews (*Peronospora effusa* on spinach and *Bremia lactucae* on lettuce) are the most economically important diseases of lettuce (*Lactuca sativa* L.) and spinach (*Spinacia oleracea* L.) worldwide and have become particularly devastating with the advent of high-density plantings for baby leaf greens since the mid-1990s. Infection results in chlorosis and reduced yields of leaves resulting in non-marketable fresh product that can compromise (e.g., by increasing spoilage in) whole fields (Kandel et al. 2019). Molecular genetic understanding of resistance to these diseases is advancing rapidly (Singh and Miklas 2015; Parra et al. 2021; Bhattarai et al. 2020; Kandel et al. 2020). Technologies such as marker-assisted selection and prediction of breeding values based on genotypic and/or objective phenotypic data that can be applied cost-effectively in breeding programs are essential to combat these rapidly evolving pathogens. Recent advances have also been made in artificial intelligence (AI)-enabled, field-based symptom detection for common bacterial blight and four other major diseases of common bean (Gomez et al. 2024).

AI refers to the simulation of human intelligence by machines and includes various subfields of machine learning (ML) such as tabular data modeling, time-series forecasting, computer vision, natural language processing, expert systems, and robotics. In the context of breeding, modeling, and sensing, AI methods are increasingly used to analyze large, multi-modal datasets, automate feature extraction, and support decision-making pipelines by increasing the throughput and number of traits. For example, ML approaches are commonly applied for classification (e.g., of categorical traits) and regression (e.g., of continuous traits), while computer vision techniques enable trait or feature extraction from image-based sensing platforms. Most ML approaches used by breeding programs are supervised models and therefore require human labeling of training sets, which can be a bottleneck. Generative models, often unsupervised, are increasingly being explored to simulate plant traits and environments, bolstering predictive tools by augmenting training datasets and providing an efficient route to overcome the labeling bottleneck.

In this review article, we examine opportunities to integrate AI-enabled sensing and crop modeling into molecular breeding pipelines for both categorical and continuous traits, enhancing the accuracy and efficiency of trait prediction and germplasm selection and thereby accelerating crop improvement. Based on the breeder’s equation (Eq. 1), this acceleration could take place through higher selection intensity, higher selection accuracy, evaluation of crop genotypes with higher additive genetic standard deviation, decreased cycle time, or simultaneous selection on a fuller set of priority traits while minimizing tradeoffs. We will first discuss strategies for stress response and challenges in breeding for quality traits and provide overviews of the crop systems of focus for this review and status of -omics tools therein, and proximal remote sensing and crop modeling. We will then discuss the status of leveraging advances in sensing in breeding and genomics, the status of crop modeling in the focal crops, and the status of using environmental data and crop growth models in genomic prediction. We will discuss analytical (hardware and software) platforms being deployed for various nutritional quality and physiological traits, including at the level of canopy structure and function. We also discuss an implementation of AI for the discovery of novel disease resistance genes. Finally, we will discuss status and expected trends in the integration of molecular breeding, crop modeling, and sensing and downstream applications of these integrated frameworks in applied breeding.

1 $$R = \frac{{ir\sigma_{A} }}{t}$$

where *i* = selection intensity, *r* = selection accuracy (correlation between true and estimated breeding values), $\sigma$_*A*_ = additive genetic standard deviation, and *t* = number of years per cycle.

### Strategies for stress responses and evaluation thereof

Resistance to microbial infection is a genetically encoded biotic stress response. Stress response strategies typically discussed for abiotic stresses include avoidance, or evading the stress—e.g., by completing a certain critical developmental stage prior to stress onset—and resilience, or recovering from a stress event (Xu et al. 2006). Definitions of resilience in biological systems were discussed in Thorogood et al. (2023) and Zampieri et al. (2020); common themes were the ability (e.g., through physiological processes) to maintain essential function and essential internal structure through a stress event and recover afterward. Tolerance to these stresses could be defined as the extent to which yield is maintained when a crop, or genotype thereof, is subjected to a given stress for a specified duration (Cattivelli et al. 2008). Tolerance thus ultimately relates to stability of a trait across two or more levels of a given stress, including a control level absent of that stress. These levels would ideally be tested in the same field environment to control other experimental variables such as soil type, weather, and planting date.

The yield and quality outcomes of resistance, avoidance, and resilience strategies would also ultimately be captured in the same metrics used to assay crop stress tolerance. However, experimental designs and sensing/modeling workstreams that distinguish among these strategies can provide additional insight into specific mechanisms of tolerance in a given genotype (G), environment (E), and management (M) scenario. Further dissection of these mechanisms would benefit from the examination of crop structural and functional traits at multiple informative time points during a crop growing season, to examine biomass accumulation and partitioning, composition of the edible portion of the crop, and responses of these emergent properties (arising from the main and interaction effects of G, E, and M) to stress and other yield-limiting factors.

Given the major impacts of biotic and abiotic stresses on both yield and quality, stress tolerance has been a consistent target in molecular and physiological breeding efforts. A target population of environments (TPE) is the set of geographies for which a given breeding program is developing crop varieties. Managed stress environments (MSEs) are strategically situated field sites in which one or more stresses can be applied in a controlled manner with higher frequency than experienced in the TPE and can inform breeding efforts for stress tolerance (Cooper et al. 2014, 2016; Beaver et al. 2020). Crop models—once appropriately parametrized—could provide a way to integrate data from MSEs and from multi-environment trials in the TPE (Diepenbrock et al. 2021). Consideration of combined stresses will also continue to be critical for current and future TPEs (Rivero et al. 2021; Suzuki et al. 2014). Examples of less frequent but catastrophic stresses include severe droughts in 1988 and 2012 (Boyer et al. 2013; Gaffney et al. 2015) and a derecho in 2020 (a long-lived windstorm associated with severe thunderstorms; Barten et al. 2022), both in main Maize-growing regions of the USA. These stress events provided an opportunity to test recently developed germplasm and integrations with crop modeling for drought tolerance and short stature (the latter for tolerance to derechos) under more extreme scenarios, and on a larger scale given that several field research sites that are not typically stress environments were impacted by these events. Responses to emerging stresses or strains thereof, and the design of field-based strategies to evaluate them, will be important as vector and pathogen life cycles and their geographic distributions (among other aspects) may be altered under changing climates (Newton et al. 2011; Elad and Pertot 2014; Singh et al. 2023).

### Current challenges in breeding for quality traits

A central challenge in breeding programs is the identification of crop varietal candidates, parents, and/or crosses that have the best combinations of multiple traits. Addressing this challenge involves two key steps: (1) Evaluation of all candidate lines for all target traits. Trait quantification is a key bottleneck in breeding, and sensing and modeling approaches hold tremendous potential for in silico evaluations that enable both direct and indirect quantification—with continued phenotyping to validate and update the relevant models. (2) Definition of a total breeding value score to make selections, once having evaluated all target traits. This score must account for correlations among target traits and how these traits jointly determine total value.

In breeding programs that routinely evaluate a small number of clearly defined target traits, such as yield and disease resistance, these targets are jointly selected through methods such as truncation selection (identifying candidates that meet a minimum requirement for each trait individually) or use of predefined selection indices that use a linear, sometimes weighted combination of the individual traits as the final score (e.g., Crossa et al. 2025). However, both of the key steps described above are challenging for complex targets including certain quality traits (e.g., those related to nutritional quality, processability, and/or consumer preference). Namely, quality trait quantification is challenging because the relevant biochemical compounds and/or food scientific properties are typically expensive to measure, limiting the number of candidate lines that can be directly evaluated. Additionally, most existing whole-genome prediction (WGP) methods are not designed to make predictions for multiple traits that might be correlated. Definition of total value is also challenging because we often lack sufficient data to derive accurate selection indices for high-dimensional targets, particularly when they interact with other targets (such as yield and shelf life).

Genomic and phenomic technologies have continued to advance and are becoming common facets of breeding programs. While often analyzed in the context of productivity and other agronomic traits thus far, genomic and phenomic data streams also tend to contain abundant information of relevance to quality traits. However, groundtruth data for quality traits tend to be lacking, particularly those related to nutritional quality, which may not yet be valorized in the supply and/or demand streams. Development of indirect proxies that could be validated via readily accessible wet chemistry services or kits (on a subset of plots) would open up the use of sensing as a method for routine quantification among more breeding research groups by reducing the need for specialized quantification platforms and the time and cost per plot. Notably, the time involved in sensing often scales less than linearly with the size of a given field trial (Parker et al. 2020). As such, scaling of sensing methods to entire populations (e.g., to enable genetic mapping and/or genomic prediction) and/or multiple levels of a given stress could be more feasible than scaling of pure wet-chemistry methods, provided that appropriate analytical pipelines are in place for processing of the sensing data.

An understandable tendency of plant breeders and others deploying sensors is to investigate how the traits they routinely measure in the breeding program can be directly or indirectly assessed. However, some of those traits are likely measured because previous technologies (or visual observations) could assess them, not necessarily because they were the best traits (or proxies for those traits; van der Bom et al. 2020). The power of sensor technologies, particularly when coupled with crop modeling, is that we can now assess traits, growth trajectories, and other components we previously could not (Chen et al. 2014; Araus et al. 2021; Gano et al. 2024). Our goal as breeders today is to reconceptualize what data we truly ‘must’ have and how we use these data in decision making, which may be fairly different from the data we are accustomed to collecting and using, and with care to avoid methodological bandwagons that may not translate into elite cultivars (Bernardo 2016). If we can think differently about what these new data streams can provide, new opportunities—particularly in targeting genotypes and more specifically, modulating the phenology of those genotypes—to better fit changing climates in the TPE will be possible.

While grain legumes, rice, and leafy greens are examined as case studies herein, a broader set of crops is of course relevant to the improvement of food and nutritional security under changing climates. van Zonneveld et al. (2023) analyzed 138 species across a wide range of crop types (including legumes, leafy greens, cereals, fruits, seeds and nuts, and roots, tubers, and bananas), and prioritized 58 species based on complementary micronutrient profiles and coverage of production areas predicted to be still-suitable or unsuitable for production of maize, rice, cassava, and yams by 2070. Prediction into a large number of crop species and environmental contexts could benefit from foundation (i.e., pre-trained) models so that each prediction task does not start from a new model architecture and coding thereof, and so that standardized benchmarks are built into the modeling framework. Joshi et al. (2023) demonstrated the potential for pre-trained models to reduce labeling requirements and boost model performance in unseen agricultural computer vision tasks using AgML (Project-AgML 2024) which contains 39 centralized and standardized datasets with labels. However, while adaptation and translation of modeling and sensing technologies from more to less extensively studied crops with similar plant and/or inflorescence architectures could be a partial solution (e.g., in a comparison of predictive models), there are also clear benefits to de novo development and deployment of breeding technologies and predictive models in these crops by researchers who work in primary regions of production and consumption of these crops (Lasdun et al. 2024).

### Overview of the crop systems of focus

Grain legumes, rice, and leafy greens are examined here as case studies because they are important for food, nutritional, and economic security on a global scale (Ebert 2014; van Zonneveld et al. 2023). Common bean is the most important staple grain legume for direct human consumption (Gepts et al. 2008; Broughton et al. 2003). The seeds (or grains) of common bean provide protein, dietary fiber, and mineral nutrients, albeit with moderate to extensive natural variation for these traits across cultivars and environments (Katuuramu et al. 2018; Hummel et al. 2018). Common bean is examined herein due to its important role as a protein source in human diets and given that grain is the tissue type primarily consumed by humans, which enables examination of relationships between vegetative and reproductive tissues and the challenges that these relationships present for crop modeling and sensing approaches.

Rice is among the most important food crops globally, constituting 20% of global caloric intake, and serving as a primary food source for over half of the world’s population (Bin Rahman and Zhang 2023; Khush 2005). Approximately 90% of rice is produced and consumed in Asia, accounting for 35–75% of calories consumed in this region (Food and Agriculture Organization of the United Nations 2023; OECD and Food and Agriculture Organization of the United Nations 2023; Khush 2005). Prior to consumption by humans, rice seeds are milled, with brown rice retaining the germ, bran, and endosperm, while white rice is stripped of all but the starchy endosperm. Nutritional quality varies greatly depending on processing, as proteins, fats, vitamins, and minerals are concentrated in the bran and germ, with additional variation observed across varieties and cultivation conditions (Dhillon et al. 2018; Ferrari et al. 2022; Saleh et al. 2019). However, in general, rice provides mainly carbohydrates, with modest protein and trace levels of vitamins and minerals unless enriched (e.g., via biofortification or in the processing stage). Given its central role in food security and genetic tractability, rice is well positioned for exploring how integration of crop modeling and sensing approaches can enhance breeding.

Lettuce and spinach are widely consumed leafy greens that have short growing seasons of three to ten weeks. Lettuce is produced on substantial acreage on nearly all continents, whether leafy (heading or non-heading) or stem types (Mou 2008). Spinach is grown on less acreage, but production of baby-leaf spinach for fresh-market consumption has been increasing in the USA and worldwide (Davis and Lucier 2021; FAOSTAT 2024). Lettuce and spinach have a broad array of shapes, colors, and textures, which define market types (e.g., romaine, butterhead, red/green leaf, and iceberg types in lettuce; and savoy/semi-savoy/smooth types in spinach). These crops are rich in vitamins, other primary and specialized metabolites such as phenolic compounds, and mineral nutrients with human health benefits, albeit with moderate to extensive natural variation among market types (lettuce: Zhang et al. 2020; van Treuren et al. 2018; Yang et al. 2018; Damerum et al. 2015; Bunning et al. 2010; spinach: Qin et al. 2017; Koh et al. 2012; Shohag et al. 2011; Howard et al. 2002; Kidmose et al. 2001). Lettuce and spinach are examined herein given that their leaves are directly consumed by humans. Additionally, their rapid growth periods and relatively simple plant architectures (or growth habits) enable ready examination of crop modeling and sensing opportunities pertaining to both nutritional quality and physiological traits.

### Status of -omics tools for use in molecular breeding

The term ‘-omics’ is used to encompass genomics, transcriptomics, proteomics, metabolomics, and other emerging areas such as epigenomics; ionomics and phenomics are also at times incorporated (Yang et al. 2021b). Each of these subfields represents (and allows the assaying of) a layer of biological organization from phenotype to genotype or vice versa—corresponding to forward or reverse genetics, respectively. Of these layers, genomics (and increasingly, phenomics) are most often used in applied breeding, and will be focused on hereafter. However, the intermediate layers between these two have been useful for more accurate and efficient identification of candidate genes and for improved mechanistic understanding at the whole-plant, organ, tissue, and/or cellular levels (Baxter 2020; Langridge and Fleury 2011; Wallace et al. 2018; Wainberg et al. 2019; Mahmood et al. 2022; Li et al. 2024a). These intermediate layers could be particularly useful in understanding mechanisms underlying traits considered to have long ‘phenotypic distance’, defined by Hammer et al. (2019) as complex traits arising from the integration of many effects across scales of biological organization. These intermediate layers can also inform the deployment of genetic markers and phenomic proxies and (more generally) the approaches taken to breeding for nutritional quality (which often involves biochemical pathways) and tolerance to individual or combined stresses (which can differentially affect gene expression; Suzuki et al. 2014; VanBuren et al. 2024). Existing and emerging AI methods can be used to identify targets for gene editing (Farooq et al. 2024), predict functional effects of nucleotide substitutions (Wang et al. 2022), and/or predict protein structure (Jumper et al. 2021). Underlying and providing a connective thread through many of these layers are the high-quality genomic resources that are critical for molecular breeding.

Substantial advances have been made in common bean -omics over the past decade (Hu et al. 2025a, b). The release of two common bean reference genomes (one from the Andean genepool, Schmutz et al. 2014; and one from the Mesoamerican genepool, Vlasova et al. 2016) were major milestones that built upon previous efforts (McClean and Raatz 2017; Assefa et al. 2019). OAC Rex was sequenced contemporaneously due to its resistance to common bacterial blight (Perry et al. 2013). Additional sequences for common bean and other *Phaseolus* species are now available on Phytozome (https://phytozome-next.jgi.doe.gov/; Goodstein et al. 2012) and the Legume Information System (https://www.legumeinfo.org/taxa/phaseolus/; Dash et al. 2016), and a new common bean reference assembly was recently released (https://phytozome-next.jgi.doe.gov/info/Pvulgaris_v2_1). These genomic resources have provided a foundation for identifying genetic underpinnings of several trait sets including disease resistance and drought tolerance. Schmutz et al. (2014) also conducted pooled resequencing of 60 wild and 100 cultivated accessions representing Mesoamerican and Andean subpopulations, and Vlasova et al. (2016) included a transcriptome atlas, as did O’Rourke et al. (2014). Transcriptomic approaches have been employed in domesticated vs. wild accessions (Bellucci et al. 2014) and under biotic and abiotic stress conditions, contributing to breeding programs aimed at improving stress resilience in beans. Population genomics (Cortinovis et al. 2020), pangenomics (Cortinovis et al. 2024; Wang et al. 2025), and comparative genomics (e.g., Garcia et al. 2021; Moghaddam et al. 2021) investigations have also been carried out particularly among *Phaseolus* species, and synteny with other legumes including soybean and cowpea has been examined. Proteomic studies have also provided new insights into the mechanism of complex traits. For example, Zadražnik et al. (2013) examined protein profiles under drought stress, and found that proteins involved in photosynthesis (oxygen evolving enhancers) showed most contrasting abundances across treatments. Badowiec and Weidner (2014) examined and compared protein alteration under chilling stress conditions. Parreira et al. (2016) investigated the proteome dynamics of seed development. Comparative proteomic studies have also been conducted to identify different biotic and abiotic stress responsive proteins (Torres et al. 2007; Lee et al. 2009; Salavati et al. 2012; Castaneda-Saucedo et al. 2014).

Lettuce is amenable to classical and molecular genetic analyses. The seed-to-seed generation time is usually three to five months, allowing multiple generations each year. Ultra-high-density genetic maps are available for several recombinant inbred line populations that have been distributed to the lettuce genetics community. CRISPR-Cas-mediated gene knockouts can be readily achieved in lettuce (e.g., Bertier et al. 2018). Genetic resistance is available to most, although not all, of the wide range of pathogens that afflict lettuce. The genetics of resistance to downy mildew have been studied for over 100 years, and more than 50 major genes and sources of resistance are known as well as Quantitative Trait Loci (QTL) for resistance (summarized in Parra et al. 2016). Molecular marker-assisted breeding, particularly for disease resistance, is a major activity of seed companies. The molecular basis for horticulturally important developmental traits, such as bolting, color, and leaf shape as well as disease resistance are increasingly well understood (e.g., Su et al. 2020; Han et al. 2021; An et al. 2022). The 2.6 Gb genome of *L. sativa* cv. Salinas has been sequenced and assembled into nine chromosomal super-contigs that have been validated genetically (Reyes-Chin-Wo et al. 2017); a near-gapless, annotated reference genome is available from Genbank (Lsat_Salinas_v11 assembly; https://www.ncbi.nlm.nih.gov/datasets/genome/GCF_002870075.4/). Multiple other genomes have been short-read sequenced, including 445 lines of a core *Lactuca* germplasm set (Wei et al. 2021), and pangenomic studies of lettuce germplasm have started (Workum et al. 2024). The recent availability of long-read technologies is resulting in the generation of near-complete assemblies for multiple lettuce types and wild relatives. Consequently, extensive genomic tools are now available and ready to be exploited for lettuce improvement.

Both because of its smaller market size and its dioecious, outcrossing nature, spinach has lagged behind lettuce and other major crops in -omics tools. However, several genomes of cultivated spinach (*S. oleracea*) have been published (Xu et al. 2017; Cai et al. 2021; Hirakawa et al. 2021; Hulse-Kemp et al. 2021) as well as several genomes for its Y-chromosome (Ma et al. 2022). Recently, two related species, *S. turkestanica* and *S. tetrandra* were also sequenced, with data suggesting both species had contributed downy mildew resistance genes to cultivated spinach (She et al. 2024). Two databases collecting and curating spinach genomics-related information have been developed: SpinachDB (Yang et al. 2016) and SpinachBase (Collins et al. 2019). A telomere-to-telomere genome assembly of *P. effusa* has also been developed showing the genome to be larger than previous sequencing assemblies have found, and showing considerable synteny to related oomycete species (Fletcher et al. 2022). Numerous transcriptomics studies have been conducted in spinach; e.g., under downy mildew pressure (Kandel et al. 2020), high temperature (Yan et al. 2016), nitrogen stress (Joshi et al. 2020), and insect herbivory (Pamplona et al. 2022) or comparing cultivated and wild species (Xu et al. 2015), among others. Single marker assays for resistance genes or major QTL are routinely used in breeding programs. Individual markers have been developed for several downy mildew genes (Feng et al. 2018), and genotyping-by-sequencing data have been used to assess genetic diversity (Shi et al. 2017) and conduct genome-wide association studies for various traits (e.g., white rust resistance (Shi et al. 2022), bolting, leaf traits (Cai et al. 2021), nitrogen related changes in biomass (Joshi et al. 2022) and nutritional traits (Ji et al. 2024b). The vast majority of genome-wide association studies in spinach have been on germplasm collections, resulting in a low proportion of phenotypic variance explained by models (< 10%). This is due to the outcrossing nature of the species with accessions representing heterogeneous mixtures of heterozygous genotypes; therefore, substantial heterogeneity exists within each accession. Despite this, 434 individual genotypes from the USDA germplasm collection and commercial cultivars have been sequenced as a source of sequence diversity (Bhattarai et al. 2022) to begin to develop whole genome tools. A more focused effort to sequence stable (inbred) and relevant breeding germplasm must be pursued to develop effective genomic-assisted breeding tools for public breeding programs. Objective phenotyping platforms must also accompany these tools to efficiently advance breeding cycles for the multitude of qualitative and complex traits related to productivity, responses to biotic and abiotic stresses, and quality.

Rice has long served as a model for cereal crops due to its relatively small genome coupled with extensive resources such as the 3,000 Rice Genomes Project (The 3,000 Rice Genomes Project 2014) and sequenced mutant populations (Jain et al. 2019; Li et al. 2017a; Kumar et al. 2024). Furthermore, the variety Kitaake has a fairly compact growth habit, is easy to transform, is amenable to genome editing and has a rapid generation cycle (Jung et al. 2008). Additionally, findings from rice studies are more easily translatable to other monocots than those from model plants such as Arabidopsis (Borrill 2019). Molecular genetic approaches in rice have been recently reviewed (Wang and Han 2022; Xu et al. 2021; Spindel and Iwata 2018), as well as multi-omic approaches for quality traits in rice (Anacleto et al. 2015).

### Overview of crop modeling

Crop models enable the prediction of crop behavior as a function of environmental conditions and management actions. Although potential applications of crop models greatly vary, the most common objective is to predict crop biomass and yield resulting from historical or hypothetical weather conditions and particular management practices. A wide range of modeling approaches have been used to mathematically describe these interactions depending on available computational resources, input and validation data, and the desired level of physical realism. Statistical modeling approaches, including AI models, are entirely data-driven, and rely on variation in the data to describe interactions between variables (Hu et al. 2024). This class of models is usually limited by the quantity and quality of available calibration data, which can be a substantial limitation in the context of crop modeling given the extremely large number of interacting variables that should ideally be factorially varied (Lobell and Burke 2010). On the opposite end of the spectrum are mechanistically based models that leverage theoretical understanding of governing biophysical processes to describe relationships between variables (Dale et al. 2021; Allen et al. 2005; Vos et al. 2010; Bailey 2019). The advantage of this approach is that less calibration data are typically needed because many relationships are described based on physical laws and conservation equations, which also tends to lead to greater success in out-of-sample prediction given that the models are physically constrained and can include representations of important feedbacks (Sinclair and Seligman 1996). The primary limitations of this approach are that they often require detailed input parameters that may not be known, theoretical knowledge governing the complex behavior of living organisms is highly incomplete, and they may also require high computational resources. The most widely used approach for traditional crop modeling focused on biomass and yield prediction is process-based modeling, which is a compromise between the above-mentioned extremes (Jones et al. 2003; Keating et al. 2003). Process-based models represent the dynamic process of crop development by integrating differential equations describing the rate of crop development. They may or may not explicitly represent biophysical processes such as photosynthesis and transpiration, but instead often utilize empirical relationships to modulate the development rate (Holzworth et al. 2014). Due to this empirical basis, they generally require a large amount of calibration data that can take many years to amass, but are relatively straightforward to apply and often give reasonable predictions.

### Overview of proximal remote sensing technologies

**Fig. 1:**
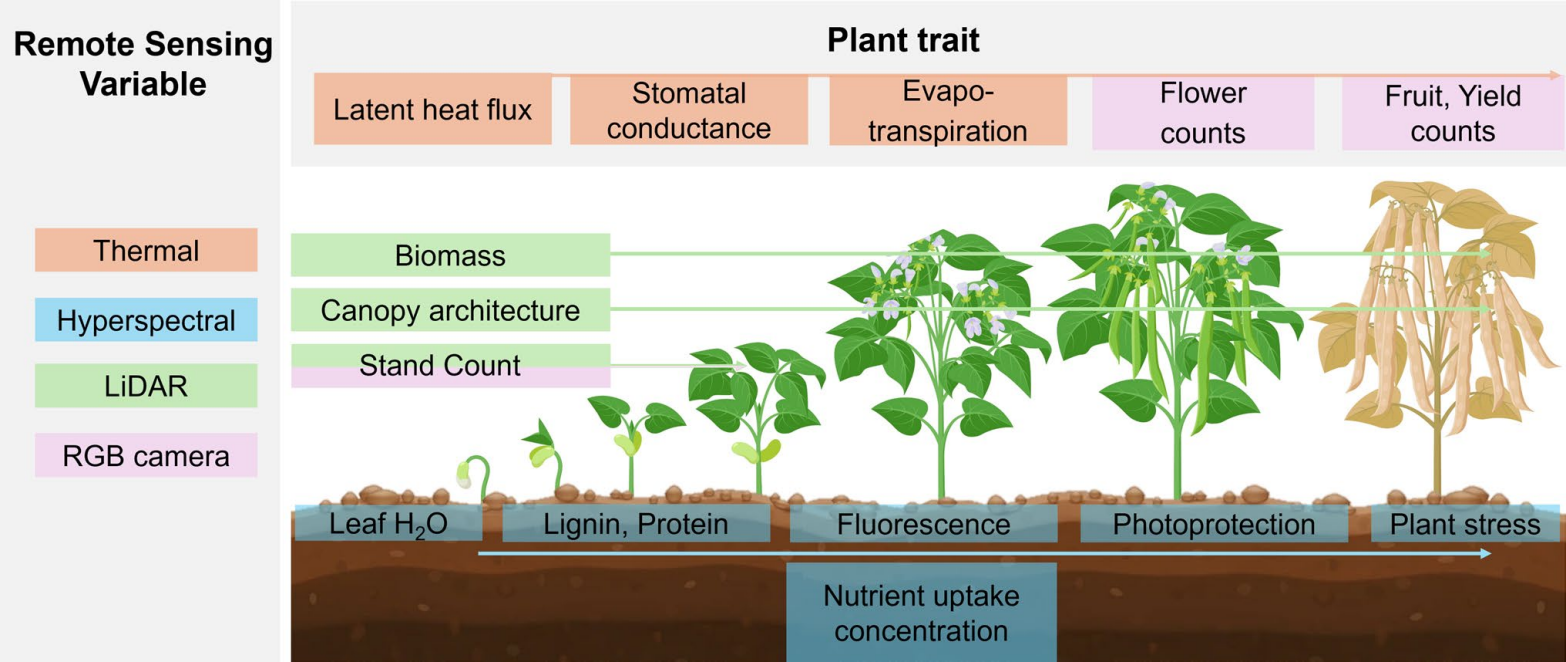
Schematic of how different remote sensing techniques could be applied to quantify plant structural and functional traits. Boxes are color-coded to indicate whether the trait would be measured from thermal (orange), hyperspectral (blue), LiDAR (green), or RGB camera (pink)and[color figure online]

Proximal sensing involves monitoring of leaf-, plant-, or canopy-level traits using handheld instruments, ground vehicles, or UAVs, and less commonly, tower-based systems (Pierrat et al. 2025), gantries (Li et al. 2021a), or spidercams (Bai et al. 2019). Remote sensing encompasses both proximal methods and those using sensors on higher-altitude platforms such as aircraft or satellites, enabling characterizations on a larger spatial scale. Sensing instruments commonly used in the assessment of priority traits include passive sensors (those that depend on solar radiation as a light source) such as RGB, multispectral, hyperspectral, solar-induced fluorescence (SIF) and thermal instruments (Wong 2023b), as well as active sensors (those that emit an independent source of energy) such as Light Detection and Ranging (LiDAR), Synthetic Aperture Radar (SAR; Liu et al. 2019), and leaf-level spectrometers. In this article, we use ‘fidelity’ to refer to the exactness (precision and accuracy) with which a plant’s structural and functional properties are captured, and ‘efficiency’ to refer to the time and cost involved in routine deployment. Proximal sensing improves fidelity by mitigating environmental noise (atmospheric absorption and other background artifacts, as observed from aircraft or satellite) in measurements, and providing increased spatial resolution, thereby offering more robust evaluations of plant physiological and structural properties (Gamon 2015; Pierrat et al. 2025). Sensors also differ in their sensitivity to specific traits, requiring appropriate instruments (spectral range/resolution) to characterize structural, biochemical or physiological traits. For example, hyperspectral data are highly sensitive to a wide range of biochemical and biophysical properties: the visible spectrum responds to pigments (Ustin et al. 2009; Blackburn 2007), the near-infrared spectrum to structural components (Homolová et al. 2013), and the short-wave infrared spectrum to water status (Angel and Shiklomanov 2022). In contrast, LiDAR or RGB-derived point clouds (photogrammetry) are sensitive to structural traits, while thermal imagers are sensitive to canopy temperature (Fig. 1). In order to assess priority traits using sensing approaches, the informative portions of sensor data must be identified (Wong 2023b). Feature extraction is the process of deriving meaningful information from raw sensor data, such as spectral reflectance or point clouds, to quantify specific plant traits.

## Leveraging advances in sensing for breeding and genomics

Sensing approaches could be used to assay intermediate traits that provide insight into—and help to simulate and predict, optionally in crop modeling frameworks—the accumulation and retention of yield (and in some cases quality) of the edible portion of the crop throughout the growing season (Magney et al. 2016). Simultaneous crop modeling and sensing capabilities are not yet routinely deployed in breeding programs for quality or physiological traits, nor for yield or stress tolerance (Peng et al. 2020). Sensing and modeling of crop yield and quality could help isolate, identify, and predict the effects of one or more stresses—for example, factor(s) predominantly controlling the rate and extent of loss in green leaf area in a given GxExM scenario (Wang et al. 2019; Großkinsky et al. 2018). The main uses of sensing data in applied breeding programs thus far have been to 1) use sensing-derived traits (or extracted features) in direct or indirect selection or to 2) use hyperspectral data (prior to feature extraction) in phenomic prediction, often in comparison and/or combination with genomic prediction. While not discussed further, a phenome-wide, genome-wide association study was also conducted to better understand how crop genomes give rise to observed phenomes, and rediscovered classical mutants in maize and known loss-of-function phenotypes in Arabidopsis (Liang et al. 2020); such approaches could continue to be useful as sensing becomes mainstream.

Conventional plant breeding programs have largely relied on phenotypic selection whereby manual measurements are used to quantify and characterize variation in plant traits including under field and laboratory conditions. While historically this method has been effective, it is time- and resource-intensive, limiting the number of unique genotypes (and plots) that can be evaluated and the genetic gain that can be made per breeding cycle (Furbank and Tester 2011; Cobb et al. 2013). However, as proximal sensing methods have become more common in breeding programs, new phenomics datasets that are information-rich can now be used for selection and advancement of promising genotypes (Araus and Cairns 2014; Araus et al. 2018; Crain et al. 2018; Tao et al. 2022; Yoosefzadeh-Najafabadi et al. 2023). Crop sensor data can provide criteria for direct selection, such as when sensors explicitly quantify the target trait (e.g., plant height), or for indirect selection when sensors are used to estimate a correlated trait (e.g., canopy volume as a proxy for total above-ground biomass) (Smith et al. 2021). Sensing-derived traits could notably be included in selection indices alongside manually measured traits, where their use conforms to many of the same theoretical and practical considerations as use of manually measured traits, such as the need to account for the correlations and heritability of all traits included in an index (Lopez-Cruz et al. 2020).

Crop sensor data can provide replacement traits (new ways to measure traditionally measured traits such as plant height and lodging resistance, discussed below). Replacement traits are useful as they can be faster to measure (higher-throughput, enabling the assessment of more genotypes with higher additive genetic standard deviation or application of higher selection intensity) or have higher heritability (and relatedly, selection accuracy) than target traits. The outcome of either of these benefits is higher rates of genetic gain and population improvement. Further, replacement traits enable breeders to collect repeated measurements that can be averaged to produce more robust estimates of true genetic values with fewer replicates or tested environments (Lane and Murray 2021). Crop sensor data can additionally provide new traits related to structural and functional plant properties that were not previously accessible. For instance, these new traits include spectral signatures that can reveal plant canopy properties (nitrogen content, anthocyanins, etc.) as well as dynamic traits captured by temporal phenotyping such as growth rates or response to changes in environmental conditions (e.g., Campbell et al. 2015; Clark et al. 2011).

### Case study: sensing of plant height and lodging resistance

Plant height is a partial determinant of lodging resistance and machine harvestability and can impact harvest index in certain crops as demonstrated by the use of dwarfing genes in the Green Revolution to reduce plant stature (Liu et al. 2018; Ferrero-Serrano et al. 2019). Drone-based imagery and resulting photogrammetrically generated point clouds are routinely used for the development of canopy height models (CHMs) from which measurements of individual and plot-level plant height can be extracted. Generally, CHMs are derived by calculating the mathematical difference between crop canopy height, often as point clouds interpolated into 3-D surfaces representing a continuous distribution of height throughout the crop canopy, and bare soil digital surface models. While studies evaluating lodging susceptibility with CHMs are still lacking in pulse crops (Madurapperumage et al. 2024), a few methods have been applied to estimate plant height using CHMs in common bean. For example, Parker et al. (2020) and Arriola-Valverde et al. (2020) estimated canopy height using photogrammetry applied to UAV data collected at a few to several periods throughout the growing season while other studies have estimated canopy height using terrestrial laser scanning (El-Naggar et al. 2021). Plant height and lodging have been more routinely evaluated in rice on subregional scales (Yang et al. 2017; Zhao et al. 2019) and as means of high-throughput phenotyping in applied breeding (Weiyuan et al. 2024; Kawamura et al. 2020). In a study examining the correlation among plant height (as scored with UAVs), management practices (N fertilization), lodging incidence, yield (and its components), and rice quality, Wu et al. (2022b) found that N fertilization level and its interaction with water management significantly impacted lodging, rice amylose and protein content and palatability in one of two years. The use of sensor-derived plant height measurements demonstrates another advantage of proximal sensing data, namely precision and accuracy of quantification often exceeding those of manual measurements, which could reduce measurement error and bias in plant phenotyping thereby making selection more effective. Zang et al. (2023) estimated plant height of wheat with several sensing platforms and ground-based measurements (measuring poles) and found that sensor approximations of plant height, particularly those estimated from terrestrial LiDAR, had significantly higher heritability estimates than human-derived measurements. Another advantage of sensor-derived measurements would be the feasibility of quantifying plant height at multiple time points (e.g., as Mu et al. 2022 found to be valuable in genetic, environmental, and developmental analyses), particularly if also regularly deploying field-based sensing platforms for other traits.

### Case study: estimation of early vigor from sensor imagery

Early vigor is defined as rapid plant establishment and growth occurring soon after germination, and is of interest for suppression of weeds, reduction of water loss from soil evaporation, and efficient capture of solar energy. Early vigor can be estimated from nadir multispectral and RGB imagery commonly collected via drone platforms. Vegetation indices (VIs) such as normalized difference vegetation index (NDVI) or excess green index (ExG, Woebbecke et al. 1995) can be applied to segmented plot areas, effectively calculating green cover across the plot area. Alternatively, plot-level imagery can be classified into binary categories delineating soil and plant areas using thresholding based on a greenness index (Xiao and Moody 2005). The classified imagery can then be used to quantify vigor as the portion of plot area that is occupied by green pixels in the image set. Additionally, if sensing is performed at multiple time points, vigor can be quantified as a growth rate indicating the change in VI score or green pixel area per unit of time, corresponding to the change in canopy cover during the early portion of the season (e.g., Magney et al. 2016).

### Proxy traits for targets that are challenging to quantify directly

While certain traits such as biomass in reproductive structures or nutritional composition cannot be directly quantified using current field-based sensing techniques due to occlusion (e.g., seeds being contained inside of pods in the canopy) or lack of a direct measurable counterpart, they can be targeted in selection schemes by selecting on proxy traits to achieve genetic gains. For example, Li et al. (2023) predicted grain protein content in rice using canopy multispectral imagery, VIs derived therefrom, and ML models (random forest and XGBoost). They found predicting grain protein content from VIs that primarily represent the plant canopy was more effective when additional intermediates, or second-order proxies—such as leaf nitrogen traits—were included, which were recombined in the final predictions of grain protein content. Magney et al. (2016) also investigated the predictive power of VIs for monitoring grain protein, yield, and biomass in wheat and while they found that daily NDVI values were generally poor predictors of these traits, promising predictions were made with these data once used to delineate fine-scale phenological intervals. These approaches illustrate how high-temporal-resolution phenotyping can enable the dynamic examination of source-sink relationships and carbon and nitrogen remobilization during the vegetative to reproductive transition.

Using proxy traits requires careful consideration of their reliability across GxExM scenarios. The strength of correlation between proxy and target traits may degrade across different genotypes, environments, and sensing conditions (Wasson et al. 2012). Similarly, updates to sensing platforms or operations can introduce variation that impacts the validity of proxy traits. These considerations parallel the need to update genomic prediction models as cycles of selection shift population structures and prediction accuracies (Neyhart et al. 2017; Voss-Fels et al. 2018; Chiaravallotti et al. 2024) and highlight the importance of coupling sensor-based indirect selection with robust model retraining and validation strategies, particularly when working with rapidly evolving populations and heterogenous sensing conditions. To ensure accuracy in indirect selection, validation of proxy trait correlations is necessary, ideally by sampling a subset of plots within each season and environment for both the proxy and target traits. Where frequent sensing changes are unavoidable, the associated validation burden may render some sensors impractical for routine deployment. Finally, although proximal sensing methods provide useful criteria for selection, they remain partial solutions. Breeding programs depend heavily on additional criteria, including genomic and manual phenotypic characterizations, to improve crop productivity and nutritional quality. As generalizable proximal sensing methods—across G, E, and M scenarios, and across species with similar growth habits—continue to be demonstrated as reliable and cost-effective means of achieving genetic gains, their use in selection will become increasingly routine.

### Applications for hyperspectral data prior to feature extraction

Hyperspectral data that are generated while sensing priority or proxy traits could have additional utility for breeding programs. For example, hyperspectral relationships could be used to identify off-type plants (or seeds) or to test for hybridity (e.g., as in Zhang et al. 2022) without needing to genotype each plant in a field plot or seed produced from a cross, which can be infeasible in many low- to medium-resourced programs (and/or in crops for which low-cost genotyping panels have not yet been developed). Phenomic prediction/selection, which is reviewed in Robert et al. (2022) as the use of spectra in prediction/selection for priority traits, could also be carried out whether on spectra of seeds (e.g., via benchtop near-infrared spectroscopy), leaves, and/or other tissues (Rincent et al. 2018). Zhu et al. (2021) found that phenomic prediction models were less reliant than GP models on relatedness between the training and prediction sets, and were more accurate than GP models with similar sizes of training set. Hyperspectral data may not be necessary to conduct phenomic selection: for example, Adak et al. (2023) found that phenomic selection using UAV-based RGB and multispectral data can outperform genomic selection. However, care is needed when comparing phenomic and genomic prediction models, particularly when hyperspectral data are collected on the same plants as groundtruth data on priority traits. Environmental correlations between spectral and priority traits can severely bias estimates of phenomic prediction accuracy, making them appear more accurate than they actually are (Runcie and Cheng 2019). Alternatively, directly combining genomic and hyperspectral information has also shown promise in prediction contexts (Runcie et al. 2021), with advisement to consider correcting hyperspectral data for flowering time and/or maturity time to avoid indirect selection on those key traits (Krause et al. 2019).

### Current challenges in sensor deployment within breeding pipelines

The financial expense of sensors, sensing platforms, required training, and certifications is a consideration and at times can be prohibitive to crop improvement programs. Given the reality of budget constraints, programs must make purchasing decisions that balance maximizing useful output while minimizing cost (including personnel time) per data point or sample. A parsimonious strategy would be to select high-efficiency sensing technologies which achieve the desired target trait characterization with minimal time and resource inputs and that are routinely calibrated and validated against high-fidelity sensors. However, identification of the most appropriate technology is not always straightforward.

Along with cost, the scale, resolution, and throughput required by breeding programs limits potential sensor options. For example, while advances in satellite technologies and increases in the number of satellites in orbit have broadened access to high-quality remote sensing datasets, the coarse spatial resolution of publicly available satellite imagery precludes use by breeding programs which often rely on small experimental plots in early generations (on the order of one to a few square meters; though Shrestha et al. (2025) found utility for satellite data in small-plot, multi-state trials) and require data streams with fine spatial resolution for many target traits (e.g., relying upon detection of individual leaves, flowers, or pods). However, there have been efforts to increase spatial resolution of satellite and other imagery using sub-pixel mapping and ML techniques to bridge the gap across platforms and spatial scales (Smith et al. 2021). Further, future satellite missions are seeking to improve both spatial and spectral resolution, such as NASA’s Surface Biology and Geology (SBG) mission (Cawse-Nicholson et al. 2021), the European Space Agency’s Copernicus Hyperspectral Imaging Mission for the Environment (CHIME) (Rast et al. 2019), the Earth Surface Mineral Dust Source Investigation (EMIT) mission (Green et al. 2020), and initiatives by private companies such as Planet Labs aiming to expand into hyperspectral imaging capabilities.

Notably, sensing could feasibly be conducted on the same trials in which yield and stress tolerance are being evaluated. Such sensing could be used to assay biomass accumulation and partitioning, stress tolerance at additional seasonal time points (and perhaps on a more time-continuous basis), and/or to assay additional priority traits, such as certain aspects of quality. However, sensing for multiple traits in a given field trial may require multiple sensors and additional sensing campaigns (at informative timepoints for each respective trait) for optimized accuracy. Ensuring sensor and platform compatibility can also be challenging, at times necessitating separate platforms or data collection intervals. These challenges stem from various technical and logistical factors such as payload restrictions, field of view/altitude incompatibility, frame rate/velocity incompatibility, image resolution, lighting control, interference of active and passive sensors, sun/sensor geometry, and power and temperature regulation capabilities for various sensor components. Finally, biological and environmental constraints also limit routine deployment of sensing technologies. Occlusion (especially in crop species with reproductive structures that lie within the canopy), difficulties accessing distant field sites, and variable sensor performance under changing environmental conditions can all reduce data quality, reliability, and ease of use.

Selection decisions must be made quickly to inform seasonal breeding program activities such as crossing and line advancement, often in a matter of days or weeks. These data must be linked to the corresponding observation unit (e.g., plant or plot) to facilitate use in prediction models or selection indices, but in the absence of a strategically designed framework, the integration of various data streams in this timeframe is not a trivial task (Tardieu et al. 2017). The plant breeding API (BrAPI) offers a potential solution to these data integration challenges by enabling the seamless transfer of plant breeding data between data collection applications, databases, and any other compatible endpoints (Selby et al. 2019). However, while BrAPI currently supports genomic, phenotypic, and RGB image data and metadata, it does not yet include standards for many proximal and remote sensing platforms nor for other high dimensional data types such as transcriptomic or metabolomic data. Manual curation is impractical and inefficient at this scale, so other data integration strategies are limited to custom scripts. While often effective, ad hoc solutions vary in terms of scalability and completeness of documentation, introducing potential points of failure.

Ultimately, plant breeders and plant breeding programs need turn-key sensor platforms that can be set up and deployed with minimal effort, and that can collect and analyze data in a streamlined and straightforward manner so that breeders have actionable results on which to make selections. While the diversity of sensor technologies, acquisition platforms, databases, and analytic methods is desirable conceptually and certainly useful for research purposes, in reality, these variables pose challenges for deployment in breeding pipelines.

### Using artificial intelligence to improve fidelity and efficiency of sensing technologies

The recent boom in artificial intelligence, while popularly associated with large language models, has also accelerated progress in AI-enabled phenotyping through increased access to computational resources, pre-trained models, large-scale datasets, and the development of new model architectures (Sheikh et al. 2024). These developments reflect a broader diffusion of AI advances across domains—advances that offer new opportunities to refine phenotyping methods.

One such opportunity is addressing a persistent challenge in phenotyping: balancing image resolution/sensitivity with cost and scalability. Cameras that capture images at higher spatial, spectral and temporal resolution/sensitivity typically incur higher costs. High-cost, high-sensitivity cameras (typically due to larger sensor size) effectively provide better contrast/edges/etc. in images, leading to greater feature resolution thereby enabling the capture of detailed characteristics of a crop such as reproductive structures (buds, flowers, fruits). Lower-cost cameras may only capture broader features such as canopy characteristics (green cover, volume approximations).

One way that AI can contribute to breeding is by enhancing the value that investments in sensors present to breeding programs. AI-based methods can be used to enhance image quality, sharpening features and allowing for higher-fidelity characterizations utilizing lower-cost sensors, thus expanding their utility across spatial and temporal scales. For instance, Generative Adversarial Networks (GANs) can be used to enhance image quality to improve model performance when detecting or classifying traits as demonstrated by Mahadevan et al. (2024) for rice leaf blight classification. Yun et al. (2024) also utilized GANs to improve temperature resolution and image clarity of low-cost thermal imaging. Deep learning models have also enabled the derivation of different data types from a single sensor. For example, Cui et al. (2022) estimated depth using RGB cameras, achieving a balance of accuracy and speed for real-time sensing. Zhao et al. (2022) reconstructed high-quality multispectral images from low-cost RGB inputs. These advancements demonstrate how AI-based methods can significantly enhance the utility of low-cost sensors by improving data quality and expanding the range of data that could be collected, opening up new possibilities for real-time, scalable, and cost-effective solutions.

## Status of crop modeling in grain legumes, rice, and leafy greens

Crop models are used to predict crop behavior as a function of environment and management as described above, and can also incorporate their interactions with genotype, alongside genotypic main effects. Crop growth models (CGMs) specifically model crop growth and development. Here we discuss the status of crop modeling (including CGM-enabled) methods and applications for the crops of focus for this review. In the subsequent section, we also highlight key methodological advances that represent ongoing and future directions across crop types. Modeling platforms are discussed commensurately with the extent that they have been used in a certain crop. We focus herein on use cases that have involved aspects of stress response, trait prediction, and/or integration with sensing or genomic data.

A major process-based crop modeling platform, the Agricultural Production Systems sIMulator (APSIM), has been developed over decades for a suite of crop types (Holzworth et al. 2014). Another major platform, the Decision Support System for Agrotechnology Transfer (DSSAT; Hoogenboom et al. 2019), implements two families of process-based crop models, CERES and CROPGRO. The CERES models are based on radiation use efficiency and tend to be simpler. The CROPGRO models are more mechanistic (daily-timescale models focused on growth, development, and yield and balance of carbon, nitrogen, and water) and have more coefficients for specific species and genotypes.

The CSM-CROPGRO-Dry bean model is a commonly used process-based model for common bean. Boote et al. (2018) used CROPGRO to test temperature responses in sunlit controlled-environment chambers for a determinate cultivar of common bean and five other crop species. Jha et al. (2023) used CROPGRO with Coupled Model Inter-comparison Project (CMIP) 6 current and future climatic data to characterize the target population of environments (TPEs) based on drought stress patterns in East Africa using a determinate cultivar, with integration of sensitivity analysis. The TPEs they identified were lack of drought stress with some high-temperature stress (comprising the majority of the production area), terminal drought with little high-temperature stress, and intermittent drought. Heinemann et al. (2016) used CROPGRO for one determinate and one indeterminate bean cultivar in two rainfed TPEs (distinguished by wet vs. dry seasons) in Goias, Brazil. That study found terminal and/or reproductive drought stress had ~ 25% frequency in the rainfed TPEs and that planting date and use of a shorter-duration cultivar were important avoidance mechanisms in the dry season. They also noted the importance of integrating high-temperature and biotic stresses into future modeling efforts. Antolin et al. (2021) tested two future climate scenarios—using Representative Concentration Pathways (RCPs) 4.5 and 8.5 and weather data from global circulation models (GCMs)—in Goias for the same indeterminate (and a second such) cultivar in CROPGRO. Coelho et al. 2023 also identified the importance of earlier sowing date for one determinate and one indeterminate cultivar under deficit irrigation in the winter season of southeast Brazil with use of CROPGRO and identified a positive relationship between global solar radiation after flowering and grain yield at the two highest (100% and 132% evapotranspiration) irrigation levels.

Hwang et al. (2017) tested a dynamic modular QTLxE approach using a recombinant inbred (RI) family between a determinate and indeterminate cultivar with a focus on node addition rate, rate of development toward time to flowering (RF), and daily maximum number of main stem nodes. These parameters were considered linear combinations of G, E, and GxE, with RF calculated daily and flowering time determined as the sum of daily rates. Wallach et al. (2018) used the same recombinant inbred family to test a model that used the photothermal time to flowering equation from CROPGRO. They identified QTL for flowering time and for three estimated model parameters: optimal temperature for maximized developmental rate, photoperiod sensitivity (or reduction in developmental rate per hour of increase above the critical short daylength), and the reciprocal of photothermal time to flowering under optimal conditions. Vallejos et al. (2022) tested a dynamic linear mixed-effects (LMM) model (incorporating G, E, and GxE) of RF and compared it to CROPGRO, which had high but variable accuracy in this RI family—potentially due to fixed parameter values that were not accurate for all genotypes. The dynamic model outperformed the static model, which was ascribed to be due to inputting daily weather values rather than those aggregated over a longer period in the static LMM approach.

Heinemann et al. (2022) took an enviromic approach using generalized additive models (an alternative to generalized linear models that allows use of nonparametric functions as predictors via smoothing functions), environmental covariates (ECs; aggregated across the whole season or for the vegetative, flowering, and reproductive stage), and yield data from 424 variety trials to estimate optimum limits of ECs for common bean production in each growing region of Brazil. They identified ECs to include in each regional model based on stepwise selection, and in turn tested the viability of those models for use in yield prediction. Seidel et al. (2016) compared the use of two photosynthetic models within a biophysical crop model for common bean: the Goudriaan and Van Laar (GvL) model focused on light distribution and light response curves, and the Farquhar–Ball–Collatz (FBC) model focused on biochemistry and hourly leaf stomatal conductance. They found both models performed adequately under non-stress conditions but both underestimated yield under high-temperature stress. However, the FBC model performed better, which was ascribed to be due to the GvL model solely basing carbon partitioning on developmental stage without adjustment for stress factors.

Hummel et al. (2018) used the EcoCrop suitability model under RCP 6.0 (and using future weather data from GCMs) to estimate historical and future common bean yields in East Africa and found the majority of current common bean production area in East Africa will no longer be suitable by 2050 due to temperatures and/or precipitation. That study also found significant increases in protein, zinc, phytate, and lead levels and a significant decrease in iron under drought stress compared to well-watered conditions in 20 varieties grown in four trials in Malawi. Rippke et al. (2016) examined suitability for common bean and other staples under RCPs 6.0 and 8.5, and common bean was found to be the crop most in need of transformational change (with crop suitability under a viable threshold in at least 10 out of 20 years) under either RCP. Jarvis et al. (2012) also used EcoCrop and GCMs and found that beans and potatoes had somewhat similar climate sensitivities, perhaps due to projected changes in highland areas that would affect both crops. Finally, Ramirez-Cabral et al. (2016) tested two GCMs in a mechanistic (process-based) modeling approach, CLIMEX, that uses a weekly time step and from monthly weather data provides annual growth, stress, and ecoclimatic indices; the latter is used as a measure of climatic suitability. They found that many of the areas predicted to be suitable for common bean are already producing common bean, and that dry/drought stress was the factor most limiting geographic distribution but high-temperature stress was also projected to limit future distribution. Together, these studies demonstrate the diversity of modeling approaches and applications in common bean, which inform further improvement efforts for and production of this crop.

Recent crop modeling efforts in leafy greens have focused on assessing growth in controlled-environment agriculture settings by utilizing RGB imagery and depth or point cloud data captured at multiple time points during the crop growth cycle. These data have been used to parameterize nonlinear models and develop prediction frameworks based on deep learning models (Buxbaum et al. 2022; Li et al. 2022a; Ojo et al. 2024; Raja et al. 2021). In addition to these data-driven approaches, mechanistic models have been developed to understand and predict lettuce growth. For instance, Pearson et al. (1997) introduced a model that divides lettuce carbon stocks into structural and storage pools and simulates growth and maturity based on these pools, their fluxes, and the effects of environmental factors such as CO_2_ concentration, temperature, and thermal time. Similarly, Nomura et al. (2020) applied a mechanistic model to study spinach canopy photosynthesis and growth, using data from an open-type flux chamber and Leaf Area Index (LAI) estimates derived from time-lapse photography. Their findings showed a clear linear relationship between LAI and cumulative net photosynthesis, indicating a predictable connection between canopy growth and photosynthesis in leafy greens. Collectively, these efforts underscore the value of integrating diverse types of sensor data with various modeling approaches to enhance our understanding and prediction of crop growth dynamics across multiple scales.

In rice, CGMs have been extensively utilized for several decades around the globe, including early models established by the United Nations Food and Agriculture Association (FAO; Doorenbos et al. 1979), the International Rice Research Institute (IRRI; Horie et al. 1995; Li et al. 2017b), DSSAT (Buresh et al. 1991; Singh and Ritchie 1993; Hoogenboom et al. 2019; Jones et al. 2003) and RiceGrow (Tang et al. 2009). Process-based modeling has been used to study the impacts of irrigation and other management practices, biotic stressors, and climate resilience, among other use cases (Ahmad et al. 2013; Hussain et al. 2023; Wang et al. 2024; Jamshidi et al. 2024). In the USA specifically, both the CERES-Rice (Hoogenboom et al. 2019; Jones et al. 2003) and IRRI-produced ORYZA models (Li et al. 2017b) have been able to accurately simulate yield from both the California and Southern US rice breeding programs (Espe et al 2016; Li et al. 2020). Li et al. (2022b, 2024b) have gone on to simulate the effects of temperature, CO_2_, and extreme heat on rice yields in the USA. Liu et al. (2022) demonstrated that the base temperature for growing degree day (GDD) accumulation in rice is genotype-specific, with implications for phenology prediction and agronomic decisions. Onogi et al. (2016) integrated genomic prediction and a phenological model to predict heading date in untested rice genotypes and environments. Yang et al. (2022) used genomic prediction to predict model parameters for seven rice CGMs, which were then used to simulate days to flowering. Over the decades as crop modeling and sensing (and recently, machine learning) technologies have developed in tandem for rice production, these technologies have also been integrated (e.g., Huang et al. 2002; Ko et al. 2024). Jin et al. (2018) reviewed the integration of sensor data into regional field crop models for rice and other systems, highlighting potential benefits and difficulties, including sources of error and the trade-offs inherent in different data assimilation methods.

However, for the crop systems of focus herein and in general, integrating sensing and/or modeling with other data types such as genomic data introduces unique challenges. Addressing these challenges is essential to increase the robustness of integrated frameworks and more fully leverage these approaches in crop improvement. Crossa et al. (2022) reviewed the integration of genomics, phenomics, and enviromics, with utility in breeding also discussed in Crossa et al. (2021). In the following section, we will review the use of environmental data and CGMs in genomic prediction, as a critical methodological trajectory of direct relevance to breeding.

## Use of environmental data and CGMs in genomic prediction

Environmental data such as weather and soil parameters are often collected in field trialing networks and are increasingly available on regional, national, continental, and global scales with varying spatial and temporal resolution (e.g., for soil: Soil Survey Staff; Batjes et al. 2024; Calisto et al. 2023; FAO and IIASA 2023; Fischer et al. 2021; Hengl et al. 2017; Arrouays et al. 2014; Shangguan et al. 2014; for weather: PRISM Group; Thornton et al. 2021; Skamarock et al. 2019; Schaefer et al. 2007; for effects of resolution in agricultural applications: Mourtzinis et al. 2017; Hoffman et al. 2016; van Bussel et al. 2016; Zhao et al. 2015; Ramirez-Villegas and Challinor 2012). From these environmental data, individual parameters or combinations thereof have been tested for their utility in improving breeding value predictions for specific environments: perhaps namely, as covariates in purely statistical models or as inputs to CGM-enabled frameworks (Bustos-Korts et al. 2016). Key workstreams within the use of environmental data in genomic prediction (GP) have been (1) predicting and dissecting GxE; for instance, which genotypes will perform best in which environments, and mechanisms underlying differential performance; and (2) defining TPEs via envirotyping, i.e., environmental classification (and potentially regression). The incorporation of environmental covariates (ECs) and their interaction with genetic factors, whether or not these investigations incorporate CGMs, has been an important development beyond the use of solely genetic effects in GP given the influence of E and GxE on priority traits within and across TPEs. This area of inquiry was recently surveyed in Tolhurst et al. (2022) and Hu et al. (2025a, b). We will describe the concept and progression of approaches that solely use known ECs (with or without variable selection or regularization or feature engineering), approaches that solely identify factors linked with GxE that do not necessarily correspond with individual ECs, and hybrid approaches using both known and latent ECs.

Approaches that solely use known ECs assume GxE is completely determined by the provided ECs. Both fixed and random regression approaches have been employed, as detailed in Tolhurst et al. (2022). Jarquin et al. (2014) implemented the use of known ECs in GP by using a multiplicative reaction norm model to jointly capture the main and interaction effects of markers and ECs using covariance structures. Extensions and adaptations of this method were implemented by Pérez-Rodríguez et al. (2015), Montesinos-López et al. (2016), Malosetti et al. (2016), Morais Júnior et al. (2018), Monteverde et al. (2019), Westhues et al. (2021), and Costa-Neto et al. (2021b), among others. Jarquin et al. (2021) found incorporation of GxE in GP improved predictive ability but that use of naïve ECs did not further improve GxE models. Hu et al. (2025a, b) surveyed approaches for selection of ECs or construction of new features out of them, for instance using variable selection or regularization (e.g., Critical Environmental Regressor through Informed Search-Joint Genomic Regression Analysis in Li et al. 2021b) or nonlinear ML (e.g., neural networks and saliency maps in Washburn et al. 2021 and Kick et al. 2023). Montesinos-López et al. (2023 and 2024b) used feature selection (based on Pearson’s correlations or the Boruta algorithm) in the training set to select ECs for use in GP. Rogers and Holland (2022) found use of all ECs (without dimensionality reduction) to be favorable and that modeling of marker x environment effects did not improve predictions in untested environments. Montesinos-López et al. (2024a) found that feature engineering to calculate various mathematical transforms among pairs of ECs was helpful in multiple genomic and GxE-enabled prediction scenarios. Costa-Neto et al. (2023) found using environment-phenotype associations as an intermediate step akin to reinforcement learning was helpful for dimensionality reduction of the GxE kernels and capturing of reaction norm trends; they also found diversity in genotype-specific coefficients for different ECs, highlighting a challenge in identifying key ECs that consistently capture GxE across years.

Another approach has been to identify ‘factors’ underlying GxE patterns among trials that are not explicitly linked to ECs. Piepho and Williams (2024) reviewed the use of factor analytic models for prediction into the TPE and the potential transition to use of observable ECs. Rogers et al. (2021) clustered environments based on ECs (the first 10 factors from factor analysis of weather data), tested G x cluster and G x E (nested in cluster) effects, and identified ECs related to the covariance of genotypic performance across environments. Tolhurst et al. (2022) and Hu et al. (2025a, b) used a combination of known and unknown ECs, which can provide flexibility if environmental data do not contain all relevant ECs so that other GxE patterns can be learned even if not yet fully explained. Notably, new (or ‘untested’) environments are particularly relevant to breeding for prediction of future, particularly on-farm, performance. Only models that use ECs (in some form, including CGMs as reviewed below) can tailor predictions to new environments, as unknown/latent EC models can only ‘observe’ GxE in the multi-environment trial itself but cannot extrapolate it to new environments (Tolhurst et al. 2022; Piepho and Williams 2024; Hu et al. 2025a, b).

ECs have also been used in genomics-enabled examinations of germplasm adaptation and envirotyping and in training set optimization. Ly et al. (2018) used genomic random regression, or factorial regression genomic best linear unbiased predictor (G-BLUP), to predict adaptation to a stress EC calculated from monthly weather data that approximately corresponded to developmental stages. Costa-Neto et al. (2020) extracted ECs via geographic information systems, then used factorial regression for spatial interpolation by testing sensitivity of genotypes to ECs and using those sensitivities along with genotype-specific intercepts to generate yield adaptability maps. Costa-Neto et al. (2021a) took an enviromic assembly approach, using ECs to conduct envirotyping via qualitative or quantitative descriptors. Finally, Gevartosky et al. (2023) examined the use of enviromics alongside genomics in training set optimization for use in GP and found a G x EC x trait kernel to be resource-efficient.

CGMs have also been used to estimate phenology and other secondary traits as an intermediate step to GP, and/or to identify ECs linked with GxE patterns. Heslot et al. (2014) used a process-based CGM to predict developmental stages, which they then used to extract stress covariates from weather data. Those covariates were then used in factorial regression to capture part of the GxE or QTLxE interaction (by allowing genotypes or QTL to have different sensitivities to those covariates), with soft rule fit predictors to capture nonlinear responses of QTL to stress covariates, when predicting yield in untested environments. Rincent et al. (2019) used the same process-based CGM to extract ECs from weather data summarized by developmental stage, identified a subset of ECs through a stepwise forward approach based on correlation of the resulting covariance matrix with that from additive main effect and multiplicative interaction (AMMI) decomposition, and used different kinship matrices for the genotypic main effect vs. GxE interaction effect. Robert et al. (2020) used the same process-based CGM to predict a secondary trait (heading date) in a test set, which they then used as an environment-specific covariate in trait-assisted prediction for a target trait (grain yield). Millet et al. (2016) clustered environments based on temperature and water scenarios (identified via APSIM in Harrison et al. 2014, though water scenarios were expressed based on measured soil water potential in Millet et al. 2016) and tested the interactions of G by environmental classification and of QTL by a given EC. Millet et al. (2019) used APSIM to simulate light interception for a reference hybrid and identified that and two other ECs in relevant phenological stages for grain number (with no significant ECs identified for grain weight). A factorial regression model for grain number accounted for the main and interaction effects of G and E, those three ECs, and genotype-specific sensitivities to those ECs, and GP was used to predict genotypic main effects and sensitivities to ECs (and grain weight) in untested genotypes. Grain number and grain weight were then multiplied to estimate grain yield, which was benchmarked against the Jarquin et al. (2014) model.

Finally, crop growth modeling and GP have been directly integrated. Technow et al. (2015) demonstrated this integration, termed CGM-WGP, in a simulated biparental population using Approximate Bayesian Computation (ABC). They described the extensive work involved in parameterizing CGMs, the importance of unbiased priors (particularly in new environments), and the computational requirement involved in sampling. They also described the issue of non-identifiability (e.g. multiple sets of intermediate trait values giving rise to the same yield), which is alleviated if measuring/observing more intermediate traits; they offered early biomass development and LAI as examples that could be measurable with high throughput. Cooper et al. (2016) tested that CGM-WGP (via ABC) framework in an empirical dataset for lines from a single biparental cross that were evaluated in two managed stress environments that incurred drought stress at flowering. Soil depth was found to be a distinguishing factor between the two environments that was important for alignment of predicted and observed yields. Prediction accuracies were highest within environments, and lower for untested (compared to tested) genotypes. Prediction accuracies were similar to those of G-BLUP, with a potential explanation that this empirical dataset may have had less physiological epistasis and/or been less fully captured by the CGM than the simulation dataset used in Technow et al. (2015).

Messina et al. (2018) re-formulated this CGM-WGP framework to use a Bayesian generalized hierarchical model and Metropolis–Hastings within Gibbs sampling algorithm. They tested four empirical populations (with six unique parents in total) and found increased relative accuracy for CGM-WGP compared to BayesA in the water-limited (WL) environment tested and for two of the populations in the non-WL environment tested. They also simulated a population in two WL and one non-WL environment and found increased relative accuracy for CGM-WGP compared to BayesA for predictions between WL and non-WL environments. They discussed a second generation of CGMs accounting for genetic variation across scales and integration of these models with less dense phenomics (than in two-step approaches that rely on observation of intermediate traits) focused on establishment of functional relationships and informative priors. Diepenbrock et al. (2021) tested CGM-WGP in a half-diallel and found predictive ability greater than or at parity with that of BayesA in all four quadrants of prediction: both tested and untested genotypes and tested and untested environments. They also found the CGM-WGP framework was able to estimate traits relevant to yield potential and/or drought tolerance simultaneously with multiple environment types in the training step. All of these CGM-WGP studies emphasized the importance of a suitable CGM. Notably, CGM-WGP does leverage the use of ‘known’ ECs in the sense that the ECs are specified model parameters and/or are constructed by the CGM. Routine ‘observation’ of model parameters pertaining to both ECs and crop structure and function (including physiological traits) for diverse crop types, via high-fidelity and high-efficiency extraction from sensing data alongside continuous improvements in weather and soil data streams and interpolation, will be a key next step. Incorporation of crop composition/quality into these integrated frameworks would also be beneficial in a longer-term effort to simulate and predict compositional/quality traits alongside yield and phenology.

## Operational considerations for sensing of nutritional quality and physiological traits: which sensor for which trait?

### Nutritional quality traits

Routine monitoring of and selection for nutritional quality traits has been less common than for yield and stress tolerance, and remains an emerging but challenging area (Diepenbrock and Gore 2015; Fernie et al. 2006). Nutritional quality traits are herein differentiated from quality traits more generally (such as seed color uniformity and cooking time in grain legumes, leaf color and shape in leafy greens, or flavor/aroma in either crop type) in that they specifically and directly offer benefits to human nutrition. The time and cost involved in assaying nutritional quality traits can depend on the instrumentation, analytical standards, and other consumables that are appropriate for accurate quantification of the target nutrients; the number of target traits that can be assayed on the same platform rather than requiring separate platforms (or extractions, etc.); the tissue type of the edible portion of the crop; and the need for cold storage and tissue grinding capabilities.

Plant-based antioxidants, such as phenolics and carotenoids, can be degraded or turned over both pre- and postharvest due to enzymatic factors (e.g., polyphenol oxidases or carotenoid cleavage dioxygenases) and non-enzymatic factors such as light, heat, oxygen, or extreme pH. Degradation or isomerization of these compounds can eliminate or reduce (respectively) their nutritional value alongside their pigmentation (Yahia et al. 2017; Dias et al. 2014; Boon et al. 2010; Enaru et al. 2021). Optimal sample preparation methods and access to cold storage and low light conditions are critical when assaying nutritional quality traits to ensure accurate quantification with minimized degradation and turnover.

Accurate quantification of target nutrients can also vary due to genotype-specific degradation rates for antioxidants. This variability can complicate the assessment of nutritional quality traits when there are already differences in at-harvest concentrations across genotypes (examples for carotenoids: Yahia et al. 2017; Mou 2005). Pandjaitan et al. (2005) assessed spinach leaves harvested at three maturity stages from eight commercial cultivars and eight advanced breeding lines and found significant effects for G and the G x maturity interaction for total phenolics and total flavonoids, indicating that the ability to synthesize phenolics differed across genotypes depending on their maturity stage. These factors pose a distinct opportunity for the use of genomic/phenomic selection to accurately predict and select genotypes that will accumulate and retain higher levels of nutritional quality traits. These predictions could take place through use of groundtruth data at multiple pre- and postharvest time points in a training set, and/or through the identification of pre- or at-harvest proxy traits that are useful predictors for postharvest nutrient levels. Monitoring of nutrient bioaccessibility (i.e., the levels available for absorption) is also important to ensure that improvements in crop nutritional quality are delivered to humans at time of consumption. Hayes et al. (2020) evaluated bioaccessible levels of chlorophylls and carotenoids in 71 spinach genotypes via simulated digestion in a shaking water bath. Significant differences were observed across genotypes for all traits except chlorophyll *b*, and within-trait correlations across seasons were stronger for β-carotene and lutein than for chlorophylls *a* and *b*.

Understanding the molecular processes that regulate nutrient stability and retention could also contribute to the development of biofortified crop varieties. For example, the esterification of carotenoids, particularly xanthophylls (i.e., oxygenated carotenoids), improves their stability against enzymatic and non-enzymatic factors, aiding in their sequestration and accumulation into fibrils or plastoglobules (Watkins et al. 2019; Mercadante et al. 2017). Morelli et al. (2024) observed that enhancing ß-carotene accumulation within plastids could be achieved by stimulating plastoglobule proliferation through intense light treatments, leading to significant increases in both the levels and bioaccessibility of ß-carotene in lettuce leaves.

In lettuce among other leafy greens, pigmentation can vary along the tip to base of the leaf (e.g., Lolla Rosa) or in a spotted pattern (e.g., Flashy Trout Back). While sampling full leaves would most closely represent the final product consumed by humans, subsampling an equal area (or mass) from the youngest fully expanded, representative leaves while avoiding veins and leaf margins could reduce variability across sample (and perhaps namely, market) types to enable assessment of nutrient concentrations. These concentrations could then be cross-analyzed with total marketable leaf area or total marketable biomass per plant or plot, allowing investigation of potential tradeoffs or synergies between these trait sets.

The extraction and characterization of phytochemicals depends on the choice of solvent, extraction phase, analytical standards, and the food/plant tissue matrix. Alongside environmental factors such as temperature and light that could impact phytochemical concentrations, evaluation of the solvent system’s extraction efficiency should be assessed not only with pigment standards but also with leaf samples. The polarity of pigment molecules significantly impacts their solubility and stability within solvent systems (Lictenthaler and Buschmann 2001). Analytical techniques for identifying individual carotenoid compounds in complex mixtures, such as leafy green extracts, have been limited. The challenge arises from spectral overlap and structural similarities among many pigment molecules, which obscures spectral deconvolution (Ashenafi et al. 2023).

Chlorophyll and carotenoid concentrations are typically measured on the basis of volume (of solution used to extract the compounds from leaves), fresh weight, dry weight, or leaf area. While dry weight is a reliable reference system (Lictenthaler and Buschmann 2001), pigment stability can be compromised under high-temperature conditions (often involved in drying) due to photodegradation and/or photoisomerization (Meléndez-Martínez et al. 2022). Fresh weight tends to be less reliable since it includes the water content in plant tissue, which can introduce more variability. For leafy greens such as lettuce and spinach, using leaf area as a reference system is also considered suitable (Lichtenthaler and Buschmann 2001).

In Table 1, we have prioritized nutrients that could feasibly be sensed based on their spectral or other chemical properties, and that have characterized causal relationships with other traits that already have market value. Thus, while quantification methods have been developed for essential nutrients such as B vitamins (ultra-performance liquid chromatography [UPLC]-selected reaction monitoring mass spectrometry [MS]; e.g., Marshall et al. 2024) and ascorbic acid (vitamin C; high-performance liquid chromatography [HPLC]) and for antinutrients such as phytate (relevant for mineral nutrient bioavailability in humans; HPLC or polyacrylamide gel electrophoresis; e.g., Raboy et al. 2020) and cyanogenic glucosides (ultra-HPLC triple quadrupole tandem MS; e.g., Zhong et al. 2020), those will not be discussed below due to lack of demonstrated sensing approaches.

**Table 1:** Overview of lab- and field-based methods for quantification of biochemical traits, with a focus on nutritional quality

Trait	Lab-based quantification of overall abundance	Lab-based quantification of individual compounds	Field-based quantification via sensing of overall abundance	Potential relationships with other traits having market value
Carotenoids, chlorophylls	Colorimetric assay using solvent-specific system of equations (Lichtenthaler 1987; Porra et al. 1989)	HPLC	RGB, multispectral fluorescence (Sytar et al. 2018)	Pigmentation, yield (via photosynthesis), provitamin A activity and other health benefits of certain carotenoids (Anitha et al. 2021; Bernstein and Arunkumar 2020)
Phenolics	Folin–Ciocalteu assay (colorimetric method: Ainsworth and Gillespie 2007)	HPLC	Multispectral fluorescence (Sytar et al. 2018)	Pigmentation, defense against biotic stresses, can promote and/or inhibit iron absorption; (Hart et al. 2017; Lesjak et al. 2019)
Macro- nutrients: protein, fat/oil, starch, fiber, moisture	AOAC methods (https://www.aoac.org/); NIRS; FT-NIRS & -MIRS (Madurapperumage et al. 2024); colorimetric assays (e.g., Osborne fractionation for protein solubility fractions; protein digestibility, Diatta-Holgate et al. 2023)	MS-based proteomics (Peters-Clarke et al. 2024), HPLC (protein composition; Sadeghi et al. 2023), LC–MS/MS (protein-bound amino acids: Yobi and Angelovici 2018; glycans: Couture et al. 2024), NMR (oils), GC–MS (fatty acid methyl esters)	Handheld/ portable NIRS (e.g., for dry matter, Hershberger et al. 2022)	Protein digestibility, protein/starch/lipid extraction yields (for use as ingredients), lack of rancidity, shelf life/storability
Mineral nutrients	NIRS, AOAC gravimetric loss method for ash (total mineral content)	ICP-MS, XRF	Portable XRF*, hyperspectral imaging (e.g., Ariza et al. 2024; Herzig et al. 2019)	Yield (agronomic biofortification)

high-performance liquid chromatography (HPLC), red–green–blue (RGB), near-infrared spectroscopy (NIRS), mid-infrared spectroscopy (MIRS), Fourier transform (FT), mass spectrometry (MS), liquid chromatography tandem mass spectrometry (LC–MS/MS), nuclear magnetic resonance (NMR) spectroscopy, gas chromatography-mass spectrometry (GC–MS), inductively coupled plasma-mass spectrometry (ICP-MS), X-ray fluorescence (XRF) spectroscopy. An asterisk signifies that a given technology is still in early testing

### Physiological traits: focus on water, nitrogen, and photosynthesis

Proximal sensing of plant physiological traits has been an active area of research for many years under both stress and optimal conditions due to the implicit understanding that these fundamental processes directly impact plant and crop performance (Bhardwaj et al. 2021; Gosa et al. 2019; van Bezouw et al. 2018). Among key physiological traits of interest, those involved in plant water and nitrogen status and light interception and photosynthesis have been especially targeted as they are key determinants of crop yields and relatedly, represent key subprocesses within plant growth and development.

Thermal and multispectral imaging systems along with hyperspectral, both spectroradiometers and imaging systems, can provide insight into the water status of plant canopies and their associated responses to drought stress. Infrared thermometry such as thermal imaging can be used to quantify canopy temperature, a functional trait that reflects transpiration as influenced by leaf stomata (Radin et al. 1994), and studies have demonstrated the usefulness of thermography when deployed on aerial platforms for detecting crop water stress and for site-specific management (Sepulcre-Cantó et al. 2006, 2005; Thorp et al. 2022). Further, several reports have demonstrated the utility of multispectral- and hyperspectral-based monitoring of crop water status. For example, tower-based hyperspectral remote sensing has been used to characterize leaf water potential and stomatal conductance across common bean genotypes (Wong et al. 2023a). Multispectral- and hyperspectral-based monitoring of crop water status relies on spectral indices that target either water content of the crop canopy directly (water indices) or structural changes to plant canopy (structural indices), or monitor the outcomes of low leaf water potential stress such as decreased photosynthesis/increased non photochemical quenching (xanthophyll indices) (Wong et al. 2022) or other alterations in plant metabolism (Melandri et al. 2021). Finally, structural changes in the crop canopy, such as loss of turgor, can be detected through alterations in reflective properties. This has been demonstrated in cotton through temporal sensing of NDVI across the day in which reflectance values changed as leaf wilting occurred (Pauli et al. 2016; Romano et al. 2011; Zhou et al. 2020).

Monitoring of crop nitrogen status also remains an important area of remote sensing research aimed at improving cropping system nitrogen use efficiency and mitigating environmental degradation. Classical approaches for estimating plant nitrogen status such as NDVI, Green Normalized Difference Vegetation Index (GNDVI), Normalized Difference Red-Edge Index (NDRE) rely on the relationship between plant nitrogen concentration and chlorophyll rather than targeting signatures originating from protein structures (which are the main pool of nitrogen in plants) (Magney et al. 2017). Newer deterministic approaches such as radiative transfer modeling of proteins are still in their early stages of development (Berger et al. 2020). Parametric regression (which includes VIs) and chemometrics are currently the most popular methods for estimating plant nitrogen content while ML and radiative transfer modeling are gaining popularity (Berger et al. 2020).

Characterizing and quantifying crop photosynthesis is another application of proximal sensing that has received significant attention given its direct relationship to plant productivity and yield (Runkle et al. 2025). Multispectral- and hyperspectral-based monitoring of crop photosynthetic activity and productivity can be estimated from spectral indices sensitive to pigment content and canopy structure (Pierrat et al. 2025). Leaf area index (LAI), a key structural trait closely linked to light interception and photosynthetic capacity, can be monitored using drone-based multispectral imagery, enabling high-throughput assessment of canopy dynamics under varying environmental conditions (Zhang et al. 2024). Additionally, solar-induced fluorescence (SIF), which requires ultra-spectral resolution (< 0.3 nm), serves as a physiological-based indicator of photosynthetic activity and productivity (Yang et al. 2021a; He et al. 2020; Fu et al. 2020). With improvements in technology and reductions in cost, these types of spectral imaging systems are being used more routinely in crop phenotyping and plant breeding, providing better insight into how environmental challenges (such as high-temperature and drought stress) can impact plant performance (Jiang et al. 2020; Kumagai et al. 2022; Pierrat et al. 2025).

However, while spectral data can be useful for characterizing plant physiological processes, analyses of these data can be challenging given the high dimensionality and collinearity between spectra (Wong 2023b; Magney 2025). To overcome these obstacles, ML techniques such as partial least squares regression, principal component analysis, random forest, support vector machines, and deep learning such as convolutional neural networks can be used with hyperspectral data (Guerri et al. 2024; Lu et al. 2020). These ML approaches reduce predictor redundancy and collinearity, extract informative features, and enable modeling of complex, nonlinear trait relationships. Here, spectral information sensitive to vegetation physiology, structure, and water is leveraged to maximize variation for predicting a suite of traits including photosynthesis, biochemistry, morphology, and water status across environmental conditions and treatments (Singh et al. 2015; Silva-Perez et al. 2018). While powerful, ML models require groundtruth data for calibration and validation and are generally limited to the local efforts, as there is potential for biases and increased error when extrapolating (Ji et al. 2024a).

## AI to identify novel resistance genes for improved disease resistance in crops

To date, the development of sensors to detect an early response of rice to pathogen infection is in nascent stages (Zheng et al. 2023; Tian et al. 2021). An alternative use of ML is to leverage knowledge of plant immune receptor-pathogen ligand interactions to engineer immune receptors that recognize a broader range of pathogens. Plant immune receptors, similar to animal innate immune receptors, bind and recognize specific pathogen-secreted molecules; therefore, we can direct the host immune system to respond to previously unrecognized pathogens by altering the binding properties of immune receptors. Immune receptors with novel ligand binding properties can be engineered through directed evolution (Rim et al. 2024, Fig. 2), a process in which high-throughput screening of lab-generated genetic variants identifies variants with a desired function. This process generates large datasets of genetic sequences and their evaluated trait, which can be leveraged in ML to map genotype–phenotype relationships and search for desired functions among natural variants.

**Fig. 2:**
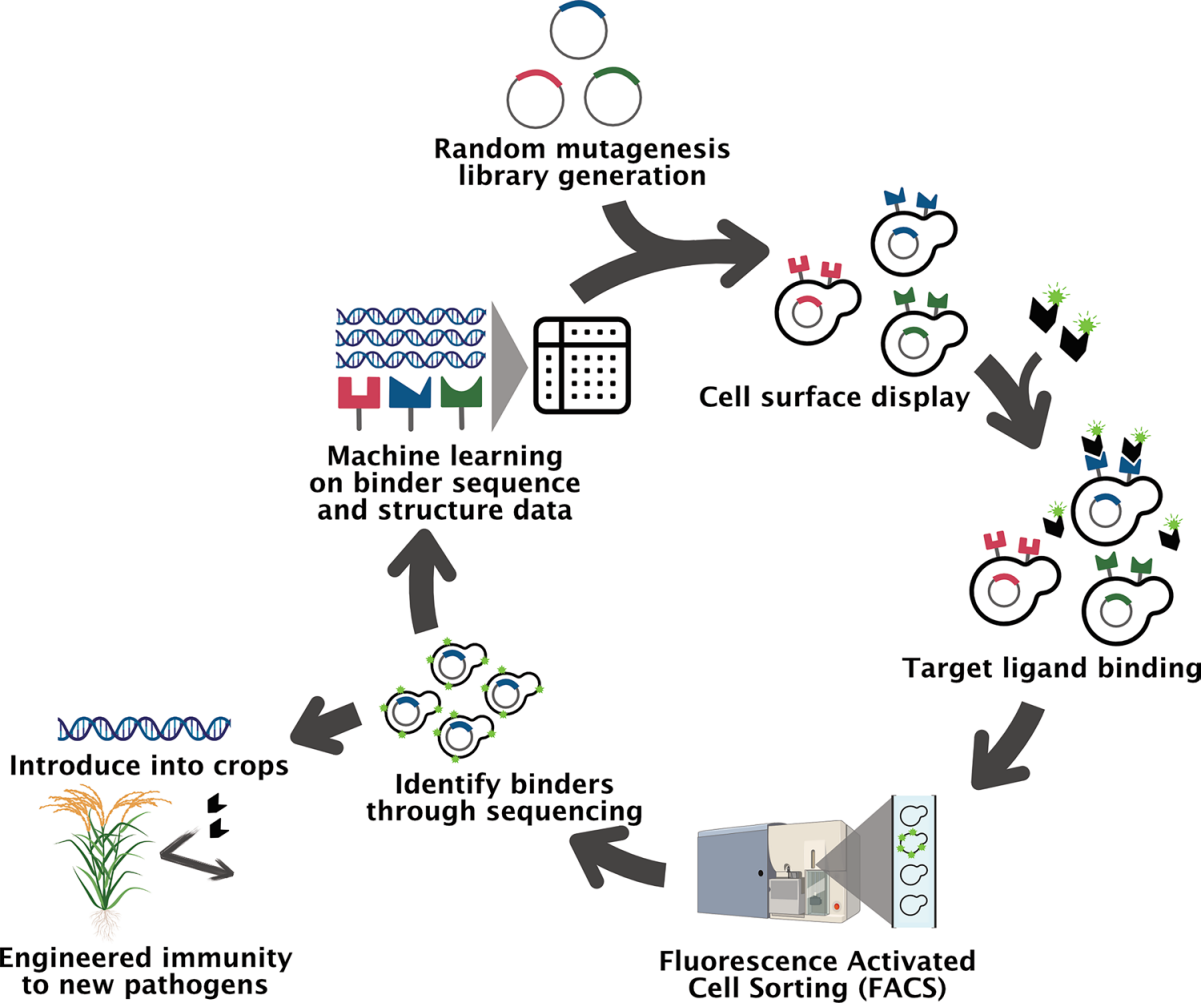
Schematic for applying AI/ML and directed evolution to plant immune receptor engineering

This strategy has been successfully applied to engineer receptors that bind a wide range of previously unrecognized ligands from *M. oryzae*, the causative agent for rice blast (Rim et al. 2024). Rim et al. expressed 2 × 10^7^ randomly mutagenized variants of the rice immune receptor Pik1 in a heterologous system, and selected variants that gained binding to a fluorescently labeled target ligand through fluorescence-activated cell sorting (FACS). Subsequently, Rim et al. searched for variants with high affinity binding to *M. oryzae* ligands that escape detection by all known natural receptor alleles through iterative FACS selection, with the goal of protecting against a wide range of *M. oryzae* strains expressing these different ligands. The starting library and receptor variants from each round of FACS selection were Illumina-sequenced. This sequencing data, therefore, captured the starting set of protein variants as well as variants that have gained a desired trait (target ligand binding) at each selection round.

Variant sequencing data were then used to train a protein language ML model and produce genotype-to-phenotype mappings for a database of 3,000 rice accessions (The 3,000 Rice Genomes Project 2014). Specifically, the model predicted which natural immune receptor variants would bind to different *M. oryzae* ligands. Doing so identified previously uncharacterized receptor variants with resistance to *M. oryzae* strains that could be used in crop breeding programs (Howard et al. 2025). This approach represents a novel strategy for identifying proteins with desired functions in large genomic databases.

## Expected trends in the integration of sensing-enabled crop modeling into molecular breeding efforts, for the improvement of crop yield and quality

### Sensing-enabled crop modeling

Phenotyping is often the primary bottleneck in modern plant breeding programs (Dipta et al. 2023). Plant breeding trials tend to involve large numbers of genetically related genotypes planted in close proximity, where subtle phenotypic differences can be difficult to detect. While manual phenotyping allows breeders to holistically assess traits in context and develop a deep familiarity with the crop system under study (Keller 1984), it is inherently limited by subjectivity and labor resources, and is typically associated with low temporal resolution. Sensing-enabled crop modeling offers a key solution to the phenotyping bottleneck by providing characterization methods for traits that cannot be measured at scale due to feasibility issues and can thereby provide novel outcomes that can be linked with the genotype and environment to support selection and prediction in breeding programs. Critically, crop models can mechanistically simulate priority traits by integrating data from granular, process-level phenotypes—such as canopy temperature, LAI, and growth rates—that are increasingly accessible at field and population scales, and with high temporal resolution, through modern sensing technologies.

Crop models use mass and energy transfer (Hank et al. 2015), soil productivity (Williams et al. 1989), and various environmental factors (Yu et al. 2023) to estimate crop growth and yield. Integrating proximal sensing data with these models can allow for model application at finer spatial resolution, shifting model predictions from field to plot scales (Oppelt 2010). Scaling down the region of interest of crop models enables examination of subtle phenotypic differences between the genotypes within breeding trials, or can allow for incorporation of sub-field-scale heterogeneity in model predictions. Sensing data can be assimilated into crop models by updating (adjusting model states based on new observations), forcing (inputting observed values to drive the model directly), and recalibration (adjusting model parameters to improve long-term accuracy) (Kasampalis et al. 2018; Smith et al. 2021). For example, remotely sensed irradiance, chlorophyll *a* and *b* content, and Rubisco content can support photosynthesis models, thereby improving the accuracy of plant growth and yield from crop models (Oppelt 2010). Parametrization of ‘cross-scale’ models that span the organ, plant, and crop scales (Wu 2023; Wu et al. 2022a; leaf and canopy scales, for instance) would be a ready application for proximal sensing (Peng et al. 2020). These cross-scale models could assist in achieving mechanistic understanding of adaptation to environmental stresses; for instance, by linking photosynthesis to yield (Wu et al. 2019, 2022a). Such models have also enabled comparative physiology as pertains to radiation use efficiency (Wu et al. 2024) and water limitation (van Oosterom et al. 2021). Peng et al. (2020) and Hammer et al. (2019) have called for cross-scale models that are biologically realistic (capturing essential mechanistic features) while also parsimonious and predictive, as venues in which to identify remaining gaps as focal points for further multidisciplinary and systems biology collaborations.

Sensing-enabled crop modeling can play valuable roles in breeding programs by providing more quantitative insights into the physiological underpinnings of performance within the TPE; for instance, optimal combinations of architectural traits and photosynthetic parameters given a set of environmental conditions. As examples, Chang et al. (2019) developed a 3-D canopy photosynthesis model in rice by integrating structural features with canopy gas exchange and weather data collected from a field trial with varying nitrogen treatments. Burgess et al. (2017) used 3-D canopy reconstructions and gas exchange measurements in a photosynthesis model to examine light and photosynthesis distributions in rice canopies, and Burgess et al. (2021) built upon this framework to evaluate these properties and plant architecture under varying nitrogen availability. Recent advances in AI-based 3-D reconstruction methods—such as Neural Radiance Field (NeRF) and Gaussian splatting—have further enhanced our ability to capture detailed plant architecture. For example, NeRF techniques have been applied to reconstruct the 3-D structure of rice plants() from smartphone images (Yang et al. 2024), offering breeders a rich resource of structural phenotypic information that can be linked with genotype for improved selection.

### Integration of sensing, crop modeling, and molecular breeding

**Fig. 3:**
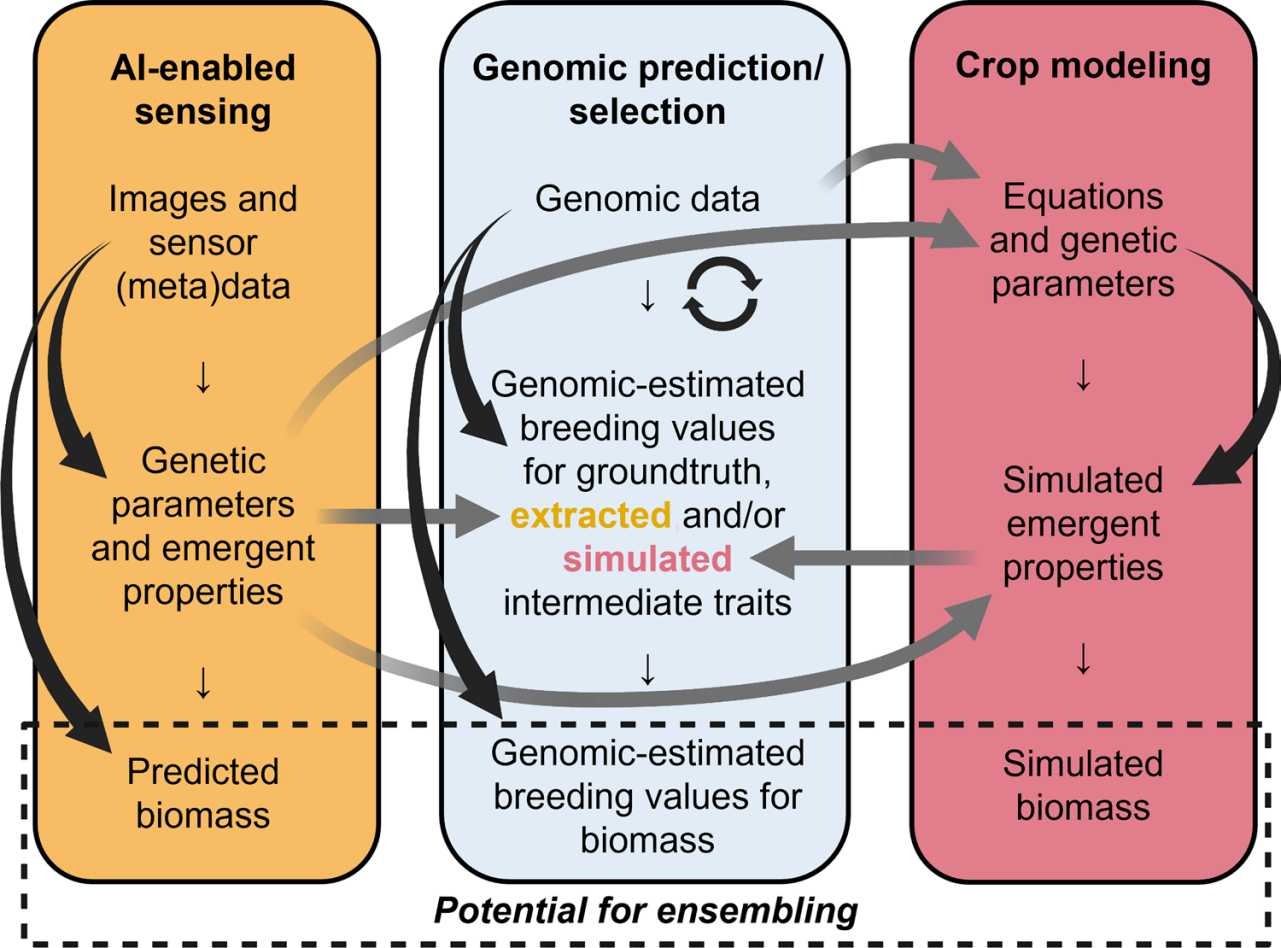
Schematic of opportunities to integrate AI-enabled sensing, genomic prediction/selection, and crop modeling workflows, using crop biomass as an example. The five long black arrows indicate use of AI, and the five gray arrows indicate an opportunity for direct data input between workflows

**Fig. 4:**
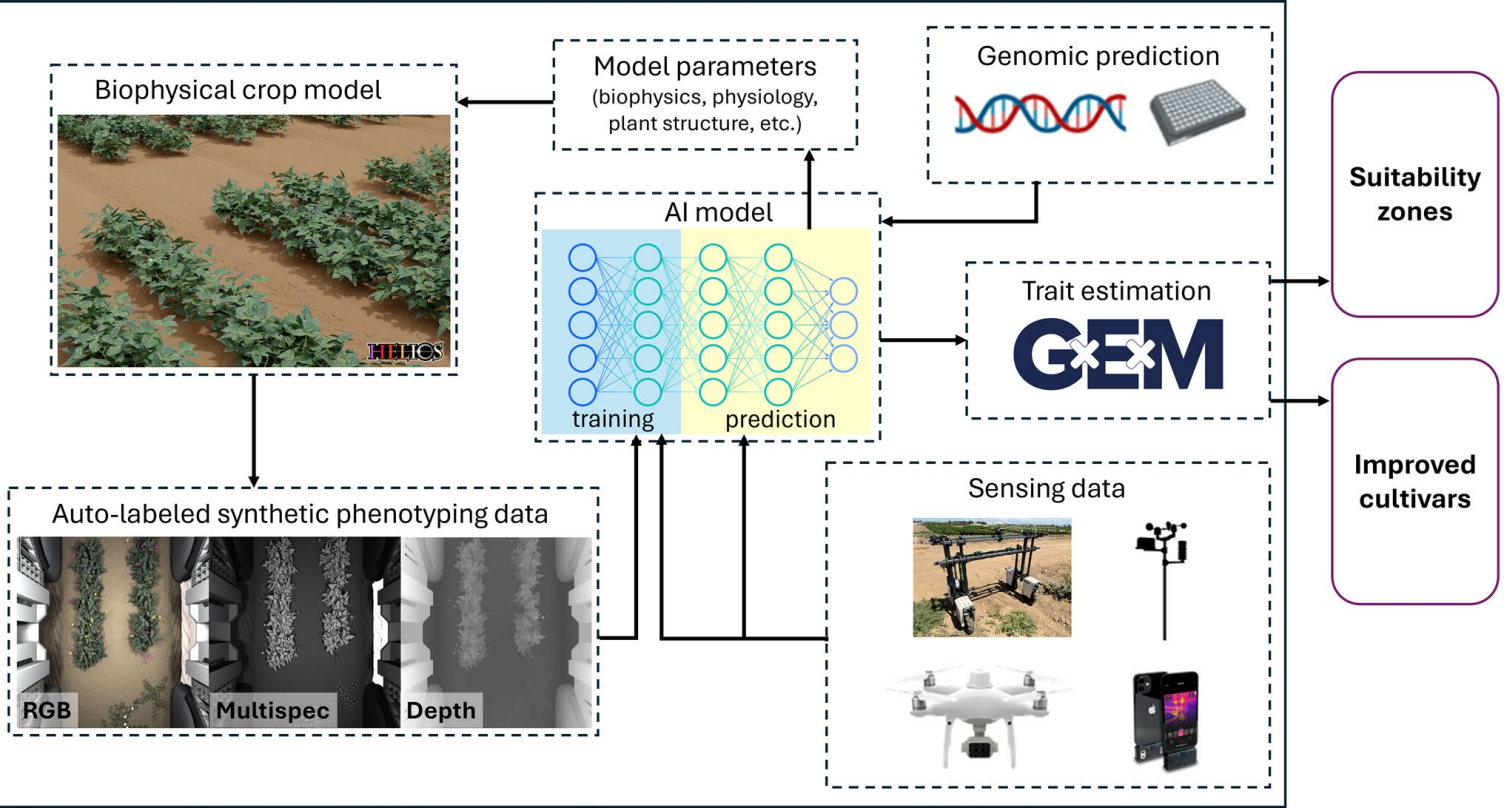
Schematic for integration of biophysical modeling, AI-enabled sensing, and breeding/genomics in multi-environment field trials, with applications in the identification of suitability zones (for crop species or cultivars thereof) and development of improved cultivars

Three-way integration of these domains would benefit from complementary and integratable, but also standalone, analytical frameworks to enable greater flexibility (e.g., based on availability of data, resources, and platforms) (Figs. 3 and 4). Sensing data and the plant, soil, and weather parameters needed for crop models are typically more difficult to collect with increasing distance from the home breeding station. However, when sensing is feasible via one or more platforms (or form factors), the resulting data can be integrated with genomic and crop modeling workstreams; for instance, one, two, or all three of these workstreams could be leveraged in predicting biomass in a given trial or (ideally) in a multi-environment trial (Fig. 3). Multi-environment testing in diverse environment types in the TPE is critical for maximizing on-farm genetic gains (Cooper et al. 2021, 2023; Lasdun et al. 2024). Three-way integration of these domains for optimized predictive ability in the TPE can be achieved through transitions between the real and synthetic domains via use of field-based sensing, AI-enabled feature extraction for parameterization of crop models, and generation of multiple modalities of auto-labeled synthetic data from those models, which are then used to train predictive models for application back to real field environments (Fig. 4). Genetic/genomic analyses can be integrated throughout that workflow both for mapping and prediction of extracted features and for analysis of relevant traits/features within genotypic/phenotypic classes (e.g., at key loci or for key market types) in both diverse and elite germplasm (Fig. 4). This three-way integrated framework could be used to establish quantitative TPEs, as a way to conduct envirotyping and establish suitability zones for use in germplasm evaluation and product placement. This framework can also be used in the development of improved cultivars through in-depth observation, modeling, and genomics-enabled dissection of the structural and functional properties of plant materials under evaluation in various stages of breeding.

Successful integration of sensing and crop modeling into molecular breeding strategies requires addressing shared operational, modeling, and communication challenges. These include logistical barriers to data collection, the generalizability and explainability of models, inconsistent terminology across disciplines, and the integration of data across platforms. In addition to logistical and modeling challenges, differences in terminology across domains can complicate interdisciplinary collaboration. For example, the term “spatial” may refer to image resolution in remote sensing and to autocorrelation structures in field trial analyses. Similarly, “traits” and “features” are used differently in genetics and computer vision contexts. Terms like “population”, “line”, “cultivar”, or “variety” also vary depending on crop reproductive biology and breeding system, with important implications for experimental design and data integration. Improved interdisciplinary communication and shared metadata standards, such as those supported by BrAPI, will be essential for resolving these terminological mismatches and supporting scalable, automated data pipelines.

Genetic parameters (Fig. 3) are considered to be only a property of G—tending to be highly heritable across environments in the germplasm of interest—and are good candidates to estimate on a genotype-specific basis in genomics-enabled crop models due to not necessarily needing to be measured in every environment. Emergent properties result from a more complete set of G, E, and M effects (Boote et al. 2021). Across the genotypes in a given field trial, genomic relationship matrices capture G (which allows estimation of genomic-estimated breeding values, i.e., the fraction of phenotypic variation that is heritable). In contrast, hyperspectral relationship matrices reflect G, E, and M such that models trained on them incorporate both heritable and non-heritable variation. Genomic prediction/selection models are based largely on G but can incorporate E and M statically (e.g., as covariates) or dynamically (e.g., via crop models) as surveyed above. Both the genetic parameters and emergent properties extracted from AI-enabled sensing and crop modeling can inform genomics-assisted methodologies and be leveraged directly in prediction and breeding. However, it is important that crop models have been appropriately parameterized for the set of genotypes under study (example in a multi-environment trial context: Rincent et al. 2017). It is also important that parameters known to vary in a given G, E, and/or M scenario are not treated as constants, which could give rise to artifacts between the main and interaction effects of G, E, and M on intermediate traits in the model and their nonlinear transformation into biomass accumulation and partitioning.

The number of plots on which validation of sensing- and modeling-derived traits (parameters and emergent properties) can feasibly be conducted varies widely by the trait. For example, pod count may be more feasible on a larger number of plots than total plant biomass; the latter is destructive and tends to be incompatible with standard machine harvest workflows. Validation strategies for these technologies would ideally be co-designed by the researchers conducting sensing, modeling, and breeding/genomics for maximized benefits to the integration of these workstreams (Interdisciplinary Plant Science Consortium 2023). For example, validation on fewer genotypes in more biological replicates (e.g., plots in the field) could be prioritized if needing to test repeatability on a by-genotype basis (Adak et al. 2023), whereas validation in more genotypes in fewer replicates could be prioritized if needing to maximize the range of the parameter space (within and across traits) that is surveyed. Models would also ideally be tested on elite genotypes rather than only diverse genotypes, so that predictive abilities are not inflated by nature of their development having been on genotypes with broad phenotypic ranges, and/or genotypes representing historical recombination as is often the case in diversity panels. In contrast, elite genotypes tend to represent recent recombination arising from crosses and the defined family structures that can be characteristic of breeding programs.

### Downstream applications of integrated sensing, modeling and breeding/genomics

Incorporation of elite genotypes into the groundtruth dataset for sensor validation and into the training set for predictive models would also benefit genomics and crop modeling efforts if these genotypes are being used as parents in the breeding program. This incorporation would allow the parents to be used as ‘tested genotypes’ in predictive models, and their breeding values (and selection accuracy) could be validated when evaluating their progeny in field settings. This integrated monitoring of parent-progeny relationships could inform the examination of mid-parent means, heterosis, transgressive segregation, additivity, dominance, pleiotropy, and epistasis. Predictive AI frameworks, particularly those that integrate sensing, crop modeling, and genomics, are increasingly well-suited to capture this complexity by deciphering relationships within and across genes and traits to inform prediction/selection strategies in genomics-enabled and physiological breeding (Voss-Fels et al. 2018).

With regards to genetic mapping, features derived from sensing and modeling could be subjected to genetic mapping as response variables (i.e., traits to be mapped). These features could also be helpful in further analyzing crop accessions that are in different genotypic classes (e.g., allelic states or haplotypes) at a given candidate locus for a given trait; e.g., to test whether those classes also systematically vary for another trait, as an indirect screen for pleiotropy (Boote et al. 2021) or confounds. For example, Zhou et al. (2021) found the measurement error between groundtruth and image-based phenotyping for five tassel traits in maize (and separately, for plant height in sorghum) was non-random and partially under genetic control. In maize, they found that groundtruth values for certain traits were correlated with measurement errors for other traits, and a largely distinct set of QTL were identified with the two phenotyping methods. They noted that increasing the number of genotypes or replicates would not have resolved this genetically determined measurement bias (related to open and closed tassel architecture, which is itself under genetic control). In partial contrast, Poland and Nelson (2011) found that most of the same QTL were identified across multiple human raters of ordinal severity of northern leaf blight in maize. In either case, interdisciplinary calibration on trait scoring schemas and checking for confounds will require careful thought in the experimental design and data collection and analysis phases as predictive models are developed, validated, and deployed in tested and untested G, E, and M scenarios.

Finally, the three-way integration of sensing, modeling, and breeding/genomics can enable detailed characterization of environment types in the TPE and of germplasm performance therein, in a manner that (particularly once integrating information from on-farm trials) informs both breeding and agronomy (Cooper et al. 2021). Agronomic applications could include product placement (e.g., which variety to grow and on which field within a farm) and optimized management of that variety in that environment (Cooper et al. 2020). Imperfect correlations between on-station and on-farm yields could reduce selection accuracy for on-farm yield, if breeders are selecting largely based on data collected at experiment stations in certain breeding stages (Werner et al. 2025; Cooper et al. 2023; Vandemark et al. 2017). Using methods such as gap analysis, which incorporates crop modeling, could further quantitate and dissect these gaps in a manner that informs decision making as germplasm is selected both by breeders and by growers (van Ittersum et al. 2013; Cooper et al. 2020; Hajjarpoor et al. 2021).

In summary, integration of modeling, sensing, and breeding/genomics enables capturing—and leveraging in prediction—the 3-D structure and function of the crop and G and repeatable GxE effects therein. Such integrated breeding frameworks could be pivotal in achieving genetic gains to close yield and quality gaps (Cooper et al. 2020; van Ittersum et al. 2013; Hawkes et al. 2012). Continually optimizing these frameworks for maximized genetic gain on-farm will be an ongoing challenge that would benefit from 1) meticulous and standardized data management platforms and practices (for sensing, modeling, groundtruth, and other breeding program data and metadata) and 2) regular collection of ancillary data streams in breeding field trials to enable crop modeling (Washburn et al. 2020), such as soil tests, detailed management records that include irrigation and fertilization timings and amounts, and on-site weather data that includes solar radiation. Given the current balance in cost vs. realized utility of sensors (e.g., for plant height and early vigor), programs already using sensing may be seeking to extract additional traits, or programs that are not yet using sensing may benefit from a shared working model in which a common platform and operator are available for occasional use across multiple programs. Either way, the question arises: if we were to routinely have detailed (and persistent, in a digital sense) records regarding structural and functional traits in a set of genotypes, would we know what breeding decisions to make, and how would our retrospective analyses of genetic gain be benefitted? Ultimately we would still be selecting upon breeding values for ‘total’ agronomic performance and quality (e.g., in a selection index), the weights in our index would indicate prioritization, and prioritization (and which groundtruth and proxy traits are in the index) would be driven in part based on our understanding of factors underlying crop performance in the TPE (Gosa et al. 2019; Boote et al. 2021; Cooper et al. 2023). As such, continuous validation and feedback between single- and multi-trait predictive models and field outcomes (Boote et al. 2021) and appropriate use of training and test sets will be critical as integrated crop simulation/prediction systems are deployed, to avoid circularity and ensure that hypotheses are being generated that breeders and researchers in adjacent disciplines (such as plant physiology and biophysics) can test empirically in relevant germplasm.

While quality traits are still not explicitly valorized in many crop types, crop quality is inherent to the concept of ‘marketable’ yield. Continued identification and leveraging in breeding of win–win scenarios for crop quality and productivity would be helpful, alongside cost-effective monitoring of quality traits in mid- to advanced-stage material (e.g., in case such material could be used as a parent in recurrent selection) rather than only in a relatively small number of final varietal candidates. Looking ahead, the fusion of sensing and modeling into molecular breeding strategies represents not just a technical revolution but a paradigm shift in how we view crop improvement. Rather than relying solely on a few to several groundtruthed agronomic and quality traits, breeders will have the decision support tools to dissect in more detail the effects of G, E, and M on a fuller range of priority traits and extract accurate and unbiased genetic values therefrom, for maximized response to selection. Such tools will help bridge the gap between research and real-world agricultural challenges, ensuring that future crop varieties are climate-resilient in both their productivity and quality profiles for improved food, nutritional, and economic security in current and future generations.

## Abbreviations

AI: Artificial intelligence
APSIM: Agricultural production systems sIMulator
BrAPI: Breeding API
CHM: Canopy height model
CGM: Crop growth model
CRISPR-Cas: Clustered regularly interspaced short palindromic repeats-CRISPR associated protein
DSSAT: Decision support system for agrotechnology transfer
EC: Environmental covariates
ExG: Excess green vegetation index
FACS: Fluorescence-activated cell sorting
GAN: Generative adversarial network
G-BLUP: Genomic best linear unbiased prediction
GP: Genomic prediction
GxExM: Genotype × environment × management interactions
GCMs: Global circulation models
GDD: Growing degree day
LAI: Leaf area index
LiDAR: Light detection and ranging
LMM: Linear mixed-effect models
ML: Machine learning
MSE: Managed stress environment
NeRF: Neural radiance field
NDVI: Normalized difference vegetation index
QTL: Quantitative trait loci
RCPs: Representative concentration pathways
RI: Recombinant inbred
RF: Rate of development toward time to flowering
SAR: Synthetic aperture radar
SIF: Solar-induced fluorescence
TPE: Target population of environments
VIs: Vegetation indices
WL: Water-limited
WGP: Whole-genome prediction

## References

[CR1] Adak A, Murray SC, Anderson SL (2023) Temporal phenomic predictions from unoccupied aerial systems can outperform genomic predictions. G3 Genes Genomes Genet 13(1):jkac294. 10.1093/g3journal/jkac294 10.1093/g3journal/jkac294 PMC9836347 36445027

[CR2] Ahmad S, Ahmad A, Ali H, Hussain A, Garcia Y Garcia A, Khan MA, Zia-Ul-Haq M, Hasanuzzaman M, Hoogenboom G (2013) Application of the CSM-CERES-Rice model for evaluation of plant density and irrigation management of transplanted rice for an irrigated semiarid environment. Irrig Sci 31(3):491–506. 10.1007/s00271-012-0324-6

[CR3] Ainsworth EA, Gillespie KM (2007) Estimation of total phenolic content and other oxidation substrates in plant tissues using Folin-Ciocalteu reagent. Nat Protoc. 10.1038/nprot.2007.102 17446889 10.1038/nprot.2007.102

[CR4] Allen MT, Prusinkiewicz P, DeJong TM (2005) Using L-systems for modeling source–sink interactions, architecture and physiology of growing trees: The L-PEACH model. New Phytol 166(3):869–880. 10.1111/j.1469-8137.2005.01348.x 15869648 10.1111/j.1469-8137.2005.01348.x

[CR5] An G, Yu C, Yan C, Wang M, Zhang W, Jia Y, Shi C, Larkin RM, Chen J, Lavelle D, Michelmore RW, Kuang H (2022) Loss-of-function of SAWTOOTH 1 affects leaf dorsiventrality genes to promote leafy heads in lettuce. Plant Cell 34(11):4329–4347. 10.1093/plcell/koac234 35916734 10.1093/plcell/koac234 PMC9614500

[CR6] Anacleto R, Cuevas RP, Jimenez R, Llorente C, Nissila E, Henry R, Sreenivasulu N (2015) Prospects of breeding high-quality rice using post-genomic tools. Theor Appl Genet 128(8):1449–1466. 10.1007/s00122-015-2537-6 25993897 10.1007/s00122-015-2537-6

[CR7] Angel Y, Shiklomanov AN (2022) Remote detection and monitoring of plant traits: theory and practice. In: Annual Plant Reviews online (pp. 313–344). John Wiley & Sons, Ltd. 10.1002/9781119312994.apr0778

[CR8] Anitha RE, Janani R, Peethambaran D, Baskaran V (2021) Lactucaxanthin protects retinal pigment epithelium from hyperglycemia-regulated hypoxia/ER stress/VEGF pathway mediated angiogenesis in ARPE-19 cell and rat model. Eur J Pharmacol 899:174014. 10.1016/j.ejphar.2021.174014 33705802 10.1016/j.ejphar.2021.174014

[CR9] Antolin LAS, Heinemann AB, Marin FR (2021) Impact assessment of common bean availability in Brazil under climate change scenarios. Agric Syst 191:103174. 10.1016/j.agsy.2021.103174

[CR10] Araus JL, Cairns JE (2014) Field high-throughput phenotyping: The new crop breeding frontier. Trends Plant Sci 19(1):52–61. 10.1016/j.tplants.2013.09.008 24139902 10.1016/j.tplants.2013.09.008

[CR11] Araus JL, Kefauver SC, Zaman-Allah M, Olsen MS, Cairns JE (2018) Translating high-throughput phenotyping into genetic gain. Trends Plant Sci 23(5):451–466. 10.1016/j.tplants.2018.02.001 29555431 10.1016/j.tplants.2018.02.001 PMC5931794

[CR12] Araus JL, Kefauver SC, Vergara-Díaz O, Gracia-Romero A, Rezzouk FZ, Segarra J, Buchaillot ML, Chang-Espino M, Vatter T, Sanchez-Bragado R, Fernandez-Gallego JA, Serret MD, Bort J (2021) Crop phenotyping in a context of global change: what to measure and how to do it. J Integr Plant Biol 64(2):592–618. 10.1111/jipb.13191 10.1111/jipb.13191 34807514

[CR13] Ariza AA, Sotta N, Fujiwara T, Guo W, Kamiya T (2024) A multi-target regression method to predict element concentrations in tomato leaves using hyperspectral imaging. Plant Phenomics 6:0146. 10.34133/plantphenomics.0146 38629079 10.34133/plantphenomics.0146 PMC11020135

[CR14] Arriola-Valverde S, Villagra-Mendoza K, Méndez-Morales M, Solórzano-Quintana M, Gómez-Calderón N, Rimolo-Donadio R (2020) Analysis of crop dynamics through close-range UAS photogrammetry. IEEE Int Symp Circuit Syst (ISCAS). 10.1109/ISCAS45731.2020.9181285

[CR15] Arrouays D, Grundy MG, Hartemink AE, Hempel JW, Heuvelink GBM, Hong SY, Lagacherie P, Lelyk G, McBratney AB, McKenzie NJ, Mendonca-Santos MDL, Minasny B, Montanarella L, Odeh IOA, Sanchez PA, Thompson JA, Zhang G-L (2014) GlobalSoilMap: Toward a fine-resolution global grid of soil properties. In: Sparks DL (ed) Advances in agronomy.cambridge, pp 93–134. 10.1016/B978-0-12-800137-0.00003-0

[CR16] Ashenafi EL, Nyman MC, Shelley JT, Mattson NS (2023) Spectral properties and stability of selected carotenoid and chlorophyll compounds in different solvent systems. Food Chem Adv 2:100178. 10.1016/j.focha.2022.100178

[CR17] Assefa T, Assibi Mahama A, Brown AV, Cannon EKS, Rubyogo JC, Rao IM, Blair MW, Cannon SB (2019) A review of breeding objectives, genomic resources, and marker-assisted methods in common bean (*Phaseolus vulgaris* L.). Mol Breed 39(2):20. 10.1007/s11032-018-0920-0

[CR18] Badowiec A, Weidner S (2014) Proteomic changes in the roots of germinating *Phaseolus vulgaris* seeds in response to chilling stress and post-stress recovery. J Plant Physiol 171(6):389–398. 10.1016/j.jplph.2013.10.020 24594390 10.1016/j.jplph.2013.10.020

[CR19] Bai G, Ge Y, Scoby D, Leavitt B, Stoerger V, Kirchgessner N, Irmak S, Graef G, Schnable J, Awada T (2019) NU-Spidercam: a large-scale, cable-driven, integrated sensing and robotic system for advanced phenotyping, remote sensing, and agronomic research. Comput Electron Agric 160:71–81. 10.1016/j.compag.2019.03.009

[CR20] Bailey BN (2019) Helios: a scalable 3D plant and environmental biophysical modeling framework. Front Plant Sci. 10.3389/fpls.2019.01185 31681349 10.3389/fpls.2019.01185 PMC6813926

[CR21] Barten TJ, Kosola KR, Dohleman FG, Eller M, Brzostowski L, Mueller S, Mioduszewski J, Gu C, Kashyap S, Ralston L, Renaud A, Hall M, Mack D, Gillespie K (2022) Short-stature maize reduced wind damage during the 2020 midwestern derecho, improving yields and greenhouse gas outcomes. Crop Sci 62(6):2439–2450. 10.1002/csc2.20823

[CR22] Batjes NH, Calisto L, de Sousa LM (2024) Providing quality-assessed and standardised soil data to support global mapping and modelling (WoSIS snapshot 2023). Earth Syst Sci Data 16(10):4735–4765. 10.5194/essd-16-4735-2024

[CR23] Baxter I (2020) We aren’t good at picking candidate genes, and it’s slowing us down. Curr Opin Plant Biol 54:57–60. 10.1016/j.pbi.2020.01.006 32106014 10.1016/j.pbi.2020.01.006

[CR24] Beaver JS, de Jensen CE, Miklas PN, Porch TG (2020) Contributions in Puerto Rico to Bean, Phaseolus spp., Research. 104(1)

[CR25] Bellucci E, Bitocchi E, Ferrarini A, Benazzo A, Biagetti E, Klie S, Minio A, Rau D, Rodriguez M, Panziera A, Venturini L, Attene G, Albertini E, Jackson SA, Nanni L, Fernie AR, Nikoloski Z, Bertorelle G, Delledonne M, Papa R (2014) Decreased nucleotide and expression diversity and modified coexpression patterns characterize domestication in the common bean. Plant Cell 26(5):1901–1912. 10.1105/tpc.114.124040 24850850 10.1105/tpc.114.124040 PMC4079357

[CR26] Berger K, Verrelst J, Féret J-B, Wang Z, Wocher M, Strathmann M, Danner M, Mauser W, Hank T (2020) Crop nitrogen monitoring: Recent progress and principal developments in the context of imaging spectroscopy missions. Remote Sens Environ 242:111758. 10.1016/j.rse.2020.111758 36082364 10.1016/j.rse.2020.111758 PMC7613361

[CR27] Bernardo R (2016) Bandwagons i, too, have known. Theor Appl Genet 129(12):2323–2332. 10.1007/s00122-016-2772-5 27681088 10.1007/s00122-016-2772-5

[CR28] Bernstein PS, Arunkumar R (2020) The emerging roles of the macular pigment carotenoids throughout the lifespan and in prenatal supplementation. J Lipid Res 62:100038. 10.1194/jlr.TR120000956 10.1194/jlr.TR120000956 PMC7933486 32709621

[CR29] Bertier LD, Ron M, Huo H, Bradford KJ, Britt AB, Michelmore RW (2018) High-resolution analysis of the efficiency, heritability, and editing outcomes of CRISPR/Cas9-induced modifications of *NCED4* in lettuce (*Lactuca sativa*). G3 Genes|genomes|genetics 8(5):1513–1521. 10.1534/g3.117.300396 29511025 10.1534/g3.117.300396 PMC5940144

[CR30] Bhardwaj A, Devi P, Chaudhary S, Rani A, Jha UC, Kumar S, Bindumadhava H, Prasad PVV, Sharma KD, Siddique KHM, Nayyar H (2021) ‘Omics’ approaches in developing combined drought and heat tolerance in food crops. Plant Cell Rep 41(3):699–739. 10.1007/s00299-021-02742-0 34223931 10.1007/s00299-021-02742-0

[CR31] Bhattarai G, Shi A, Feng C, Dhillon B, Mou B, Correll JC (2020) Genome wide association studies in multiple spinach breeding populations refine downy mildew race 13 resistance genes. Front Plant Sci 11:563187. 10.3389/fpls.2020.563187 33193490 10.3389/fpls.2020.563187 PMC7609621

[CR32] Bhattarai G, Shi A, Mou B, Correll JC (2022) Resequencing worldwide spinach germplasm for identification of field resistance QTLs to downy mildew and assessment of genomic selection methods. Hortic Res 9:uhac205. 10.1093/hr/uhac205 36467269 10.1093/hr/uhac205 PMC9715576

[CR33] Bin Rahman ANMR, Zhang J (2023) Trends in rice research: 2030 and beyond. Food Energy Secur 12(2):e390. 10.1002/fes3.390

[CR34] Blackburn GA (2007) Hyperspectral remote sensing of plant pigments. J Exp Bot 58(4):855–867. 10.1093/jxb/erl123 16990372 10.1093/jxb/erl123

[CR35] Boon CS, McClements DJ, Weiss J, Decker EA (2010) Factors influencing the chemical stability of carotenoids in foods. Crit Rev Food Sci Nutr. 10.1080/10408390802565889 20544442 10.1080/10408390802565889

[CR36] Boote KJ, Prasad V, Allen LH, Singh P, Jones JW (2018) Modeling sensitivity of grain yield to elevated temperature in the DSSAT crop models for peanut, soybean, dry bean, chickpea, sorghum, and millet. Eur J Agron 100:99–109. 10.1016/j.eja.2017.09.002

[CR37] Boote KJ, Jones JW, Hoogenboom G (2021) Incorporating realistic trait physiology into crop growth models to support genetic improvement. In Silico Plants 3(1):diab002. 10.1093/insilicoplants/diab002

[CR38] Borrill P (2019) Blurring the boundaries between cereal crops and model plants. New Phytol 228(6):1721–1727. 10.1111/nph.16229 31571228 10.1111/nph.16229

[CR39] Boyer JS, Byrne P, Cassman KG, Cooper M, Delmer D, Greene T, Gruis F, Habben J, Hausmann N, Kenny N, Lafitte R, Paszkiewicz S, Porter D, Schlegel A, Schussler J, Setter T, Shanahan J, Sharp RE, Vyn TJ, Warner D, Gaffney J (2013) The U.S. drought of 2012 in perspective: a call to action. Glob Food Secur 2(3):139–143. 10.1016/j.gfs.2013.08.002

[CR40] Broughton WJ, Hernández G, Blair M, Beebe S, Gepts P, Vanderleyden J (2003) Beans (*Phaseolus* spp.) – model food legumes. Plant Soil 252(1):55–128. 10.1023/A:1024146710611

[CR41] Bunning ML, Kendall PA, Stone MB, Stonaker FH, Stushnoff C (2010) Effects of seasonal variation on sensory properties and total phenolic content of 5 lettuce cultivars. J Food Sci 75(3):S156–S161. 10.1111/j.1750-3841.2010.01533.x 20492312 10.1111/j.1750-3841.2010.01533.x

[CR42] Buresh RJ, Singh U, Godwin DC, Ritchie JT, De Datta SK (1991) Simulating soil nitrogen transformations and crop response to nitrogen using the CERES-RICE model. IRRI research paper series-International Rice Research Institute

[CR43] Burgess AJ, Retkute R, Herman T, Murchie EH (2017) Exploring relationships between canopy architecture, light distribution, and photosynthesis in contrasting rice genotypes using 3D canopy reconstruction. Front Plant Sci 8:734. 10.3389/fpls.2017.00734 28567045 10.3389/fpls.2017.00734 PMC5434157

[CR44] Burgess AJ, Durand M, Gibbs JA, Retkute R, Robson TM, Murchie EH (2021) The effect of canopy architecture on the patterning of “windflecks” within a wheat canopy. Plant Cell Environ 44(11):3524–3537. 10.1111/pce.14168 34418115 10.1111/pce.14168

[CR45] Bustos-Korts D, Malosetti M, Chapman S, van Eeuwijk F (2016) Modelling of genotype by environment interaction and prediction of complex traits across multiple environments as a synthesis of crop growth modelling, genetics and statistics. In: Yin X, Struik PC (eds) Crop systems biology: narrowing the gaps between crop modelling and genetics. pp 55–82. 10.1007/978-3-319-20562-5_3

[CR46] Buxbaum N, Lieth JH, Earles M (2022) Non-destructive plant biomass monitoring with high spatio-temporal resolution via proximal RGB-d imagery and end-to-end deep learning. Front Plant Sci 13:758818 35498682 10.3389/fpls.2022.758818 PMC9043900

[CR47] Cai X, Sun X, Xu C, Sun H, Wang X, Ge C, Zhang Z, Wang Q, Fei Z, Jiao C, Wang Q (2021) Genomic analyses provide insights into spinach domestication and the genetic basis of agronomic traits. Nat Commun 12(1):7246. 10.1038/s41467-021-27432-z 34903739 10.1038/s41467-021-27432-z PMC8668906

[CR48] Calisto L, de Sousa LM, Batjes NH (2023) Standardised soil profile data for the world (WoSIS snapshot – December 2023), 10.17027/isric-wdcsoils-20231130

[CR49] Campbell MT, Knecht AC, Berger B, Brien CJ, Wang D, Walia H (2015) Integrating image-based phenomics and association analysis to dissect the genetic architecture of temporal salinity responses in rice. Plant Physiol 168(4):1476–1489. 10.1104/pp.15.00450 26111541 10.1104/pp.15.00450 PMC4528749

[CR50] Castaneda-Saucedo MC, Córdova-Téllez L, Tapia-Campos E, Delgado-Alvarado A, González-Hernández VA, Santacruz-Varela A, Loza-Tavera H, García-de-los-Santos G, Vargas-Suárez M (2014) Dehydrins patterns in common bean exposed to drought and watered conditions. Rev Fitotec Mex 37(1):59–68

[CR51] Cattivelli L, Rizza F, Badeck F-W, Mazzucotelli E, Mastrangelo AM, Francia E, Marè C, Tondelli A, Stanca AM (2008) Drought tolerance improvement in crop plants: an integrated view from breeding to genomics. Field Crop Res 105(1–2):1–14. 10.1016/j.fcr.2007.07.004

[CR52] Cawse-Nicholson K, Townsend PA, Schimel D, Assiri AM, Blake PL, Buongiorno MF, Campbell P, Carmon N, Casey KA, Correa-Pabón RE, Dahlin KM, Dashti H, Dennison PE, Dierssen H, Erickson A, Fisher JB, Frouin R, Gatebe CK, Gholizadeh H, Gierach M, Glenn NF, Goodman JA, Griffith DM, Guild L, Hakkenberg CR, Hochberg EJ, Holmes TRH, Hu C, Hulley G, Huemmrich KF, Kudela RM, Kokaly RF, Lee CM, Martin R, Miller CE, Moses WJ, Muller-Karger FE, Ortiz JD, Otis DB, Pahlevan N, Painter TH, Pavlick R, Poulter B, Qi Yi, Realmuto VJ, Roberts D, Schaepman ME, Schneider FD, Schwandner FM, Serbin SP, Shiklomanov AN, Stavros EN, Thompson DR, Torres-Perez JL, Turpie KR, Tzortziou M, Ustin S, Yu Q, Yusup Y, Zhang Q (2021) NASA’s surface biology and geology designated observable: a perspective on surface imaging algorithms. Remote Sens Environ 257:112349. 10.1016/j.rse.2021.112349

[CR53] Chang T-G, Zhao H, Wang N, Song Q-F, Xiao Y, Qu M, Zhu X-G (2019) A three-dimensional canopy photosynthesis model in rice with a complete description of the canopy architecture, leaf physiology, and mechanical properties. J Exp Bot 70(9):2479–2490. 10.1093/jxb/ery430 30801123 10.1093/jxb/ery430 PMC6487591

[CR54] Chen D, Neumann K, Friedel S, Kilian B, Chen M, Altmann T, Klukas C (2014) Dissecting the phenotypic components of crop plant growth and drought responses based on high-throughput image analysis. Plant Cell 26(12):4636–4655. 10.1105/tpc.114.129601 25501589 10.1105/tpc.114.129601 PMC4311194

[CR55] Chiaravallotti I, Lin J, Arief V, Jahufer Z, Osorno JM, McClean P, Jarquin D, Hoyos-Villegas V (2024) Simulations of multiple breeding strategy scenarios in common bean for assessing genomic selection accuracy and model updating. Plant Genome 17(1):e20388. 10.1002/tpg2.20388 38317595 10.1002/tpg2.20388 PMC12806982

[CR56] Clark RT, MacCurdy RB, Jung JK, Shaff JE, McCouch SR, Aneshansley DJ, Kochian LV (2011) Three-dimensional root phenotyping with a novel imaging and software platform. Plant Physiol 156(2):455–465. 10.1104/pp.110.169102 21454799 10.1104/pp.110.169102 PMC3177249

[CR57] Cobb JN, DeClerck G, Greenberg A, Clark R, McCouch S (2013) Next-generation phenotyping: requirements and strategies for enhancing our understanding of genotype–phenotype relationships and its relevance to crop improvement. Theor Appl Genet 126(4):867–887. 10.1007/s00122-013-2066-0 23471459 10.1007/s00122-013-2066-0 PMC3607725

[CR58] Coelho AP, de Faria RT, Lemos LB, Cazuza Neto A (2023) Application of the CSM-CROPGRO-Dry bean model to optimize irrigation as a function of sowing date in common bean cultivars. Field Crop Res 293:108840. 10.1016/j.fcr.2023.108840

[CR59] Collins K, Zhao K, Jiao C, Xu C, Cai X, Wang X, Ge C, Dai S, Wang Q, Wang Q, Fei Z, Zheng Y (2019) SpinachBase: a central portal for spinach genomics. Database 2019:baz072. 10.1093/database/baz072 31211398 10.1093/database/baz072 PMC6580994

[CR60] Cooper M, Gho C, Leafgren R, Tang T, Messina C (2014) Breeding drought-tolerant maize hybrids for the US corn-belt: discovery to product. J Exp Bot 65(21):6191–6204. 10.1093/jxb/eru064 24596174 10.1093/jxb/eru064

[CR61] Cooper M, Technow F, Messina C, Gho C, Totir LR (2016) Use of crop growth models with whole-genome prediction: application to a maize multienvironment trial. Crop Sci 56(5):2141–2156. 10.2135/cropsci2015.08.0512

[CR62] Cooper M, Tang T, Gho C, Hart T, Hammer G, Messina C (2020) Integrating genetic gain and gap analysis to predict improvements in crop productivity. Crop Sci 60(2):582–604. 10.1002/csc2.20109

[CR63] Cooper M, Voss-Fels KP, Messina CD, Tang T, Hammer GL (2021) Tackling G × E × M interactions to close on-farm yield-gaps: creating novel pathways for crop improvement by predicting contributions of genetics and management to crop productivity. Theor Appl Genet 134(6):1625–1644. 10.1007/s00122-021-03812-3 33738512 10.1007/s00122-021-03812-3 PMC8206060

[CR64] Cooper M, Powell O, Gho C, Tang T, Messina C (2023) Extending the breeder’s equation to take aim at the target population of environments. Front Plant Sci 14:1129591. 10.3389/fpls.2023.1129591 36895882 10.3389/fpls.2023.1129591 PMC9990092

[CR65] Cortinovis G, Frascarelli G, Di Vittori V, Papa R (2020) Current state and perspectives in population genomics of the common bean. Plants. 10.3390/plants9030330 32150958 10.3390/plants9030330 PMC7154925

[CR66] Cortinovis G, Vincenzi L, Anderson R, Marturano G, Marsh JI, Bayer PE, Rocchetti L, Frascarelli G, Lanzavecchia G, Pieri A, Benazzo A, Bellucci E, Di Vittori V, Nanni L, Ferreira Fernández JJ, Rossato M, Aguilar OM, Morrell PL, Rodriguez M, Gioia T, Neumann K, Alvarez Diaz JC, Gratias A, Klopp C, Bitocchi E, Geffroy V, Delledonne M, Edwards D, Papa R (2024) Adaptive gene loss in the common bean pan-genome during range expansion and domestication. Nat Commun 15(1):6698. 10.1038/s41467-024-51032-2 39107305 10.1038/s41467-024-51032-2 PMC11303546

[CR67] Costa-Neto GMF, Morais Júnior OP, Heinemann AB, de Castro AP, Duarte JB (2020) A novel GIS-based tool to reveal spatial trends in reaction norm: upland rice case study. Euphytica 216(3):37. 10.1007/s10681-020-2573-4

[CR68] Costa-Neto G, Crossa J, Fritsche-Neto R (2021a) Enviromic assembly increases accuracy and reduces costs of the genomic prediction for yield plasticity in maize. Front Plant Sci. 10.3389/fpls.2021.717552 34691099 10.3389/fpls.2021.717552 PMC8529011

[CR69] Costa-Neto G, Fritsche-Neto R, Crossa J (2021b) Nonlinear kernels, dominance, and envirotyping data increase the accuracy of genome-based prediction in multi-environment trials. Heredity 126(1):92–106. 10.1038/s41437-020-00353-1 32855544 10.1038/s41437-020-00353-1 PMC7852533

[CR70] Costa-Neto G, Crespo-Herrera L, Fradgley N, Gardner K, Bentley AR, Dreisigacker S, Fritsche-Neto R, Montesinos-López OA, Crossa J (2023) Envirome-wide associations enhance multi-year genome-based prediction of historical wheat breeding data. G3 Genes Genomes Genetics 13(2):jkac313. 10.1093/g3journal/jkac313 36454213 10.1093/g3journal/jkac313 PMC9911085

[CR71] Couture G, Cheang SE, Suarez C, Chen Y, Bacalzo NP, Jiang J, Weng C-YC, Stacy A, Castillo JJ, Delannoy-Bruno O, Webber DM, Barratt MJ, Gordon JI, Mills DA, German JB, Fukagawa NK, Lebrilla CB (2024) A multi-glycomic platform for the analysis of food carbohydrates. Nat Protoc. 10.1038/s41596-024-01017-8 39026121 10.1038/s41596-024-01017-8

[CR72] Crain J, Mondal S, Rutkoski J, Singh RP, Poland J (2018) Combining high-throughput phenotyping and genomic information to increase prediction and selection accuracy in wheat breeding. Plant Genome 11(1):170043. 10.3835/plantgenome2017.05.0043 10.3835/plantgenome2017.05.0043 PMC12962554 29505641

[CR73] Crossa J, Fritsche-Neto R, Montesinos-Lopez OA, Costa-Neto G, Dreisigacker S, Montesinos-Lopez A, Bentley AR (2021) The modern plant breeding triangle: optimizing the use of genomics, phenomics, and enviromics data. Front Plant Sci. 10.3389/fpls.2021.651480 33936136 10.3389/fpls.2021.651480 PMC8085545

[CR74] Crossa J, Montesinos-López OA, Pérez-Rodríguez P, Costa-Neto G, Fritsche-Neto R, Ortiz R, Martini JWR, Lillemo M, Montesinos-López A, Jarquin D, Breseghello F, Cuevas J, Rincent R (2022) Genome and environment based prediction models and methods of complex traits incorporating genotype × environment interaction. In: Ahmadi N, Bartholomé J (eds) Genomic prediction of complex traits: methods and protocols. Springer, New York, pp 245–283. 10.1007/978-1-0716-2205-6_9

[CR75] Crossa J, Cerón-Rojas JJ, Montesinos-López A, Montesinos-López OA, Punzalan J, Famoso A, Fritsche-Neto R (2025) Evaluating the effectiveness of selection indices and their genomic prediction using environmental and historical rice data. G3 Genes Genomes Genetics. 10.1093/g3journal/jkaf087 40238949 10.1093/g3journal/jkaf087 PMC12135014

[CR76] Cui X-Z, Feng Q, Wang S-Z, Zhang J-H (2022) Monocular depth estimation with self-supervised learning for vineyard unmanned agricultural vehicle. Sensors. 10.3390/s22030721 35161463 10.3390/s22030721 PMC8838921

[CR77] Dale R, Oswald S, Jalihal A, LaPorte M-F, Fletcher DM, Hubbard A, Shiu S-H, Nelson ADL, Bucksch A (2021) Overcoming the challenges to enhancing experimental plant biology with computational modeling. Front Plant Sci. 10.3389/fpls.2021.687652 34354723 10.3389/fpls.2021.687652 PMC8329482

[CR78] Damerum A, Selmes SL, Biggi GF, Clarkson GJ, Rothwell SD, Truco MJ, Michelmore RW, Hancock RD, Shellcock C, Chapman MA, Taylor G (2015) Elucidating the genetic basis of antioxidant status in lettuce (*Lactuca sativa*). Hortic Res 2(1):15055. 10.1038/hortres.2015.55 26640696 10.1038/hortres.2015.55 PMC4660231

[CR79] Dash S, Campbell JD, Cannon EKS, Cleary AM, Huang W, Kalberer SR, Karingula V, Rice AG, Singh J, Umale PE, Weeks NT, Wilkey AP, Farmer AD, Cannon SB (2016) Legume information system (LegumeInfo.org): A key component of a set of federated data resources for the legume family. Nucleic Acids Research 44(D1):D1181-1188. 10.1093/nar/gkv1159 26546515 10.1093/nar/gkv1159 PMC4702835

[CR80] Davis WV, Lucier G (2021) Vegetables and Pulses Outlook: November 2021

[CR81] Dhillon AK, Sharma N, Dosanjh NK, Goyal M, Mahajan G (2018) Variation in the nutritional quality of rice straw and grain in response to different nitrogen levels. J Plant Nutr 41(15):1946–1956. 10.1080/01904167.2018.1482915

[CR82] Dias MG, Camões MFGFC, Oliveira L (2014) Carotenoid stability in fruits, vegetables and working standards – effect of storage temperature and time. Food Chem 156:37–41. 10.1016/j.foodchem.2014.01.050 24629935 10.1016/j.foodchem.2014.01.050

[CR83] Diatta-Holgate E, Anderson JS, Hatch R, Tuinstra MR, Weil C (2023) Rapid determination of protein digestibility in sorghum before and after cooking. MethodsX 10:102162. 10.1016/j.mex.2023.102162 37091954 10.1016/j.mex.2023.102162 PMC10119968

[CR84] Diepenbrock CH, Gore MA (2015) Closing the divide between human nutrition and plant breeding. Crop Sci 55(4):1437–1448. 10.2135/cropsci2014.08.0555

[CR85] Diepenbrock CH, Tang T, Jines M, Technow F, Lira S, Podlich D, Cooper M, Messina C (2021) Can we harness digital technologies and physiology to hasten genetic gain in US maize breeding? Plant Physiol 188(2):1141–1157. 10.1093/plphys/kiab527 10.1093/plphys/kiab527 PMC8825268 34791474

[CR86] Dipta B, Sood S, Devi R, Bhardwaj V, Mangal V, Thakur AK, Kumar V, Pandey NK, Rathore A, Singh AK (2023) Digitalization of potato breeding program: improving data collection and management. Heliyon 9(1):e12974. 10.1016/j.heliyon.2023.e12974 36747944 10.1016/j.heliyon.2023.e12974 PMC9898647

[CR87] Doorenbos J, Kassam AH, Bentvelsen C, Uittenbogaard G (1979) Yield Response to Water. In Irrigation and Agricultural Development (pp. 257–280). Elsevier. https://linkinghub.elsevier.com/retrieve/pii/B9780080256757500212

[CR88] Ebert AW (2014) Potential of underutilized traditional vegetables and legume crops to contribute to food and nutritional security, income and more sustainable production systems. Sustainability. 10.3390/su6010319

[CR89] Elad Y, Pertot I (2014) Climate change impacts on plant pathogens and plant diseases. J Crop Improv 28(1):99–139. 10.1080/15427528.2014.865412

[CR90] El-Naggar AG, Jolly B, Hedley CB, Horne D, Roudier P, Clothier BE (2021) The use of terrestrial LiDAR to monitor crop growth and account for within-field variability of crop coefficients and water use. Comput Electron Agric 190:106416. 10.1016/j.compag.2021.106416

[CR91] Enaru B, Drețcanu G, Pop TD, Stǎnilǎ A, Diaconeasa Z (2021) Anthocyanins: factors affecting their stability and degradation. Antioxidants. 10.3390/antiox10121967 34943070 10.3390/antiox10121967 PMC8750456

[CR92] Espe MB, Yang H, Cassman KG, Guilpart N, Sharifi H, Linquist BA (2016) Estimating yield potential in temperate high-yielding, direct-seeded US rice production systems. Field Crops Res 193:123–132. 10.1016/j.fcr.2016.04.003

[CR93] Fanzo J, Davis C, McLaren R, Choufani J (2018) The effect of climate change across food systems: implications for nutrition outcomes. Glob Food Secur 18:12–19. 10.1016/j.gfs.2018.06.001

[CR94] FAO. (2023). World Food and Agriculture – Statistical Yearbook 2023. FAO; https://openknowledge.fao.org/handle/20.500.14283/cc8166en

[CR95] FAOSTAT. (2024). Retrieved May 16, 2025, from https://www.fao.org/faostat/en/#home

[CR96] Farooq MA, Gao S, Hassan MA, Huang Z, Rasheed A, Hearne S, Prasanna B, Li X, Li H (2024) Artificial intelligence in plant breeding. Trends Genet. 10.1016/j.tig.2024.07.001 39117482 10.1016/j.tig.2024.07.001

[CR97] Feng C, Bluhm B, Shi A, Correll JC (2018) Development of molecular markers linked to three spinach downy mildew resistance loci. Euphytica 214(10):174. 10.1007/s10681-018-2258-4

[CR98] Fernandez J, Orth K (2018) Rise of a cereal killer: the biology of *Magnaporthe oryzae* biotrophic growth. Trends Microbiol 26(7):582–597 29395728 10.1016/j.tim.2017.12.007 PMC6003838

[CR99] Fernie AR, Tadmor Y, Zamir D (2006) Natural genetic variation for improving crop quality. Curr Opin Plant Biol 9(2):196–202. 10.1016/j.pbi.2006.01.010 16480915 10.1016/j.pbi.2006.01.010

[CR100] Ferrari S, Cunha MLO, do Valle Polycarpo G, Zied DC, de Oliveira LCA, Júnior EF (2022) Genotypic variation in grain nutritional content and agronomic traits of upland rice: Strategy to reduce hunger and malnutrition. Cereal Res Commun 50(4):1155–1163. 10.1007/s42976-022-00257-2

[CR101] Ferrero-Serrano Á, Cantos C, Assmann SM (2019) The role of dwarfing traits in historical and modern agriculture with a focus on rice. Cold Spring Harb Perspect Biol 11(11):a034645. 10.1101/cshperspect.a034645 31358515 10.1101/cshperspect.a034645 PMC6824242

[CR103] Fischer G, Nachtergaele FO, van Velthuizen HT, Chiozza F, Franceschini G, Henry M, Muchoney D, Tramberend S (2021) Global agro-ecological zones v4 – model documentation. FAO, Rome

[CR104] Fletcher K, Shin O-H, Clark KJ, Feng C, Putman AI, Correll JC, Klosterman SJ, Van Deynze A, Michelmore RW (2022) Ancestral chromosomes for family Peronosporaceae inferred from a telomere-to-telomere genome assembly of *Peronospora effusa*. Mol Plant-Microbe Interactions® 35(6):450–463. 10.1094/MPMI-09-21-0227-R 10.1094/MPMI-09-21-0227-R 35226812

[CR105] Fu P, Meacham-Hensold K, Siebers MH, Bernacchi CJ (2020) The inverse relationship between solar-induced fluorescence yield and photosynthetic capacity: benefits for field phenotyping. J Exp Bot 72(4):1295–1306 10.1093/jxb/eraa537 PMC7904154 33340310

[CR106] Furbank RT, Tester M (2011) Phenomics – technologies to relieve the phenotyping bottleneck. Trends Plant Sci 16(12):635–644. 10.1016/j.tplants.2011.09.005 22074787 10.1016/j.tplants.2011.09.005

[CR107] Gaffney J, Schussler J, Löffler C, Cai W, Paszkiewicz S, Messina C, Groeteke J, Keaschall J, Cooper M (2015) Industry-scale evaluation of maize hybrids selected for increased yield in drought-stress conditions of the US corn belt. Crop Sci 55(4):1608–1618. 10.2135/cropsci2014.09.0654

[CR108] Gamon JA (2015) Reviews and syntheses: optical sampling of the flux tower footprint. Biogeosciences 12(14):4509–4523. 10.5194/bg-12-4509-2015

[CR109] Gano B, Bhadra S, Vilbig JM, Ahmed N, Sagan V, Shakoor N (2024) Drone-based imaging sensors, techniques, and applications in plant phenotyping for crop breeding: a comprehensive review. Plant Phenome J 7(1):e20100. 10.1002/ppj2.20100

[CR110] Garcia T, Duitama J, Zullo SS, Gil J, Ariani A, Dohle S, Palkovic A, Skeen P, Bermudez-Santana CI, Debouck DG, Martínez-Castillo J, Gepts P, Chacón-Sánchez MI (2021) Comprehensive genomic resources related to domestication and crop improvement traits in Lima bean. Nat Commun 12(1):702. 10.1038/s41467-021-20921-1 33514713 10.1038/s41467-021-20921-1 PMC7846787

[CR111] Gauch J (1992) Statistical analysis of regional yield trials: AMMI analysis of factorial designs. https://www.cabidigitallibrary.org/doi/full/10.5555/19931643324

[CR112] Gepts P, Aragão FJL, Barros ED, Blair MW, Brondani R, Broughton W, Galasso I, Hernández G, Kami J, Lariguet P, McClean P, Melotto M, Miklas P, Pauls P, Pedrosa-Harand A, Porch T, Sánchez F, Sparvoli F, Yu K (2008) Genomics of phaseolus beans, a major source of dietary protein and micronutrients in the tropics. In: Moore PH, Ming R (eds) Genomics of tropical crop plants. Springer, New York, pp 113–143. 10.1007/978-0-387-71219-2_5

[CR113] Gevartosky R, Carvalho HF, Costa-Neto G, Montesinos-López OA, Crossa J, Fritsche-Neto R (2023) Enviromic-based kernels may optimize resource allocation with multi-trait multi-environment genomic prediction for tropical Maize. BMC Plant Biol 23(1):10. 10.1186/s12870-022-03975-1 36604618 10.1186/s12870-022-03975-1 PMC9814176

[CR114] Gomez D, Selvaraj MG, Casas J, Mathiyazhagan K, Rodriguez M, Assefa T, Mlaki A, Nyakunga G, Kato F, Mukankusi C, Girma E, Mosquera G, Arredondo V, Espitia E (2024) Advancing common bean (*Phaseolus vulgaris* L.) disease detection with YOLO driven deep learning to enhance agricultural AI. Sci Rep 14(1):15596. 10.1038/s41598-024-66281-w 38971939 10.1038/s41598-024-66281-w PMC11227504

[CR115] Goodstein DM, Shu S, Howson R, Neupane R, Hayes RD, Fazo J, Mitros T, Dirks W, Hellsten U, Putnam N, Rokhsar DS (2012) Phytozome: a comparative platform for green plant genomics. Nucleic Acids Res 40(D1):D1178–D1186. 10.1093/nar/gkr944 22110026 10.1093/nar/gkr944 PMC3245001

[CR116] Gosa SC, Lupo Y, Moshelion M (2019) Quantitative and comparative analysis of whole-plant performance for functional physiological traits phenotyping: new tools to support pre-breeding and plant stress physiology studies. Plant Sci 282:49–59. 10.1016/j.plantsci.2018.05.008 31003611 10.1016/j.plantsci.2018.05.008

[CR117] Green RO, Mahowald N, Ung C, Thompson DR, Bator L, Bennet M, Bernas M, Blackway N, Bradley C, Cha J, Clark P, Clark R, Cloud D, Diaz E, Ben Dor E, Duren R, Eastwood M, Ehlmann BL, Fuentes L, Zan J (2020) The Earth Surface Mineral Dust Source Investigation: An Earth Science Imaging Spectroscopy Mission. 2020 IEEE Aerospace Conference, 1–15. 10.1109/AERO47225.2020.9172731

[CR118] Großkinsky DK, Syaifullah SJ, Roitsch T (2018) Integration of multi-omics techniques and physiological phenotyping within a holistic phenomics approach to study senescence in model and crop plants. J Exp Bot 69(4):825–844. 10.1093/jxb/erx333 29444308 10.1093/jxb/erx333

[CR119] Guerri MF, Distante C, Spagnolo P, Bougourzi F, Taleb-Ahmed A (2024) Deep learning techniques for hyperspectral image analysis in agriculture: a review. ISPRS Open J Photogrammetry Remote Sens 12:100062. 10.1016/j.ophoto.2024.100062

[CR120] Hajjarpoor A, Kholová J, Pasupuleti J, Soltani A, Burridge J, Degala SB, Gattu S, Murali TV, Garin V, Radhakrishnan T, Vadez V (2021) Environmental characterization and yield gap analysis to tackle genotype-by-environment-by-management interactions and map region-specific agronomic and breeding targets in groundnut. Field Crop Res 267:108160. 10.1016/j.fcr.2021.108160

[CR121] Hammer G, Messina C, Wu A, Cooper M (2019) Biological reality and parsimony in crop models—why we need both in crop improvement! In Silico Plants 1(1):diz010. 10.1093/insilicoplants/diz010

[CR122] Han R, Truco MJ, Lavelle DO, Michelmore RW (2021) A composite analysis of flowering time regulation in lettuce. Front Plant Sci. 10.3389/fpls.2021.632708 33763095 10.3389/fpls.2021.632708 PMC7982828

[CR123] Hank TB, Bach H, Mauser W (2015) Using a remote sensing-supported hydro-agroecological model for field-scale simulation of heterogeneous crop growth and yield: application for wheat in Central Europe. Remote Sens. 10.3390/rs70403934

[CR124] Harrison MT, Tardieu F, Dong Z, Messina CD, Hammer GL (2014) Characterizing drought stress and trait influence on maize yield under current and future conditions. Glob Change Biol 20(3):867–878. 10.1111/gcb.12381 10.1111/gcb.12381 24038882

[CR125] Hart JJ, Tako E, Glahn RP (2017) Characterization of polyphenol effects on inhibition and promotion of iron uptake by Caco-2 cells. J Agric Food Chem. 10.1021/acs.jafc.6b05755 28361541 10.1021/acs.jafc.6b05755

[CR126] Hawkes C, Turner R, Ferguson E, Johnston D, Shankar B, Waage J, Haseen F, Homans H, Hussein J, Marais D, McNeil G (2012) Current and planned research on agriculture for improved nutrition: A mapping and a gap analysis. Leverhulme Centre for Integrative Research on Agriculture and Health

[CR127] Hayes M, Pottorff M, Kay C, Van Deynze A, Osorio-Marin J, Lila MA, Iorrizo M, Ferruzzi MG (2020) In vitro bioaccessibility of carotenoids and chlorophylls in a diverse collection of spinach accessions and commercial cultivars. J Agric Food Chem. 10.1021/acs.jafc.0c00158 32125838 10.1021/acs.jafc.0c00158

[CR128] He L, Magney T, Dutta D, Yin Y, Köhler P, Grossmann K, Stutz J, Dold C, Hatfield J, Guan K, Peng B, Frankenberg C (2020) From the ground to space: using solar-induced chlorophyll fluorescence to estimate crop productivity. Geophys Res Lett 47(7):e2020GL087474. 10.1029/2020GL087474

[CR129] Heinemann AB, Ramirez-Villegas J, Souza TLPO, Didonet AD, di Stefano JG, Boote KJ, Jarvis A (2016) Drought impact on rainfed common bean production areas in Brazil. Agric for Meteorol 225:57–74. 10.1016/j.agrformet.2016.05.010

[CR130] Heinemann AB, Costa-Neto G, Fritsche-Neto R, da Matta DH, Fernandes IK (2022) Enviromic prediction is useful to define the limits of climate adaptation: a case study of common bean in Brazil. Field Crops Res 286:108628. 10.1016/j.fcr.2022.108628

[CR131] Hengl T, Jesus JMde, Heuvelink GBM, Gonzalez MR, Kilibarda M, Blagotić A, Shangguan W, Wright MN, Geng X, Bauer-Marschallinger B, Guevara MA, Vargas R, MacMillan RA, Batjes NH, Leenaars JGB, Ribeiro E, Wheeler I, Mantel S, Kempen B (2017) SoilGrids250m: Global gridded soil information based on machine learning. PLoS ONE 12(2):e0169748. 10.1371/journal.pone.0169748 28207752 10.1371/journal.pone.0169748 PMC5313206

[CR132] Hershberger J, Mbanjo EGN, Peteti P, Ikpan A, Ogunpaimo K, Nafiu K, Rabbi IY, Gore MA (2022) Low-cost, handheld near-infrared spectroscopy for root dry matter content prediction in cassava. Plant Phenome J 5(1):e20040. 10.1002/ppj2.20040

[CR133] Herzig P, Backhaus A, Seiffert U, von Wirén N, Pillen K, Maurer A (2019) Genetic dissection of grain elements predicted by hyperspectral imaging associated with yield-related traits in a wild barley NAM population. Plant Sci 285:151–164. 10.1016/j.plantsci.2019.05.008 31203880 10.1016/j.plantsci.2019.05.008

[CR134] Heslot N, Akdemir D, Sorrells ME, Jannink J-L (2014) Integrating environmental covariates and crop modeling into the genomic selection framework to predict genotype by environment interactions. Theor Appl Genet 127(2):463–480. 10.1007/s00122-013-2231-5 24264761 10.1007/s00122-013-2231-5

[CR135] Hirakawa H, Toyoda A, Itoh T, Suzuki Y, Nagano AJ, Sugiyama S, Onodera Y (2021) A spinach genome assembly with remarkable completeness, and its use for rapid identification of candidate genes for agronomic traits. DNA Res 28(3):dsab004. 10.1093/dnares/dsab004 34142133 10.1093/dnares/dsab004 PMC8231376

[CR136] Hoffmann H, Zhao G, Asseng S, Bindi M, Biernath C, Constantin J, Coucheney E, Dechow R, Doro L, Eckersten H, Gaiser T, Grosz B, Heinlein F, Kassie BT, Kersebaum K-C, Klein C, Kuhnert M, Lewan E, Moriondo M, Nendel C, Priesack E, Raynal H, Roggero PP, Rötter RP, Siebert S, Specka X, Tao F, Teixeira E, Trombi G, Wallach D, Weihermüller L, Yeluripati J, Ewert F (2016) Impact of spatial soil and climate input data aggregation on regional yield simulations. PLoS ONE 11(4):e0151782. 10.1371/journal.pone.0151782 27055028 10.1371/journal.pone.0151782 PMC4824533

[CR137] Holzworth DP, Huth NI, deVoil PG, Zurcher EJ, Herrmann NI, McLean G, Chenu K, van Oosterom EJ, Snow V, Murphy C, Moore AD, Brown H, Whish JPM, Verrall S, Fainges J, Bell LW, Peake AS, Poulton PL, Hochman Z, Thorburn PJ, Gaydon DS, Dalgliesh NP, Rodriguez D, Cox H, Chapman S, Doherty A, Teixeira E, Sharp J, Cichota R, Vogeler I, Li FY, Wang E, Hammer GL, Robertson MJ, Dimes JP, Whitbread AM, Hunt J, van Rees H, McClelland T, Carberry PS, Hargreaves JNG, MacLeod N, McDonald C, Harsdorf J, Wedgwood S, Keating BA (2014) Apsim – evolution towards a new generation of agricultural systems simulation. Environ Model Softw 62:327–350. 10.1016/j.envsoft.2014.07.009

[CR138] Homolová L, Malenovský Z, Clevers JGPW, García-Santos G, Schaepman ME (2013) Review of optical-based remote sensing for plant trait mapping. Ecol Complex 15:1–16. 10.1016/j.ecocom.2013.06.003

[CR139] Hoogenboom G, Porter CH, Boote KJ, Shelia V, Wilkens PW, Singh U, White JW, Asseng S, Lizaso JI, Moreno LP, Pavan W, Ogoshi R, Hunt LA, Tsuji GY, Jones JW (2019) The DSSAT Crop Modeling Ecosystem. https://hub.ifdc.org/handle/20.500.14297/2873

[CR140] Horie T, Nakagawa HN, Centeno HGS, Kropff MJ (1995) The rice simulation model SIMRIW and its testing. In Modeling the impact of climate change on rice production in Asia (pp. 95–139). https://research.wur.nl/en/publications/the-rice-simulation-model-simriw-and-its-testing

[CR141] Howard LR, Pandjaitan N, Morelock T, Gil MI (2002) Antioxidant capacity and phenolic content of spinach as affected by genetics and growing season. J Agric Food Chem 50(21):5891–5896. 10.1021/jf020507o 12358455 10.1021/jf020507o

[CR142] Howard AJ, Rim EY, Garrett OD, Shim Y, Notwell JH, Ronald PC (2025). Combining Directed Evolution with Machine Learning Enables Accurate Genotype-to-henotype Predictions (p. 2025.01.27.635131). bioRxiv. 10.1101/2025.01.27.635131

[CR143] Hu T, Zhang X, Khanal S, Wilson R, Leng G, Toman EM, Wang X, Li Y, Zhao K (2024) Climate change impacts on crop yields: a review of empirical findings, statistical crop models, and machine learning methods. Environ Model Softw 179:106119. 10.1016/j.envsoft.2024.106119

[CR144] Hu H, Rincent R, Runcie DE (2025a) MegaLMM improves genomic predictions in new environments using environmental covariates. Genetics 229(1):iyae171. 10.1093/genetics/iyae171 39471330 10.1093/genetics/iyae171 PMC11708919

[CR145] Hu H, Yuan X, Saini DK, Yang T, Wu X, Wu R, Liu Z, Jan F, Mir RR, Liu L, Miao J, Liu N, Xu P (2025b) A panomics-driven framework for the improvement of major food legume crops: advances, challenges, and future prospects. Hortic Res 12(7):uhaf091. 10.1093/hr/uhaf091 40352287 10.1093/hr/uhaf091 PMC12064956

[CR146] Huang J, Tang S, Ousama A-I, Wang R (2002) Rice yield estimation using remote sensing and simulation model. J Zhejiang Univ-SCIENCE A 3(4):461–466. 10.1631/BF02839491

[CR147] Hulse-Kemp AM, Bostan H, Chen S, Ashrafi H, Stoffel K, Sanseverino W, Li L, Cheng S, Schatz MC, Garvin T, du Toit LJ, Tseng E, Chin J, Iorizzo M, Van Deynze A (2021) An anchored chromosome-scale genome assembly of spinach improves annotation and reveals extensive gene rearrangements in euasterids. Plant Genome 14(2):e20101. 10.1002/tpg2.20101 34109759 10.1002/tpg2.20101 PMC12806983

[CR148] Hummel M, Hallahan BF, Brychkova G, Ramirez-Villegas J, Guwela V, Chataika B, Curley E, McKeown PC, Morrison L, Talsma EF, Beebe S, Jarvis A, Chirwa R, Spillane C (2018) Reduction in nutritional quality and growing area suitability of common bean under climate change induced drought stress in Africa. Sci Rep 8(1):16187. 10.1038/s41598-018-33952-4 30385766 10.1038/s41598-018-33952-4 PMC6212502

[CR149] Hussain T, Anothai J, Nualsri C, Ata-Ul-Karim ST, Duangpan S, Hussain N, Ali A (2023) Assessment of CSM–CERES–Rice as a decision support tool in the identification of high-yielding drought-tolerant upland rice genotypes. Agronomy 13(2):432. 10.3390/agronomy13020432

[CR150] Hwang C, Correll MJ, Gezan SA, Zhang L, Bhakta MS, Vallejos CE, Boote KJ, Clavijo-Michelangeli JA, Jones JW (2017) Next generation crop models: a modular approach to model early vegetative and reproductive development of the common bean (*Phaseolus vulgaris* L). Agric Syst 155:225–239. 10.1016/j.agsy.2016.10.010 28701815 10.1016/j.agsy.2016.10.010 PMC5485674

[CR151] FAO & IIASA. 2023. Harmonized World Soil Database version 2.0. Rome and Laxenburg. 10.4060/cc3823en

[CR152] Interdisciplinary Plant Science Consortium I (2023) Inclusive collaboration across plant physiology and genomics: Now is the time! Plant Direct, 7(5): e493. 10.1002/pld3.493 10.1002/pld3.493 PMC10192722 37214275

[CR153] Jain R, Jenkins J, Shu S, Chern M, Martin JA, Copetti D, Duong PQ, Pham NT, Kudrna DA, Talag J, Schackwitz WS, Lipzen AM, Dilworth D, Bauer D, Grimwood J, Nelson CR, Xing F, Xie W, Barry KW, Wing RA, Schmutz J, Li G, Ronald PC (2019) Genome sequence of the model rice variety KitaakeX. BMC Genomics 20(1):905. 10.1186/s12864-019-6262-4 31775618 10.1186/s12864-019-6262-4 PMC6882167

[CR154] Jamshidi S, Murgia T, Morales-Ona AG, Cerioli T, Famoso AN, Cammarano D, Wang DR (2024) Modeling interactions of planting date and phenology in Louisiana rice under current and future climate conditions. Crop Sci 64(4):2274–2287. 10.1002/csc2.21036

[CR155] Jarquín D, Crossa J, Lacaze X, Du Cheyron P, Daucourt J, Lorgeou J, Piraux F, Guerreiro L, Pérez P, Calus M, Burgueño J, de los Campos G (2014) A reaction norm model for genomic selection using high-dimensional genomic and environmental data. Theor Appl Genet 127(3):595–607. 10.1007/s00122-013-2243-1 24337101 10.1007/s00122-013-2243-1 PMC3931944

[CR156] Jarquin D, de Leon N, Romay C, Bohn M, Buckler ES, Ciampitti I, Edwards J, Ertl D, Flint-Garcia S, Gore MA, Graham C, Hirsch CN, Holland JB, Hooker D, Kaeppler SM, Knoll J, Lee EC, Lawrence-Dill CJ, Lynch JP, Moose SP, Murray SC, Nelson R, Rocheford T, Schnable JC, Schnable PS, Smith M, Springer N, Thomison P, Tuinstra M, Wisser RJ, Xu W, Yu J, Lorenz A (2021) Utility of climatic information via combining ability models to improve genomic prediction for yield within the Genomes to Fields Maize Project. Front Genet. 10.3389/fgene.2020.592769 33763106 10.3389/fgene.2020.592769 PMC7982677

[CR157] Jarvis A, Ramirez-Villegas J, Herrera Campo BV, Navarro-Racines C (2012) Is cassava the answer to African climate change adaptation? Trop Plant Biol 5(1):9–29. 10.1007/s12042-012-9096-7

[CR158] Jha PK, Beebe S, Alvarez-Toro P, Mukankusi C, Ramirez-Villegas J (2023) Characterizing patterns of seasonal drought stress for use in common bean breeding in East Africa under present and future climates. Agric for Meteorol 342:109735. 10.1016/j.agrformet.2023.109735 38020492 10.1016/j.agrformet.2023.109735 PMC10636599

[CR159] Ji F, Li F, Hao D, Shiklomanov AN, Yang X, Townsend PA, Dashti H, Nakaji T, Kovach KR, Liu H, Luo M, Chen M (2024a) Unveiling the transferability of PLSR models for leaf trait estimation: lessons from a comprehensive analysis with a novel global dataset. New Phytol 243(1):111–131. 10.1111/nph.19807 38708434 10.1111/nph.19807

[CR160] Ji N, Liu Z, She H, Xu Z, Zhang H, Fang Z, Qian W (2024b) A genome-wide association study reveals the genetic mechanisms of nutrient accumulation in spinach. Genes. 10.3390/genes15020172 38397162 10.3390/genes15020172 PMC10887921

[CR161] Jiang Y, Snider JL, Li C, Rains GC, Paterson AH (2020) Ground based hyperspectral imaging to characterize canopy-level photosynthetic activities. Remote Sens. 10.3390/rs12020315

[CR162] Jin X, Kumar L, Li Z, Feng H, Xu X, Yang G, Wang J (2018) A review of data assimilation of remote sensing and crop models. Eur J Agron 92:141–152. 10.1016/j.eja.2017.11.002

[CR163] Jones JW, Hoogenboom G, Porter CH, Boote KJ, Batchelor WD, Hunt LA, Wilkens PW, Singh U, Gijsman AJ, Ritchie JT (2003) The DSSAT cropping system model. Eur J Agron 18(3):235–265. 10.1016/S1161-0301(02)00107-7

[CR164] Joshi V, Joshi M, Penalosa A (2020) Comparative analysis of tissue-specific transcriptomic responses to nitrogen stress in spinach (*Spinacia oleracea*). PLoS ONE 15(5):e0232011. 10.1371/journal.pone.0232011 32374731 10.1371/journal.pone.0232011 PMC7202632

[CR165] Joshi V, Shi A, Mishra AK, Gill H, DiPiazza J (2022) Genetic dissection of nitrogen induced changes in the shoot and root biomass of spinach. Sci Rep 12(1):13751. 10.1038/s41598-022-18134-7 35962022 10.1038/s41598-022-18134-7 PMC9374745

[CR166] Joshi A, Guevara D, Earles M (2023) Standardizing and centralizing datasets for efficient training of agricultural deep learning models. Plant Phenomics 5:0084. 10.34133/plantphenomics.0084 37680999 10.34133/plantphenomics.0084 PMC10482164

[CR167] Jumper J, Evans R, Pritzel A, Green T, Figurnov M, Ronneberger O, Tunyasuvunakool K, Bates R, Žídek A, Potapenko A, Bridgland A, Meyer C, Kohl SAA, Ballard AJ, Cowie A, Romera-Paredes B, Nikolov S, Jain R, Adler J, Back T, Petersen S, Reiman D, Clancy E, Zielinski M, Steinegger M, Pacholska M, Berghammer T, Bodenstein S, Silver D, Vinyals O, Senior AW, Kavukcuoglu K, Kohli P, Hassabis D (2021) Highly accurate protein structure prediction with AlphaFold. Nature 596(7873):583–589. 10.1038/s41586-021-03819-2 34265844 10.1038/s41586-021-03819-2 PMC8371605

[CR168] Jung K-H, An G, Ronald PC (2008) Towards a better bowl of rice: assigning function to tens of thousands of rice genes. Nat Rev Genet 9(2):91–101. 10.1038/nrg2286 18160965 10.1038/nrg2286

[CR169] Kandel SL, Mou B, Shishkoff N, Shi A, Subbarao KV, Klosterman SJ (2019) Spinach downy mildew: advances in our understanding of the disease cycle and prospects for disease management. Plant Dis 103(5):791–803. 10.1094/PDIS-10-18-1720-FE 30939071 10.1094/PDIS-10-18-1720-FE

[CR170] Kandel SL, Hulse-Kemp AM, Stoffel K, Koike ST, Shi A, Mou B, Van Deynze A, Klosterman SJ (2020) Transcriptional analyses of differential cultivars during resistant and susceptible interactions with *Peronospora effusa*, the causal agent of spinach downy mildew. Sci Rep 10:6719. 10.1038/s41598-020-63668-3 32317662 10.1038/s41598-020-63668-3 PMC7174412

[CR171] Kasampalis DA, Alexandridis TK, Deva C, Challinor A, Moshou D, Zalidis G (2018) Contribution of remote sensing on crop models: a review. J Imaging. 10.3390/jimaging4040052

[CR172] Katuuramu DN, Hart JP, Porch TG, Grusak MA, Glahn RP, Cichy KA (2018) Genome-wide association analysis of nutritional composition-related traits and iron bioavailability in cooked dry beans (*Phaseolus vulgaris* L.). Mol Breed 38(4):44. 10.1007/s11032-018-0798-x

[CR173] Kawamura K, Asai H, Yasuda T, Khanthavong P, Soisouvanh P, Phongchanmixay S (2020) Field phenotyping of plant height in an upland rice field in Laos using low-cost small unmanned aerial vehicles (UAVs). Plant Prod Sci 23(4):452–465. 10.1080/1343943X.2020.1766362

[CR174] Keating BA, Carberry PS, Hammer GL, Probert ME, Robertson MJ, Holzworth D, Huth NI, Hargreaves JNG, Meinke H, Hochman Z, McLean G, Verburg K, Snow V, Dimes JP, Silburn M, Wang E, Brown S, Bristow KL, Asseng S, Chapman S, McCown RL, Freebairn DM, Smith CJ (2003) An overview of APSIM, a model designed for farming systems simulation. Eur J Agron 18(3):267–288. 10.1016/S1161-0301(02)00108-9

[CR175] Keller EF (1984) A feeling for the organism : the life and work of Barbara McClintock. Freeman, W.H

[CR176] Khush GS (2005) What it will take to feed 5.0 billion rice consumers in 2030. Plant Mol Biol 59(1):1–6. 10.1007/s11103-005-2159-5 16217597 10.1007/s11103-005-2159-5

[CR177] Kick DR, Wallace JG, Schnable JC, Kolkman JM, Alaca B, Beissinger TM, Edwards J, Ertl D, Flint-Garcia S, Gage JL, Hirsch CN, Knoll JE, de Leon N, Lima DC, Moreta DE, Singh MP, Thompson A, Weldekidan T, Washburn JD (2023) Yield prediction through integration of genetic, environment, and management data through deep learning. G3 Genes Genomes Genetics 13(4):006. 10.1093/g3journal/jkad006 10.1093/g3journal/jkad006 PMC10085787 36625555

[CR178] Kidmose U, Knuthsen P, Edelenbos M, Justesen U, Hegelund E (2001) Carotenoids and flavonoids in organically grown spinach (*Spinacia oleracea* L) genotypes after deep frozen storage. J Sci Food Agric 81(9):918–923. 10.1002/jsfa.902

[CR179] Ko J, Shin T, Kang J, Baek J, Sang W-G (2024) Combining machine learning and remote sensing-integrated crop modeling for rice and soybean crop simulation. Front Plant Sci. 10.3389/fpls.2024.1320969 38410726 10.3389/fpls.2024.1320969 PMC10894942

[CR180] Koh E, Charoenprasert S, Mitchell AE (2012) Effect of organic and conventional cropping systems on ascorbic acid, vitamin C, flavonoids, nitrate, and oxalate in 27 varieties of spinach (*Spinacia oleracea* L.). J Agric Food Chem 60(12):3144–3150. 10.1021/jf300051f 22393895 10.1021/jf300051f

[CR181] Krause MR, González-Pérez L, Crossa J, Pérez-Rodríguez P, Montesinos-López O, Singh RP, Dreisigacker S, Poland J, Rutkoski J, Sorrells M, Gore MA, Mondal S (2019) Hyperspectral reflectance-derived relationship matrices for genomic prediction of grain yield in wheat. G3 Genes|genomes|genetics 9(4):1231–1247. 10.1534/g3.118.200856 30796086 10.1534/g3.118.200856 PMC6469421

[CR182] Kumagai E, Burroughs CH, Pederson TL, Montes CM, Peng B, Kimm H, Guan K, Ainsworth EA, Bernacchi CJ (2022) Predicting biochemical acclimation of leaf photosynthesis in soybean under in-field canopy warming using hyperspectral reflectance. Plant Cell Environ 45(1):80–94. 10.1111/pce.14204 34664281 10.1111/pce.14204

[CR183] Kumar R, Das SP, Choudhury BU, Kumar A, Prakash NR, Verma R, Chakraborti M, Devi AG, Bhattacharjee B, Das R, Das B, Devi HL, Das B, Rawat S, Mishra VK (2024) Advances in genomic tools for plant breeding: harnessing DNA molecular markers, genomic selection, and genome editing. Biol Res 57(1):80. 10.1186/s40659-024-00562-6 39506826 10.1186/s40659-024-00562-6 PMC11542492

[CR184] Lane HM, Murray SC (2021) High throughput can produce better decisions than high accuracy when phenotyping plant populations. Crop Sci 61(5):3301–3313. 10.1002/csc2.20514

[CR185] Langridge P, Fleury D (2011) Making the most of ‘omics’ for crop breeding. Trends Biotechnol 29(1):33–40. 10.1016/j.tibtech.2010.09.006 21030098 10.1016/j.tibtech.2010.09.006

[CR186] Lasdun V, Güereña D, Ortiz-Crespo B, Mutuvi S, Selvaraj M, Assefa T (2024) Participatory AI for inclusive crop improvement. Agric Syst 220:104054. 10.1016/j.agsy.2024.104054 39473987 10.1016/j.agsy.2024.104054 PMC11513333

[CR187] Lee J, Feng J, Campbell KB, Scheffler BE, Garrett WM, Thibivilliers S, Stacey G, Naiman DQ, Tucker ML, Pastor-Corrales MA, Cooper B (2009) Quantitative proteomic analysis of bean plants infected by a virulent and avirulent obligate rust fungus. Mol Cell Proteomics 8(1):19–31. 10.1074/mcp.M800156-MCP200 18755735 10.1074/mcp.M800156-MCP200

[CR188] Lesjak M, Srai KS (2019) Role of Dietary Flavonoids in Iron Homeostasis. Pharmaceuticals. 10.3390/ph12030119 31398897 10.3390/ph12030119 PMC6789581

[CR189] Li G, Jain R, Chern M, Pham NT, Martin JA, Wei T, Schackwitz WS, Lipzen AM, Duong PQ, Jones KC, Jiang L, Ruan D, Bauer D, Peng Y, Barry KW, Schmutz J, Ronald PC (2017a) The sequences of 1504 mutants in the model rice variety Kitaake facilitate rapid functional genomic studies. Plant Cell 29(6):1218–1231. 10.1105/tpc.17.00154 28576844 10.1105/tpc.17.00154 PMC5502455

[CR190] Li T, Angeles O, Marcaida M, Manalo E, Manalili MP, Radanielson A, Mohanty S (2017b) From ORYZA2000 to ORYZA (v3): An improved simulation model for rice in drought and nitrogen-deficient environments. Agric for Meteorol 237–238:246–256. 10.1016/j.agrformet.2017.02.025 28469286 10.1016/j.agrformet.2017.02.025 PMC5391805

[CR191] Li S, Fleisher D, Timlin D, Reddy VR, Wang Z, McClung A (2020) Evaluation of different crop models for simulating rice development and yield in the U.S. Mississippi Delta. Agronomy 10(12):1905. 10.3390/agronomy10121905

[CR192] Li D, Quan C, Song Z, Li X, Yu G, Li C, Muhammad A (2021a) High-throughput plant phenotyping platform (HT3P) as a novel tool for estimating agronomic traits from the lab to the field. Front Bioeng Biotechnol. 10.3389/fbioe.2020.623705 33520974 10.3389/fbioe.2020.623705 PMC7838587

[CR193] Li X, Guo T, Wang J, Bekele WA, Sukumaran S, Vanous AE, McNellie JP, Tibbs-Cortes LE, Lopes MS, Lamkey KR, Westgate ME, McKay JK, Archontoulis SV, Reynolds MP, Tinker NA, Schnable PS, Yu J (2021b) An integrated framework reinstating the environmental dimension for GWAS and genomic selection in crops. Mol Plant 14(6):874–887. 10.1016/j.molp.2021.03.010 33713844 10.1016/j.molp.2021.03.010

[CR194] Li Q, Gao H, Zhang X, Ni J, Mao H (2022a) Describing lettuce growth using morphological features combined with nonlinear models. Agronomy. 10.3390/agronomy12040860

[CR195] Li S, Fleisher DH, Timlin D, Barnaby J, Sun W, Wang Z, Reddy VR (2022b) Improving simulations of rice in response to temperature and CO2. Agronomy 12(12):2927. 10.3390/agronomy12122927

[CR196] Li W, Wu W, Yu M, Tao H, Yao X, Cheng T, Zhu Y, Cao W, Tian Y (2023) Monitoring rice grain protein accumulation dynamics based on UAV multispectral data. Field Crops Res 294:108858. 10.1016/j.fcr.2023.108858

[CR197] Li D, Wang Q, Tian Y, Lyv X, Zhang H, Hong H, Gao H, Li Y-F, Zhao C, Wang J, Wang R, Yang J, Liu B, Schnable PS, Schnable JC, Li Y-H, Qiu L-J (2024a) TWAS facilitates gene-scale trait genetic dissection through gene expression, structural variations, and alternative splicing in soybean. Plant Commun. 10.1016/j.xplc.2024.101010 38918950 10.1016/j.xplc.2024.101010 PMC11573905

[CR198] Li S, Fleisher DH, Barnaby JY (2024b) Quantifying the impact of climate change and extreme heat on rice in the United States. Agric for Meteorol 355:110145. 10.1016/j.agrformet.2024.110145

[CR199] Liang Z, Qiu Y, Schnable JC (2020) Genome-phenome wide association in maize and *Arabidopsis* identifies a common molecular and evolutionary signature. Mol Plant 13(6):907–922. 10.1016/j.molp.2020.03.003 32171733 10.1016/j.molp.2020.03.003

[CR200] Lichtenthaler HK, Buschmann C (2001) Chlorophylls and carotenoids: measurement and characterization by UV-VIS spectroscopy. Curr Protoc Food Anal Chem. 10.1002/0471142913.faf0403s01

[CR201] Lichtenthaler HK (1987) [34] Chlorophylls and carotenoids: Pigments of photosynthetic biomembranes. In Methods in Enzymology (Vol 148, pp 350–382). Academic Press. 10.1016/0076-6879(87)48036-1

[CR202] Liu F, Wang P, Zhang X, Li X, Yan X, Fu D, Wu G (2018) The genetic and molecular basis of crop height based on a rice model. Planta 247(1):1–26. 10.1007/s00425-017-2798-1 29110072 10.1007/s00425-017-2798-1

[CR203] Liu C, Chen Z, Shao Y, Chen J, Hasi T, Pan H (2019) Research advances of SAR remote sensing for agriculture applications: a review. J Integr Agric 18(3):506–525. 10.1016/S2095-3119(18)62016-7

[CR204] Liu L-W, Lu C-T, Wang Y-M, Lin K-H, Ma X, Lin W-S (2022) Rice (*Oryza sativa* L.) growth modeling based on growth degree day (GDD) and artificial intelligence algorithms. Agriculture (Basel). 10.3390/agriculture12010059

[CR205] Lobell DB, Burke MB (2010) On the use of statistical models to predict crop yield responses to climate change. Agric for Meteorol 150(11):1443–1452. 10.1016/j.agrformet.2010.07.008

[CR206] Lopez-Cruz M, Olson E, Rovere G, Crossa J, Dreisigacker S, Mondal S, Singh R, Campos G (2020) Regularized selection indices for breeding value prediction using hyper-spectral image data. Sci Rep 10(1):8195. 10.1038/s41598-020-65011-2 32424224 10.1038/s41598-020-65011-2 PMC7235263

[CR207] Lu B, Dao PD, Liu J, He Y, Shang J (2020) Recent advances of hyperspectral imaging technology and applications in agriculture. Remote Sens. 10.3390/rs12162659

[CR208] Ly D, Huet S, Gauffreteau A, Rincent R, Touzy G, Mini A, Jannink J-L, Cormier F, Paux E, Lafarge S, Le Gouis J, Charmet G (2018) Whole-genome prediction of reaction norms to environmental stress in bread wheat (*Triticum aestivum* L.) by genomic random regression. Field Crops Res 216:32–41. 10.1016/j.fcr.2017.08.020

[CR209] Ma X, Yu L, Fatima M, Wadlington WH, Hulse-Kemp AM, Zhang X, Zhang S, Xu X, Wang J, Huang H, Lin J, Deng B, Liao Z, Yang Z, Ma Y, Tang H, Van Deynze A, Ming R (2022) The spinach YY genome reveals sex chromosome evolution, domestication, and introgression history of the species. Genome Biol 23(1):75. 10.1186/s13059-022-02633-x 35255946 10.1186/s13059-022-02633-x PMC8902716

[CR210] Madurapperumage A, Naser MZ, Boatwright L, Bridges W, Vandemark G, Thavarajah D (2024) High-throughput phenotyping platforms for pulse crop biofortification. Plants People Planet. 10.1002/ppp3.10568

[CR211] Magney TS (2025) Hyperspectral reflectance integrates key traits for predicting leaf metabolism. New Phytol 246(2):383–385. 10.1111/nph.20345 39673249 10.1111/nph.20345 PMC11923394

[CR212] Magney TS, Eitel JUH, Huggins DR, Vierling LA (2016) Proximal NDVI derived phenology improves in-season predictions of wheat quantity and quality. Agric Meteorol 217:46–60. 10.1016/j.agrformet.2015.11.009

[CR822] Magney, T. S., Eitel, J. U. H., Vierling, L. A. (2017).Mapping wheat nitrogen uptake from RapidEye vegetation indices. Precision Agriculture, 18(4),429–451 10.1007/s11119-016-9463-8

[CR213] Mahadevan K, Punitha A, Suresh J (2024) A novel rice plant leaf diseases detection using deep spectral generative adversarial neural network. Int J Cognit Comput Eng 5:237–249. 10.1016/j.ijcce.2024.05.004

[CR214] Mahmood U, Li X, Fan Y, Chang W, Niu Y, Li J, Qu C, Lu K (2022) Multi-omics revolution to promote plant breeding efficiency. Front Plant Sci. 10.3389/fpls.2022.1062952 36570904 10.3389/fpls.2022.1062952 PMC9773847

[CR215] Malosetti M, Bustos-Korts D, Boer MP, van Eeuwijk FA (2016) Predicting responses in multiple environments: issues in relation to genotype × environment interactions. Crop Sci 56(5):2210–2222. 10.2135/cropsci2015.05.0311

[CR218] Marshall J, Vargas A, Bett K (2024) B vitamin quantification in lentil seed tissues using ultra-performance liquid chromatography-selected reaction monitoring mass spectrometry. Food Chem 430:136922. 10.1016/j.foodchem.2023.136922 37517945 10.1016/j.foodchem.2023.136922

[CR219] McClean PE, Raatz B (2017) Common bean genomes: mining new knowledge of a major societal crop. In: Pérez de la Vega M, Santalla M, Marsolais F (eds) The common bean genome. Springer International Publishing, New York, pp 129–145. 10.1007/978-3-319-63526-2_6

[CR220] Melandri G, Thorp KR, Broeckling C, Thompson AL, Hinze L, Pauli D (2021) Assessing drought and heat stress-induced changes in the cotton leaf metabolome and their relationship with hyperspectral reflectance. Front Plant Sci 12:751868. 10.3389/fpls.2021.751868 34745185 10.3389/fpls.2021.751868 PMC8569624

[CR221] Meléndez-Martínez AJ, Mandić AI, Bantis F, Böhm V, Borge GIA, Brnčić M, Bysted A, Cano MP, Dias MG, Elgersma A, Fikselová M, García-Alonso J, Giuffrida D, Gonçalves VSS, Hornero-Méndez D, Kljak K, Lavelli V, Manganaris GA, Mapelli-Brahm P, Marounek M, Olmedilla-Alonso B, Periago-Castón MJ, Pintea A, Sheehan JJ, Tumbas Šaponjac V, Valšíková-Frey M, Meulebroek LV, O’Brien N (2022) A comprehensive review on carotenoids in foods and feeds: status quo, applications, patents, and research needs. Crit Rev Food Sci Nutr. 10.1080/10408398.2020.1867959 33399015 10.1080/10408398.2020.1867959

[CR222] Mercadante AZ, Rodrigues DB, Petry FC, Mariutti LRB (2017) Carotenoid esters in foods—a review and practical directions on analysis and occurrence. Food Res Int 99:830–850. 10.1016/j.foodres.2016.12.018 28847421 10.1016/j.foodres.2016.12.018

[CR223] Messina CD, Technow F, Tang T, Totir R, Gho C, Cooper M (2018) Leveraging biological insight and environmental variation to improve phenotypic prediction: integrating crop growth models (CGM) with whole genome prediction (WGP). Eur J Agron 100:151–162. 10.1016/j.eja.2018.01.007

[CR224] Millet EJ, Welcker C, Kruijer W, Negro S, Coupel-Ledru A, Nicolas SD, Laborde J, Bauland C, Praud S, Ranc N, Presterl T, Tuberosa R, Bedo Z, Draye X, Usadel B, Charcosset A, Van Eeuwijk F, Tardieu F (2016) Genome-wide analysis of yield in europe: allelic effects vary with drought and heat scenarios. Plant Physiol 172(2):749–764. 10.1104/pp.16.00621 27436830 10.1104/pp.16.00621 PMC5047082

[CR225] Millet EJ, Kruijer W, Coupel-Ledru A, Alvarez Prado S, Cabrera-Bosquet L, Lacube S, Charcosset A, Welcker C, van Eeuwijk F, Tardieu F (2019) Genomic prediction of maize yield across European environmental conditions. Nat Genet 51(6):952–956. 10.1038/s41588-019-0414-y 31110353 10.1038/s41588-019-0414-y

[CR226] Moghaddam SM, Oladzad A, Koh C, Ramsay L, Hart JP, Mamidi S, Hoopes G, Sreedasyam A, Wiersma A, Zhao D, Grimwood J, Hamilton JP, Jenkins J, Vaillancourt B, Wood JC, Schmutz J, Kagale S, Porch T, Bett KE, Buell CR, McClean PE (2021) The tepary bean genome provides insight into evolution and domestication under heat stress. Nat Commun 12(1):2638. 10.1038/s41467-021-22858-x 33976152 10.1038/s41467-021-22858-x PMC8113540

[CR227] Montesinos-López OA, Montesinos-López A, Crossa J, Toledo FH, Pérez-Hernández O, Eskridge KM, Rutkoski J (2016) A genomic Bayesian multi-trait and multi-environment model. G3 Genes|genomes|genetics 6(9):2725–2744. 10.1534/g3.116.032359 27342738 10.1534/g3.116.032359 PMC5015931

[CR228] Montesinos-López OA, Crespo-Herrera L, Saint Pierre C, Bentley AR, de la Rosa-Santamaria R, Ascencio-Laguna JA, Agbona A, Gerard GS, Montesinos-López A, Crossa J (2023) Do feature selection methods for selecting environmental covariables enhance genomic prediction accuracy? Front Genet. 10.3389/fgene.2023.1209275 37554404 10.3389/fgene.2023.1209275 PMC10405933

[CR229] Montesinos-López OA, Crespo-Herrera L, Pierre CS, Cano-Paez B, Huerta-Prado GI, Mosqueda-González BA, Ramos-Pulido S, Gerard G, Alnowibet K, Fritsche-Neto R, Montesinos-López A, Crossa J (2024a) Feature engineering of environmental covariates improves plant genomic-enabled prediction. Front Plant Sci. 10.3389/fpls.2024.1349569 38812738 10.3389/fpls.2024.1349569 PMC11135473

[CR230] Montesinos-López OA, Herr AW, Crossa J, Montesinos-López A, Carter AH (2024b) Enhancing winter wheat prediction with genomics, phenomics and environmental data. BMC Genomics 25(1):544. 10.1186/s12864-024-10438-4 38822262 10.1186/s12864-024-10438-4 PMC11143639

[CR231] Monteverde E, Gutierrez L, Blanco P, Pérez de Vida F, Rosas JE, Bonnecarrère V, Quero G, McCouch S (2019) Integrating molecular markers and environmental covariates to interpret genotype by environment interaction in rice (*Oryza sativa* L.) grown in subtropical areas. G3 Genes Genomes Genetics 9(5):1519–1531. 10.1534/g3.119.400064 30877079 10.1534/g3.119.400064 PMC6505146

[CR232] Morais Júnior OP, Duarte JB, Breseghello F, Coelho ASG, Morais OP, Magalhães Júnior AM (2018) Single-step reaction norm models for genomic prediction in multienvironment recurrent selection trials. Crop Sci 58(2):592–607. 10.2135/cropsci2017.06.0366

[CR233] Morelli L, Perez-Colao P, Reig-Lopez D, Di X, Llorente B, Rodriguez-Concepcion M (2024) Boosting pro-vitamin A content and bioaccessibility in leaves by combining engineered biosynthesis and storage pathways with high-light treatments. Plant J. 10.1111/tpj.16964 39121193 10.1111/tpj.16964

[CR234] Mou B (2005) Genetic Variation of Beta-carotene and Lutein Contents in Lettuce. 10.21273/JASHS.130.6.870

[CR235] Mou B (2008) Lettuce. In: Vegetables I: Asteraceae, Brassicaceae, Chenopodicaceae, and Cucurbitaceae. Springer

[CR236] Mourtzinis S, Rattalino Edreira JI, Conley SP, Grassini P (2017) From grid to field: assessing quality of gridded weather data for agricultural applications. Eur J Agron 82:163–172. 10.1016/j.eja.2016.10.013

[CR831] Mu Q, Guo T, Li X, Yu J (2022) Phenotypic plasticity in plant height shaped by interaction between genetic loci and diurnal temperature range. New Phytol 233:1768–1779. 10.1111/nph.17904 34870847 10.1111/nph.17904

[CR237] Myers SS, Smith MR, Guth S, Golden CD, Vaitla B, Mueller ND, Dangour AD, Huybers P (2017) Climate change and global food systems: potential impacts on food security and undernutrition. Annu Rev Public Health 38(1):259–277. 10.1146/annurev-publhealth-031816-044356 28125383 10.1146/annurev-publhealth-031816-044356

[CR238] Newton AC, Johnson SN, Gregory PJ (2011) Implications of climate change for diseases, crop yields and food security. Euphytica 179(1):3–18. 10.1007/s10681-011-0359-4

[CR239] Neyhart JL, Tiede T, Lorenz AJ, Smith KP (2017) Evaluating methods of updating training data in long-term genomewide selection. G3 Genes|genomes|genetics 7(5):1499–1510. 10.1534/g3.117.040550 28315831 10.1534/g3.117.040550 PMC5427505

[CR240] Nomura K, Takada A, Kunishige H, Ozaki Y, Okayasu T, Yasutake D, Kitano M (2020) Long-term and continuous measurement of canopy photosynthesis and growth of spinach. Environ Control Biol 58(2):21–29. 10.2525/ecb.58.21

[CR241] O’Rourke JA, Iniguez LP, Fu F, Bucciarelli B, Miller SS, Jackson SA, McClean PE, Li J, Dai X, Zhao PX, Hernandez G, Vance CP (2014) An RNA-Seq based gene expression atlas of the common bean. BMC Genomics 15(1):866. 10.1186/1471-2164-15-866 25283805 10.1186/1471-2164-15-866 PMC4195886

[CR242] OECD & Food and Agriculture Organization of the United Nations (2023) OECD-FAO agricultural outlook 2023–2032. OECD. 10.1787/08801ab7-en

[CR243] Ojo MO, Zahid A, Masabni JG (2024) Estimating hydroponic lettuce phenotypic parameters for efficient resource allocation. Comput Electron Agric 218:108642. 10.1016/j.compag.2024.108642

[CR244] Onogi A, Watanabe M, Mochizuki T, Hayashi T, Nakagawa H, Hasegawa T, Iwata H (2016) Toward integration of genomic selection with crop modelling: the development of an integrated approach to predicting rice heading dates. Theor Appl Genet 129(4):805–817. 10.1007/s00122-016-2667-5 26791836 10.1007/s00122-016-2667-5

[CR245] Oppelt NM (2010) Use of remote sensing data to assist crop modeling. J Appl Remote Sens 4(1):041896. 10.1117/1.3491191

[CR246] P.vulgaris v2.1: Phytozome. (n.d.). Retrieved May 16, 2025, from https://phytozome-next.jgi.doe.gov/info/Pvulgaris_v2_1

[CR247] Pamplona RS, Kim J, Lee JW, Kim CS, Boo K-H (2022) Comparative transcriptome analysis of spinach in response to insect herbivory. Plant Biotechnol Rep 16(1):43–55. 10.1007/s11816-021-00736-8

[CR248] Pandjaitan N, Howard LR, Morelock T, Gil MI (2005) Antioxidant capacity and phenolic content of spinach as affected by genetics and maturation. J Agric Food Chem. 10.1021/jf052077i 16248562 10.1021/jf052077i

[CR249] Parker TA, Palkovic A, Gepts P (2020) Determining the genetic control of common bean early-growth rate using unmanned aerial vehicles. Remote Sens. 10.3390/rs12111748

[CR250] Parra L, Maisonneuve B, Lebeda A, Schut J, Christopoulou M, Jeuken M, McHale L, Truco M-J, Crute I, Michelmore R (2016) Rationalization of genes for resistance to *Bremia lactucae* in lettuce. Euphytica 210(3):309–326. 10.1007/s10681-016-1687-1

[CR251] Parra L, Nortman K, Sah A, Truco MJ, Ochoa O, Michelmore R (2021) Identification and mapping of new genes for resistance to downy mildew in lettuce. Theor Appl Genet 134(2):519–528. 10.1007/s00122-020-03711-z 33128618 10.1007/s00122-020-03711-z PMC7843477

[CR252] Parreira JR, Bouraada J, Fitzpatrick MA, Silvestre S, Bernardes da Silva A, Marques da Silva J, Almeida AM, Fevereiro P, Altelaar AFM, Araújo SS (2016) Differential proteomics reveals the hallmarks of seed development in common bean (*Phaseolus vulgaris* L.). J Proteomics 143:188–198. 10.1016/j.jprot.2016.03.002 26945737 10.1016/j.jprot.2016.03.002

[CR253] Pauli D, Andrade-Sanchez P, Carmo-Silva AE, Gazave E, French AN, Heun J, Hunsaker DJ, Lipka AE, Setter TL, Strand RJ, Thorp KR, Wang S, White JW, Gore MA (2016) Field-based high-throughput plant phenotyping reveals the temporal patterns of quantitative trait loci associated with stress-responsive traits in cotton. G3 Genes|genomes|genetics 6(4):865–879. 10.1534/g3.115.023515 26818078 10.1534/g3.115.023515 PMC4825657

[CR254] Pearson S, Wheeler TR, Hadley P, Wheldon AE (1997) A validated model to predict the effects of environment on the growth of lettuce (*Lactuca sativa* L.): implications for climate change. J Hortic Sci 72(4):503–517. 10.1080/14620316.1997.11515538

[CR255] Peng B, Guan K, Tang J, Ainsworth EA, Asseng S, Bernacchi CJ, Cooper M, Delucia EH, Elliott JW, Ewert F, Grant RF, Gustafson DI, Hammer GL, Jin Z, Jones JW, Kimm H, Lawrence DM, Li Y, Lombardozzi DL, Marshall-Colon A, Messina CD, Ort DR, Schnable JC, Vallejos CE, Wu A, Yin X, Zhou W (2020) Towards a multiscale crop modelling framework for climate change adaptation assessment. Nat Plants 6(4):338–348. 10.1038/s41477-020-0625-3 32296143 10.1038/s41477-020-0625-3

[CR256] Pérez-Rodríguez P, Crossa J, Bondalapati K, De Meyer G, Pita F, Campos G (2015) A pedigree-based reaction norm model for prediction of cotton yield in multienvironment trials. Crop Sci 55(3):1143–1151. 10.2135/cropsci2014.08.0577

[CR257] Perry G, Dinatale C, Xie W, Navabi A, Reinprecht Y, Crosby W, Yu K, Shi C, Pauls KP (2013) A comparison of the molecular organization of genomic regions associated with resistance to common bacterial blight in two *Phaseolus vulgaris* genotypes. Front Plant Sci 4:318. 10.3389/fpls.2013.00318 24009615 10.3389/fpls.2013.00318 PMC3756299

[CR258] Peters-Clarke TM, Coon JJ, Riley NM (2024) Instrumentation at the leading edge of proteomics. Anal Chem. 10.1021/acs.analchem.3c04497 38738990 10.1021/acs.analchem.3c04497 PMC11996003

[CR260] Piepho H-P, Williams E (2024) Factor-analytic variance-covariance structures for prediction into a target population of environments. Biom J 66(6):e202400008. 10.1002/bimj.202400008 39049627 10.1002/bimj.202400008

[CR261] Pierrat ZA, Magney TS, Richardson WP, Runkle BRK, Diehl JL, Yang X, Woodgate W, Smith WK, Johnston MR, Ginting YRS, Koren G, Albert LP, Kibler CL, Morgan BE, Barnes M, Uscanga A, Devine C, Javadian M, Meza K, Julitta T, Tagliabue G, Dannenberg MP, Antala M, Wong CYS, Santos ALD, Hufkens K, Marrs JK, Stovall AEL, Liu Y, Fisher JB, Gamon JA, Cawse-Nicholson K (2025) Proximal remote sensing: an essential tool for bridging the gap between high-resolution ecosystem monitoring and global ecology. New Phytol 246(2):419–436. 10.1111/nph.20405 39853577 10.1111/nph.20405 PMC11923411

[CR262] Poland JA, Nelson RJ (2011) In the eye of the beholder: the effect of rater variability and different rating scales on QTL mapping. Phytopathology® 101(2):290–298. 10.1094/PHYTO-03-10-0087 20955083 10.1094/PHYTO-03-10-0087

[CR263] Porra RJ, Thompson WA, Kriedemann PE (1989) Determination of accurate extinction coefficients and simultaneous equations for assaying chlorophylls a and b extracted with four different solvents: verification of the concentration of chlorophyll standards by atomic absorption spectroscopy. Biochimica Et Biophysica Acta (BBA) Bioenergetics. 10.1016/S0005-2728(89)80347-0

[CR264] PRISM Group, Oregon State University, https://prism.oregonstate.edu

[CR265] Project-AgML/AgML. (2024). [Python]. AgML. https://github.com/Project-AgML/AgML (Original work published 2021)

[CR266] Qin J, Shi A, Mou B, Grusak MA, Weng Y, Ravelombola W, Bhattarai G, Dong L, Yang W (2017) Genetic diversity and association mapping of mineral element concentrations in spinach leaves. BMC Genomics 18(1):941. 10.1186/s12864-017-4297-y 29202697 10.1186/s12864-017-4297-y PMC5715654

[CR267] Raboy V, Gibson RS, Bailey KB, King JC (2020) Comparison of four methods for phytate analysis in plant-based foods. J Food Compos Anal 90:103481. 10.1016/j.jfca.2020.103481

[CR268] Radin JW, Lu Z, Percy RG, Zeiger E (1994) Genetic variability for stomatal conductance in Pima cotton and its relation to improvements of heat adaptation. Proc Natl Acad Sci 91(15):7217–7221. 10.1073/pnas.91.15.7217 11607487 10.1073/pnas.91.15.7217 PMC44370

[CR269] Raja P, Olenskyj A, Kamangir H, Earles M (2021) Simultaneously Predicting Multiple Plant Traits from Multiple Sensors via Deformable CNN Regression. arXiv.Org. https://arxiv.org/abs/2112.03205v1

[CR270] Ramirez-Cabral NYZ, Kumar L, Taylor S (2016) Crop niche modeling projects major shifts in common bean growing areas. Agric for Meteorol 218:102–113. 10.1016/j.agrformet.2015.12.002

[CR271] Ramirez-Villegas J, Challinor A (2012) Assessing relevant climate data for agricultural applications. Agric for Meteorol 161:26–45. 10.1016/j.agrformet.2012.03.015

[CR272] Rast M, Ananasso C, Bach H, Ben-Dor E, Chabrillat S, Colombo R, Del Bello U, Feret JB, Giardino C, Green RO (2019) Copernicus hyperspectral imaging mission for the environment: Mission requirements document. https://research.utwente.nl/files/228969030/Copernicus_CHIME_MRD_v2.1_Issued20190723.pdf

[CR273] Reyes-Chin-Wo S, Wang Z, Yang X, Kozik A, Arikit S, Song C, Xia L, Froenicke L, Lavelle DO, Truco M-J, Xia R, Zhu S, Xu C, Xu H, Xu X, Cox K, Korf I, Meyers BC, Michelmore RW (2017) Genome assembly with in vitro proximity ligation data and whole-genome triplication in lettuce. Nat Commun 8(1):14953. 10.1038/ncomms14953 28401891 10.1038/ncomms14953 PMC5394340

[CR274] Rim EY, Garrett OD, Howard AJ, Shim Y, Li Y, Dyke JEV, Packer RC, Ho N, Jain RS, Stewart VJ, Dinesh-Kumar SP, Notwell JH, Ronald PC (2024) Directed evolution of a plant immune receptor for broad spectrum recognition of pathogen effectors (p. 2024.09.30.614878). bioRxiv. 10.1101/2024.09.30.614878

[CR275] Rincent R, Kuhn E, Monod H, Oury F-X, Rousset M, Allard V, Le Gouis J (2017) Optimization of multi-environment trials for genomic selection based on crop models. Theor Appl Genet 130(8):1735–1752. 10.1007/s00122-017-2922-4 28540573 10.1007/s00122-017-2922-4 PMC5511605

[CR276] Rincent R, Charpentier J-P, Faivre-Rampant P, Paux E, Le Gouis J, Bastien C, Segura V (2018) Phenomic selection is a low-cost and high-throughput method based on indirect predictions: proof of concept on wheat and poplar. G3 Genes|genomes|genetics 8(12):3961–3972. 10.1534/g3.118.200760 30373914 10.1534/g3.118.200760 PMC6288839

[CR277] Rincent R, Malosetti M, Ababaei B, Touzy G, Mini A, Bogard M, Martre P, Le Gouis J, van Eeuwijk F (2019) Using crop growth model stress covariates and AMMI decomposition to better predict genotype-by-environment interactions. Theor Appl Genet 132(12):3399–3411. 10.1007/s00122-019-03432-y 31562567 10.1007/s00122-019-03432-y

[CR278] Rippke U, Ramirez-Villegas J, Jarvis A, Vermeulen SJ, Parker L, Mer F, Diekkrüger B, Challinor AJ, Howden M (2016) Timescales of transformational climate change adaptation in sub-Saharan African agriculture. Nat Clim Chang 6(6):605–609. 10.1038/nclimate2947

[CR279] Rivero RM, Mittler R, Blumwald E, Zandalinas SI (2021) Developing climate-resilient crops: Improving plant tolerance to stress combination. Plant J 109(2):373–389. 10.1111/tpj.15483 34482588 10.1111/tpj.15483

[CR280] Robert P, Brault C, Rincent R, Segura V (2022) Phenomic selection: a new and efficient alternative to genomic selection. In: Ahmadi N, Bartholomé J (eds) Genomic prediction of complex traits: methods and protocols. Springer, USA, pp 397–420. 10.1007/978-1-0716-2205-6_14 10.1007/978-1-0716-2205-6_14 35451784

[CR281] Robert P, Le Gouis J, The BreedWheat Consortium, Rincent R. (2020) Combining Crop Growth Modeling With Trait-Assisted Prediction Improved the Prediction of Genotype by Environment Interactions. Frontiers in Plant Science, 11. 10.3389/fpls.2020.00827 10.3389/fpls.2020.00827 PMC7317015 32636859

[CR282] Rogers AR, Holland JB (2022) Environment-specific genomic prediction ability in maize using environmental covariates depends on environmental similarity to training data. G3 Genes Genomes Genetics 12(2):jkab440. 10.1093/g3journal/jkab440 35100364 10.1093/g3journal/jkab440 PMC9245610

[CR283] Rogers AR, Dunne JC, Romay C, Bohn M, Buckler ES, Ciampitti IA, Edwards J, Ertl D, Flint-Garcia S, Gore MA, Graham C, Hirsch CN, Hood E, Hooker DC, Knoll J, Lee EC, Lorenz A, Lynch JP, McKay J, Holland JB (2021) The importance of dominance and genotype-by-environment interactions on grain yield variation in a large-scale public cooperative maize experiment. G3 Genes Genomes Genetics 11(2):jkaa050. 10.1093/g3journal/jkaa050 33585867 10.1093/g3journal/jkaa050 PMC8022981

[CR284] Romano G, Zia S, Spreer W, Sanchez C, Cairns J, Araus JL, Müller J (2011) Use of thermography for high throughput phenotyping of tropical maize adaptation in water stress. Comput Electron Agric 79(1):67–74. 10.1016/j.compag.2011.08.011

[CR285] Runcie D, Cheng H (2019) Pitfalls and remedies for cross validation with multi-trait genomic prediction methods. G3 Genes Genomes Genetics 9(11):3727–3741. 10.1534/g3.119.400598 31511297 10.1534/g3.119.400598 PMC6829121

[CR286] Runcie DE, Qu J, Cheng H, Crawford L (2021) MegaLMM: Mega-scale linear mixed models for genomic predictions with thousands of traits. Genome Biol 22(1):213. 10.1186/s13059-021-02416-w 34301310 10.1186/s13059-021-02416-w PMC8299638

[CR333] Runkle, B. R. K., Barnes, M., Dannenberg, M., Gamon, J. A., Magney, T., Pierrat, Z., Southwick, C. D., Still, C., Woodgate, W. (2025). Near-surface remote sensing applications for a robust, climate-smart measurement, monitoring, and information system (MMIS). Carbon Management, 16(1). 10.1080/17583004.2025.2465361

[CR287] Sadeghi R, Colle M, Smith B (2023) Protein composition of pulses and their protein isolates from different sources and in different isolation pH values using a reverse phase high performance liquid chromatography method. Food Chem 409:135278. 10.1016/j.foodchem.2022.135278 36586270 10.1016/j.foodchem.2022.135278

[CR288] Salavati A, Taleei A, Bushehri AAS, Komatsu S (2012) Analysis of the Proteome of Common Bean (Phaseolus vulgaris L.) Roots after Inoculation with Rhizobium etli. http://Www.Eurekaselect.Com. Retrieved September 23, 2024, from https://www.eurekaselect.com/article/44191 10.2174/092986612801619615 22762188

[CR289] Saleh ASM, Wang P, Wang N, Yang L, Xiao Z (2019) Brown rice versus white rice: nutritional quality, potential health benefits, development of food products, and preservation technologies. Compr Rev Food Sci Food Saf 18(4):1070–1096. 10.1111/1541-4337.12449 33336992 10.1111/1541-4337.12449

[CR290] Schaefer GL, Cosh MH, Jackson TJ (2007) The USDA Natural Resources Conservation Service Soil Climate Analysis Network (SCAN). 10.1175/2007JTECHA930.1

[CR291] Schmutz J, McClean PE, Mamidi S, Wu GA, Cannon SB, Grimwood J, Jenkins J, Shu S, Song Q, Chavarro C, Torres-Torres M, Geffroy V, Moghaddam SM, Gao D, Abernathy B, Barry K, Blair M, Brick MA, Chovatia M, Jackson SA (2014) A reference genome for common bean and genome-wide analysis of dual domestications. Nat Genetics 46(7):707–713. 10.1038/ng.3008 24908249 10.1038/ng.3008 PMC7048698

[CR292] Seidel SJ, Rachmilevitch S, Schütze N, Lazarovitch N (2016) Modelling the impact of drought and heat stress on common bean with two different photosynthesis model approaches. Environ Model Softw 81:111–121. 10.1016/j.envsoft.2016.04.001

[CR293] Selby P, Abbeloos R, Backlund JE, Basterrechea Salido M, Bauchet G, Benites-Alfaro OE, Birkett C, Calaminos VC, Carceller P, Cornut G (2019) BrAPI—an application programming interface for plant breeding applications. Bioinformatics 35(20):4147–4155 30903186 10.1093/bioinformatics/btz190 PMC6792114

[CR294] Sepulcre-Cantó G, Zarco-Tejada PJ, Sobrino JA, Jiménez-Muñoz JC, Villalobos FJ (2005) Spatial variability of crop water stress in an olive grove with high-spatial thermal remote sensing imagery. Brill. 10.3920/978-90-8686-549-9_033

[CR295] Sepulcre-Cantó G, Zarco-Tejada PJ, Jiménez-Muñoz JC, Sobrino JA, de Miguel E, Villalobos FJ (2006) Detection of water stress in an olive orchard with thermal remote sensing imagery. Agric for Meteorol 136(1):31–44. 10.1016/j.agrformet.2006.01.008

[CR296] Shangguan W, Dai Y, Duan Q, Liu B, Yuan H (2014) A global soil data set for earth system modeling. J Adv Model Earth Syst 6(1):249–263. 10.1002/2013MS000293

[CR297] She H, Liu Z, Xu Z, Zhang H, Wu J, Wang X, Cheng F, Charlesworth D, Qian W (2024) Insights into spinach domestication from genome sequences of two wild spinach progenitors, Spinacia turkestanica and Spinacia tetrandra. New Phytol 243(1):477–494. 10.1111/nph.19799 38715078 10.1111/nph.19799

[CR298] Sheikh M, Iqra F, Ambreen H, Pravin KA, Ikra M, Chung YS (2024) Integrating artificial intelligence and high-throughput phenotyping for crop improvement. J Integr Agric 23(6):1787–1802. 10.1016/j.jia.2023.10.019

[CR299] Shi A, Qin J, Mou B, Correll J, Weng Y, Brenner D, Feng C, Motes D, Yang W, Dong L, Bhattarai G, Ravelombola W (2017) Genetic diversity and population structure analysis of spinach by single-nucleotide polymorphisms identified through genotyping-by-sequencing. PLoS ONE 12(11):e0188745. 10.1371/journal.pone.0188745 29190770 10.1371/journal.pone.0188745 PMC5708663

[CR300] Shi A, Bhattarai G, Xiong H, Avila CA, Feng C, Liu B, Joshi V, Stein L, Mou B, du Toit LJ, Correll JC (2022) Genome-wide association study and genomic prediction of white rust resistance in USDA GRIN spinach germplasm. Hortic Res 9:uhac069. 10.1093/hr/uhac069 35669703 10.1093/hr/uhac069 PMC9157682

[CR301] Shohag MJI, Wei Y, Yu N, Zhang J, Wang K, Patring J, He Z, Yang X (2011) Natural Variation of folate content and composition in Spinach (Spinacia oleracea) Germplasm. J Agric Food Chem 59(23):12520–12526. 10.1021/jf203442h 22004472 10.1021/jf203442h

[CR302] Shrestha N, Powadi A, Davis J, Ayanlade TT, Liu H, Tross MC, Mathivanan RK, Bares J, Lopez-Corona L, Turkus J, Coffey L, Jubery TZ, Ge Y, Sarkar S, Schnable JC, Ganapathysubramanian B, Schnable PS (n.d.). Plot-level satellite imagery can substitute for UAVs in assessing maize phenotypes across multistate field trials. PLANTS, PEOPLE, PLANET, n/a(n/a). 10.1002/ppp3.10613

[CR303] Silva-Perez V, Molero G, Serbin SP, Condon AG, Reynolds MP, Furbank RT, Evans JR (2018) Hyperspectral reflectance as a tool to measure biochemical and physiological traits in wheat. J Exp Bot 69(3):483–496. 10.1093/jxb/erx421 29309611 10.1093/jxb/erx421 PMC5853784

[CR304] Sinclair TR, Seligman NG (1996) Crop modeling: from infancy to maturity. Agron J 88(5):698–704. 10.2134/agronj1996.00021962008800050004x

[CR305] Singh SP, Miklas PN (2015) Breeding common bean for resistance to common blight: a review. Crop Sci 55(3):971–984. 10.2135/cropsci2014.07.0502

[CR306] Singh U, Ritchie JT (1993) Simulating the Impact of climate change on crop growth and nutrient dynamics using the CERES-rice model. J Agric Meteorol 48(5):819–822. 10.2480/agrmet.48.819

[CR307] Singh A, Serbin SP, McNeil BE, Kingdon CC, Townsend PA (2015) Imaging spectroscopy algorithms for mapping canopy foliar chemical and morphological traits and their uncertainties. Ecol Appl 25(8):2180–2197. 10.1890/14-2098.1 26910948 10.1890/14-2098.1

[CR308] Singh BK, Delgado-Baquerizo M, Egidi E, Guirado E, Leach JE, Liu H, Trivedi P (2023) Climate change impacts on plant pathogens, food security and paths forward. Nat Rev Microbiol 21(10):640–656. 10.1038/s41579-023-00900-7 37131070 10.1038/s41579-023-00900-7 PMC10153038

[CR309] Skamarock WC, Klemp JB, Dudhia J, Gill DO, Liu Z, Berner J, Wang W, Powers JG, Duda MG, Barker DM, Huang XY (2019) A Description of the Advanced Research WRF Version 4. NCAR Tech. Note NCAR/TN-556+STR, 145 pp. 10.5065/1dfh-6p97

[CR311] Smith DT, Potgieter AB, Chapman SC (2021) Scaling up high-throughput phenotyping for abiotic stress selection in the field. Theor Appl Genet 134(6):1845–1866. 10.1007/s00122-021-03864-5 34076731 10.1007/s00122-021-03864-5

[CR313] Soil Survey Staff, Natural Resources Conservation Service, United States Department of Agriculture. Web Soil Survey. Available online at https://websoilsurvey.nrcs.usda.gov/

[CR314] Spindel J, Iwata H (2018) Genomic selection in rice breeding. In: Sasaki T, Ashikari M (eds) Rice genomics, genetics and breeding. Springer, Singapore, pp 473–496. 10.1007/978-981-10-7461-5_24

[CR315] Su W, Tao R, Liu W, Yu C, Yue Z, He S, Lavelle D, Zhang W, Zhang L, An G, Zhang Y, Hu Q, Larkin RM, Michelmore RW, Kuang H, Chen J (2020) Characterization of four polymorphic genes controlling red leaf colour in lettuce that have undergone disruptive selection since domestication. Plant Biotechnol J 18(2):479–490. 10.1111/pbi.13213 31325407 10.1111/pbi.13213 PMC6953203

[CR316] Suzuki N, Rivero RM, Shulaev V, Blumwald E, Mittler R (2014) Abiotic and biotic stress combinations. New Phytol 203(1):32–43. 10.1111/nph.12797 24720847 10.1111/nph.12797

[CR317] Sytar O, Zivcak M, Bruckova K, Brestic M, Hemmerich I, Rauh C, Simko I (2018) Shift in accumulation of flavonoids and phenolic acids in lettuce attributable to changes in ultraviolet radiation and temperature. Sci Hortic 239:193–204. 10.1016/j.scienta.2018.05.020

[CR318] Tang L, Zhu Y, Hannaway D, Meng Y, Liu L, Chen L, Cao W (2009) RiceGrow: A rice growth and productivity model. NJAS Wageningen J Life Sci 57(1):83–92. 10.1016/j.njas.2009.12.003

[CR319] Tao H, Xu S, Tian Y, Li Z, Ge Y, Zhang J, Wang Y, Zhou G, Deng X, Zhang Z, Ding Y, Jiang D, Guo Q, Jin S (2022) Proximal and remote sensing in plant phenomics: 20 years of progress, challenges, and perspectives. Plant Commun. 10.1016/j.xplc.2022.100344 35655429 10.1016/j.xplc.2022.100344 PMC9700174

[CR320] Tardieu F, Cabrera-Bosquet L, Pridmore T, Bennett M (2017) Plant phenomics, from sensors to knowledge. Curr Biol 27(15):R770–R783. 10.1016/j.cub.2017.05.055 28787611 10.1016/j.cub.2017.05.055

[CR321] Technow F, Messina CD, Totir LR, Cooper M (2015) Integrating crop growth models with whole genome prediction through approximate bayesian computation. PLoS ONE 10(6):e0130855. 10.1371/journal.pone.0130855 26121133 10.1371/journal.pone.0130855 PMC4488317

[CR322] The 3, 000 rice genomes project. (2014). The 3,000 rice genomes project. GigaScience, 3(1), 7. 10.1186/2047-217X-3-7 10.1186/2047-217X-3-7 PMC4035669 24872877

[CR323] Thornton PE, Shrestha R, Thornton M et al (2021) Gridded daily weather data for North America with comprehensive uncertainty quantification. Sci Data 8:190. 10.1038/s41597-021-00973-0 34301954 10.1038/s41597-021-00973-0 PMC8302764

[CR324] Thorogood R, Mustonen V, Aleixo A, Aphalo PJ, Asiegbu FO, Cabeza M, Cairns J, Candolin U, Cardoso P, Eronen JT, Hällfors M, Hovatta I, Juslén A, Kovalchuk A, Kulmuni J, Kuula L, Mäkipää R, Ovaskainen O, Pesonen A-K, Vanhatalo J (2023) Understanding and applying biological resilience, from genes to ecosystems. Npj Biodiversity 2(1):1–13. 10.1038/s44185-023-00022-6 39242840 10.1038/s44185-023-00022-6 PMC11332022

[CR325] Thorp KR, Calleja S, Pauli D, Thompson AL, Elshikha DE (2022) Agronomic outcomes of precision irrigation management technologies with varying complexity. J ASABE 65(1):135–150. 10.13031/ja.14950

[CR326] Tian L, Xue B, Wang Z, Li D, Yao X, Cao Q, Zhu Y, Cao W, Cheng T (2021) Spectroscopic detection of rice leaf blast infection from asymptomatic to mild stages with integrated machine learning and feature selection. Remote Sens Environ 257:112350. 10.1016/j.rse.2021.112350

[CR327] Tolhurst DJ, Gaynor RC, Gardunia B, Hickey JM, Gorjanc G (2022) Genomic selection using random regressions on known and latent environmental covariates. Theor Appl Genet 135(10):3393–3415. 10.1007/s00122-022-04186-w 36066596 10.1007/s00122-022-04186-w PMC9519718

[CR328] Torres NL, Cho K, Shibato J, Hirano M, Kubo A, Masuo Y, Iwahashi H, Jwa N-S, Agrawal GK, Rakwal R (2007) Gel-based proteomics reveals potential novel protein markers of ozone stress in leaves of cultivated bean and maize species of Panama. Electrophoresis 28(23):4369–4381. 10.1002/elps.200700219 17987633 10.1002/elps.200700219

[CR329] Ustin SL, Gitelson AA, Jacquemoud S, Schaepman M, Asner GP, Gamon JA, Zarco-Tejada P (2009) Retrieval of foliar information about plant pigment systems from high resolution spectroscopy. Remote Sens Environ 113:S67–S77. 10.1016/j.rse.2008.10.019

[CR330] Vallejos CE, Jones JW, Bhakta MS, Gezan SA, Correll MJ (2022) Dynamic QTL-based ecophysiological models to predict phenotype from genotype and environment data. BMC Plant Biol 22(1):275. 10.1186/s12870-022-03624-7 35658831 10.1186/s12870-022-03624-7 PMC9169398

[CR331] van Bezouw RFHM, Keurentjes JJB, Harbinson J, Aarts MGM (2018) Converging phenomics and genomics to study natural variation in plant photosynthetic efficiency. Plant J 97(1):112–133. 10.1111/tpj.14190 10.1111/tpj.14190 PMC6850172 30548574

[CR332] van Bussel LGJ, Ewert F, Zhao G, Hoffmann H, Enders A, Wallach D, Asseng S, Baigorria GA, Basso B, Biernath C, Cammarano D, Chryssanthacopoulos J, Constantin J, Elliott J, Glotter M, Heinlein F, Kersebaum K-C, Klein C, Nendel C, Tao F (2016) Spatial sampling of weather data for regional crop yield simulations. Agric For Meteorol 220:101–115. 10.1016/j.agrformet.2016.01.014

[CR838] van Ittersum MK, Cassman KG, Grassini P, Wolf J, Tittonell P, Hochman Z (2013) Yield gap analysis with local to global relevance—A review. Field Crop Res 143:4–17. 10.1016/j.fcr.2012.09.009

[CR334] van Oosterom EJ, Kulathunga MRDL, Deifel KS, McLean GB, Barrasso C, Wu A, Messina C, Hammer GL (2021) Dissecting and modelling the comparative adaptation to water limitation of sorghum and maize: Role of transpiration efficiency, transpiration rate and height. In Silico Plants 3(1):diaa012. 10.1093/insilicoplants/diaa012

[CR335] van Treuren R, van Eekelen HDLM, Wehrens R, de Vos RCH (2018) Metabolite variation in the lettuce gene pool: Towards healthier crop varieties and food. Metabolomics 14(11):146. 10.1007/s11306-018-1443-8 30830450 10.1007/s11306-018-1443-8 PMC6208706

[CR336] van Zonneveld M, Kindt R, McMullin S, Achigan-Dako EG, N’Danikou S, Hsieh W, Lin Y, Dawson IK (2023) Forgotten food crops in sub-Saharan Africa for healthy diets in a changing climate. Proc Natl Acad Sci 120(14):e2205794120. 10.1073/pnas.2205794120 36972432 10.1073/pnas.2205794120 PMC10083591

[CR337] van der Bom FJT, Williams A, Bell MJ (2020) Root architecture for improved resource capture: Trade-offs in complex environments. J Exp Bot 71(19):5752–5763. 10.1093/jxb/eraa324 32667996 10.1093/jxb/eraa324

[CR338] van Workum DJM, Mehrem SL, Snoek BL, Alderkamp MC, Lapin D, Mulder FFM, den Ackerveken GV, Ridder D de, Schranz ME, Smit S (2024) Lactuca super-pangenome reduces bias towards reference genes in lettuce research (p. 2024.06.20.599299). bioRxiv. 10.1101/2024.06.20.599299 10.1186/s12870-024-05712-2 PMC11514843 39468479

[CR339] VanBuren R, Nguyen A, Marks RA, Mercado C, Pardo A, Pardo J, Schuster J, Aubin BS, Wilson ML, Rhee SY (2024) Variability in drought gene expression datasets highlight the need for community standardization (p. 2024.02.04.578814). bioRxiv. 10.1101/2024.02.04.578814 10.1093/plphys/kiaf653 PMC12854406 41401198

[CR340] Vandemark G, Brick M, Kelly J, Osorno J, Urrea C (2017) YIELD GAINS IN DRY BEANS IN THE U.S. United States Department of Agriculture-Agricultural Research Service / University of Nebraska-Lincoln: Faculty Publications. https://digitalcommons.unl.edu/usdaarsfacpub/1781

[CR341] Vlasova A, Capella-Gutiérrez S, Rendón-Anaya M, Hernández-Oñate M, Minoche AE, Erb I, Câmara F, Prieto-Barja P, Corvelo A, Sanseverino W, Westergaard G, Dohm JC, Pappas GJ, Saburido-Alvarez S, Kedra D, Gonzalez I, Cozzuto L, Gómez-Garrido J, Aguilar-Morón MA, Guigó R (2016) Genome and transcriptome analysis of the Mesoamerican common bean and the role of gene duplications in establishing tissue and temporal specialization of genes. Genome Biol 17(1):32. 10.1186/s13059-016-0883-6 26911872 10.1186/s13059-016-0883-6 PMC4766624

[CR342] Vos J, Evers JB, Buck-Sorlin GH, Andrieu B, Chelle M, de Visser PHB (2010) Functional–structural plant modelling: a new versatile tool in crop science. J Exp Bot 61(8):2101–2115. 10.1093/jxb/erp345 19995824 10.1093/jxb/erp345

[CR343] Voss-Fels KP, Cooper M, Hayes BJ (2018) Accelerating crop genetic gains with genomic selection. Theor Appl Genet 132(3):669–686. 10.1007/s00122-018-3270-8 30569365 10.1007/s00122-018-3270-8

[CR344] Wainberg M, Sinnott-Armstrong N, Mancuso N, Barbeira AN, Knowles DA, Golan D, Ermel R, Ruusalepp A, Quertermous T, Hao K, Björkegren JLM, Im HK, Pasaniuc B, Rivas MA, Kundaje A (2019) Opportunities and challenges for transcriptome-wide association studies. Nat Genet 51(4):592–599. 10.1038/s41588-019-0385-z 30926968 10.1038/s41588-019-0385-z PMC6777347

[CR345] Wallace JG, Rodgers-Melnick E, Buckler ES (2018) On the road to breeding 4.0: unraveling the good, the bad, and the boring of crop quantitative genomics. Ann Rev Genetics 52:421–444. 10.1146/annurev-genet-120116-024846 30285496 10.1146/annurev-genet-120116-024846

[CR346] Wallach D, Hwang C, Correll MJ, Jones JW, Boote K, Hoogenboom G, Gezan S, Bhakta M, Vallejos CE (2018) A dynamic model with QTL covariables for predicting flowering time of common bean (Phaseolus vulgaris) genotypes. Eur J Agron 101:200–209. 10.1016/j.eja.2018.10.003

[CR347] Wang C, Han B (2022) Twenty years of rice genomics research: From sequencing and functional genomics to quantitative genomics. Mol Plant 15(4):593–619. 10.1016/j.molp.2022.03.009 35331914 10.1016/j.molp.2022.03.009

[CR348] Wang E, Brown HE, Rebetzke GJ, Zhao Z, Zheng B, Chapman SC (2019) Improving process-based crop models to better capture genotype×environment×management interactions. J Exp Bot 70(9):2389–2401. 10.1093/jxb/erz092 30921457 10.1093/jxb/erz092

[CR349] Wang D, Li J, Wang Y, Wang E (2022) A comparison on predicting functional impact of genomic variants. NAR Genomics Bioinform 4(1):lqab122. 10.1093/nargab/lqab122 10.1093/nargab/lqab122 PMC8759571 35047814

[CR350] Wang DR, Jamshidi S, Han R, Edwards JD, McClung AM, McCouch SR (2024) Positive effects of public breeding on US rice yields under future climate scenarios. Proc Natl Acad Sci 121(13):e2309969121. 10.1073/pnas.2309969121 38498708 10.1073/pnas.2309969121 PMC10990131

[CR351] Wang X, Yan M, Cui S, Li F, Zhao Q, Wang Q, Jiang B, Huang Y, Sun Y, Kong X (2025) Common bean pan-genome reveals abundant variation patterns and relationships of stress response genes and pathways. BMC Genomics 26(1):495. 10.1186/s12864-025-11662-2 40380089 10.1186/s12864-025-11662-2 PMC12084947

[CR352] Washburn JD, Burch MB, Franco JAV (2020) Predictive breeding for maize: Making use of molecular phenotypes, machine learning, and physiological crop models. Crop Sci 60(2):622–638. 10.1002/csc2.20052

[CR353] Washburn JD, Cimen E, Ramstein G, Reeves T, O’Briant P, McLean G, Cooper M, Hammer G, Buckler ES (2021) Predicting phenotypes from genetic, environment, management, and historical data using CNNs. Theor Appl Genet 134(12):3997–4011. 10.1007/s00122-021-03943-7 34448888 10.1007/s00122-021-03943-7

[CR354] Wasson AP, Richards RA, Chatrath R, Misra SC, Prasad SVS, Rebetzke GJ, Kirkegaard JA, Christopher J, Watt M (2012) Traits and selection strategies to improve root systems and water uptake in water-limited wheat crops. J Exp Bot 63(9):3485–3498. 10.1093/jxb/ers111 22553286 10.1093/jxb/ers111

[CR355] Watkins JL, Li M, McQuinn RP, Chan KX, McFarlane HE, Ermakova M, Furbank RT, Mares D, Dong C, Chalmers KJ, Sharp P, Mather DE, Pogson BJ (2019) A GDSL Esterase/Lipase Catalyzes the Esterification of Lutein in Bread Wheat. The Plant Cell. 10.1105/tpc.19.00272 31575724 10.1105/tpc.19.00272 PMC6925002

[CR356] Wei T, van Treuren R, Liu X, Zhang Z, Chen J, Liu Y, Dong S, Sun P, Yang T, Lan T, Wang X, Xiong Z, Liu Y, Wei J, Lu H, Han S, Chen JC, Ni X, Wang J, Liu H (2021) Whole-genome resequencing of 445 Lactuca accessions reveals the domestication history of cultivated lettuce. Nature Genetics 53(5):752–760. 10.1038/s41588-021-00831-0 33846635 10.1038/s41588-021-00831-0

[CR357] Weiyuan H, Ziqiu L, Xiangqian F, Jinhua Q, Aidong W, Shichao J, Danying W, Song C (2024) Estimating key phenological dates of multiple rice accessions using UAV-based plant height dynamics for breeding. Rice Sci. 10.1016/j.rsci.2024.04.007

[CR358] Werner CR, Zaman-Allah M, Assefa T, Cairns JE, Atlin GN (2025) Accelerating genetic gain through early-stage on-farm sparse testing. Trends Plant Sci 30(1):17–20. 10.1016/j.tplants.2024.10.010 39521690 10.1016/j.tplants.2024.10.010

[CR359] Westhues CC, Mahone GS, da Silva S, Thorwarth P, Schmidt M, Richter J-C, Simianer H, Beissinger TM (2021) Prediction of maize phenotypic traits with genomic and environmental predictors using gradient boosting frameworks. Front Plant Sci. 10.3389/fpls.2021.699589 34880880 10.3389/fpls.2021.699589 PMC8647909

[CR360] Williams JR, Jones CA, Kiniry JR, Spanel DA (1989) The EPIC Crop Growth Model. Trans ASAE 32(2):0497–0511. 10.13031/2013.31032

[CR361] Woebbecke DM, Meyer GE, Von Bargen K, Mortensen DA (1995) Color indices for weed identification under various soil, residue, and lighting conditions. Trans ASAE 38(1):259–269

[CR362] Wong CYS (2023) Plant optics: underlying mechanisms in remotely sensed signals for phenotyping applications. AoB PLANTS 15(4):plaq039. 10.1093/aobpla/plad039 10.1093/aobpla/plad039 PMC10407989 37560760

[CR363] Wong CYS, Bambach NE, Alsina MM, McElrone AJ, Jones T, Buckley TN, Kustas WP, Magney TS (2022) Detecting short-term stress and recovery events in a vineyard using tower-based remote sensing of photochemical reflectance index (PRI). Irrig Sci 40(4–5):683–696. 10.1007/s00271-022-00777-z

[CR364] Wong CY, Gilbert ME, Pierce MA, Parker TA, Palkovic A, Gepts P, Magney TS, Buckley TN (2023) Hyperspectral remote sensing for phenotyping the physiological drought response of common and tepary bean. Plant Phenomics 5:0021. 10.34133/plantphenomics.0021 37040284 10.34133/plantphenomics.0021 PMC10076057

[CR365] Wu A (2023) Modelling plants across scales of biological organisation for guiding crop improvement. Funct Plant Biol 50(6):435–454. 10.1071/FP23010 37105931 10.1071/FP23010

[CR366] Wu A, Hammer GL, Doherty A, von Caemmerer S, Farquhar GD (2019) Quantifying impacts of enhancing photosynthesis on crop yield. Nature Plants 5(4):380–388. 10.1038/s41477-019-0398-8 30962528 10.1038/s41477-019-0398-8

[CR367] Wu A, Brider J, Busch FA, Chen M, Chenu K, Clarke VC, Collins B, Ermakova M, Evans JR, Farquhar GD, Forster B, Furbank RT, Groszmann M, Hernandez-Prieto MA, Long BM, Mclean G, Potgieter A, Price GD, Sharwood RE, Hammer GL (2022) A cross-scale analysis to understand and quantify the effects of photosynthetic enhancement on crop growth and yield across environments. Plant Cell Environ 46(1):23–44. 10.1111/pce.14453 36200623 10.1111/pce.14453 PMC10091820

[CR368] Wu D-H, Chen C-T, Yang M-D, Wu Y-C, Lin C-Y, Lai M-H, Yang C-Y (2022b) Controlling the lodging risk of rice based on a plant height dynamic model. Bot Stud 63(1):25. 10.1186/s40529-022-00356-7 36008613 10.1186/s40529-022-00356-7 PMC9411474

[CR369] Wu A, Truong SH, McCormick R, van Oosterom EJ, Messina CD, Cooper M, Hammer GL (2024) Contrasting leaf-scale photosynthetic low-light response and its temperature dependency are key to differences in crop-scale radiation use efficiency. New Phytol 241(6):2435–2447. 10.1111/nph.19537 38214462 10.1111/nph.19537

[CR370] Xiao J, Moody A (2005) A comparison of methods for estimating fractional green vegetation cover within a desert-to-upland transition zone in central New Mexico, USA. Remote Sens Environ 98(2):237–250. 10.1016/j.rse.2005.07.011

[CR371] Xu K, Xu X, Fukao T, Canlas P, Maghirang-Rodriguez R, Heuer S, Ismail AM, Bailey-Serres J, Ronald PC, Mackill DJ (2006) Sub1A is an ethylene-response-factor-like gene that confers submergence tolerance to rice. Nature 442(7103):705–708. 10.1038/nature04920 16900200 10.1038/nature04920

[CR372] Xu C, Jiao C, Zheng Y, Sun H, Liu W, Cai X, Wang X, Liu S, Xu Y, Mou B, Dai S, Fei Z, Wang Q (2015) De novo and comparative transcriptome analysis of cultivated and wild spinach. Sci Rep 5(1):17706. 10.1038/srep17706 26635144 10.1038/srep17706 PMC4669492

[CR373] Xu C, Jiao C, Sun H, Cai X, Wang X, Ge C, Zheng Y, Liu W, Sun X, Xu Y, Deng J, Zhang Z, Huang S, Dai S, Mou B, Wang Q, Fei Z, Wang Q (2017) Draft genome of spinach and transcriptome diversity of 120 Spinacia accessions. Nat Commun 8(1):15275. 10.1038/ncomms15275 28537264 10.1038/ncomms15275 PMC5458060

[CR374] Xu Y, Ma K, Zhao Y, Wang X, Zhou K, Yu G, Li C, Li P, Yang Z, Xu C, Xu S (2021) Genomic selection: a breakthrough technology in rice breeding. The Crop Journal 9(3):669–677. 10.1016/j.cj.2021.03.008

[CR375] Yahia EM, de Jesús Ornelas-Paz J, Emanuelli T, Jacob-Lopes E, Zepka LQ, Cervantes-Paz B (2017) Chemistry, Stability, and Biological Actions of Carotenoids. In Fruit and Vegetable Phytochemicals (pp. 285–346). John Wiley & Sons, Ltd. 10.1002/9781119158042.ch15

[CR377] Yan J, Yu L, Xuan J, Lu Y, Lu S, Zhu W (2016) De novo transcriptome sequencing and gene expression profiling of spinach (*Spinacia oleracea* L.) leaves under heat stress. Sci Rep 6(1):19473. 10.1038/srep19473 26857466 10.1038/srep19473 PMC4746569

[CR378] Yang X-D, Tan H-W, Zhu W-M (2016) SpinachDB: a well-characterized genomic database for gene family classification and SNP information of spinach. PLoS ONE 11(5):e0152706. 10.1371/journal.pone.0152706 27148975 10.1371/journal.pone.0152706 PMC4858205

[CR379] Yang M-D, Huang K-S, Kuo Y-H, Tsai HP, Lin L-M (2017) Spatial and spectral hybrid image classification for rice lodging assessment through UAV imagery. Remote Sens. 10.3390/rs9060583

[CR380] Yang X, Wei S, Liu B, Guo D, Zheng B, Feng L, Liu Y, Tomás-Barberán FA, Luo L, Huang D (2018) A novel integrated non-targeted metabolomic analysis reveals significant metabolite variations between different lettuce (*Lactuca sativa* L.) varieties. Hortic Res 5:33. 10.1038/s41438-018-0050-1 29977569 10.1038/s41438-018-0050-1 PMC6015802

[CR381] Yang P, van der Tol C, Campbell PKE, Middleton EM (2021a) Unraveling the physical and physiological basis for the solar- induced chlorophyll fluorescence and photosynthesis relationship using continuous leaf and canopy measurements of a corn crop. Biogeosciences 18(2):441–465. 10.5194/bg-18-441-2021

[CR382] Yang Y, Saand MA, Huang L, Abdelaal WB, Zhang J, Wu Y, Li J, Sirohi MH, Wang F (2021b) Applications of multi-omics technologies for crop improvement. Front Plant Sci 12:563953. 10.3389/fpls.2021.563953 34539683 10.3389/fpls.2021.563953 PMC8446515

[CR383] Yang Y, Wilson LT, Li T, Paleari L, Confalonieri R, Zhu Y, Tang L, Qiu X, Tao F, Chen Y, Hoogenboom G, Boote KJ, Gao Y, Onogi A, Nakagawa H, Yoshida H, Yabe S, Dingkuhn M, Lafarge T, Wang J (2022) Integration of genomics with crop modeling for predicting rice days to flowering: a multi-model analysis. Field Crops Res 276:108394. 10.1016/j.fcr.2021.108394

[CR384] Yang X, Lu X, Xie P, Guo Z, Fang H, Fu H, Hu X, Sun Z, Cen H (2024) PanicleNeRF: low-cost, high-precision in-field phenotyping of rice panicles with smartphone. Plant Phenom 6:0279. 10.34133/plantphenomics.0279 10.34133/plantphenomics.0279 PMC11617619 39639877

[CR386] Yobi A, Angelovici R (2018) A high-throughput absolute-level quantification of protein-bound amino acids in seeds. Curr Protocols Plant Biol. 10.1002/cppb.20084 10.1002/cppb.20084 30408333

[CR387] Yoosefzadeh-Najafabadi M, Singh KD, Pourreza A, Sandhu KS, Adak A, Murray SC, Eskandari M, Rajcan I (2023) Chapter four - remote and proximal sensing: how far has it come to help plant breeders? In: Sparks DL (ed) Advances in agronomy. Academic Press, Cambridge, pp 279–315. 10.1016/bs.agron.2023.05.004

[CR388] Yu Y, Cheng Q, Wang F, Zhu Y, Shang X, Jones A, He H, Song Y (2023) Crop/Plant Modeling Supports Plant Breeding: I. Optimization of Environmental Factors in Accelerating Crop Growth and Development for Speed Breeding. Plant Phenomics 5:0099. 10.34133/plantphenomics.0099 37817886 10.34133/plantphenomics.0099 PMC10561689

[CR389] Yun H, Lo S, Diepenbrock CH, Bailey BN, Earles JM (2024) VisTA-SR: Improving the Accuracy and Resolution of Low-Cost Thermal Imaging Cameras for Agriculture

[CR390] Zadražnik T, Hollung K, Egge-Jacobsen W, Meglič V, Šuštar-Vozlič J (2013) Differential proteomic analysis of drought stress response in leaves of common bean (*Phaseolus vulgaris* L.). J Proteomics 78:254–272. 10.1016/j.jprot.2012.09.021 23026550 10.1016/j.jprot.2012.09.021

[CR391] Zampieri M, Weissteiner CJ, Grizzetti B, Toreti A, van den Berg M, Dentener F (2020) Estimating resilience of crop production systems: from theory to practice. Sci Total Environ 735:139378. 10.1016/j.scitotenv.2020.139378 32480148 10.1016/j.scitotenv.2020.139378 PMC7374405

[CR392] Zang J, Jin S, Zhang S, Li Q, Mu Y, Li Z, Li S, Wang X, Su Y, Jiang D (2023) Field-measured canopy height may not be as accurate and heritable as believed: evidence from advanced 3D sensing. Plant Methods 19(1):39. 10.1186/s13007-023-01012-2 37009892 10.1186/s13007-023-01012-2 PMC10069135

[CR393] Zhang W, Alseekh S, Zhu X, Zhang Q, Fernie AR, Kuang H, Wen W (2020) Dissection of the domestication-shaped genetic architecture of lettuce primary metabolism. Plant J 104(3):613–630. 10.1111/tpj.14950 32772408 10.1111/tpj.14950

[CR394] Zhang H, Hou Q, Luo B, Tu K, Zhao C, Sun Q (2022) Detection of seed purity of hybrid wheat using reflectance and transmittance hyperspectral imaging technology. Front Plant Sci 13:1015891. 10.3389/fpls.2022.1015891 36247557 10.3389/fpls.2022.1015891 PMC9554440

[CR395] Zhang Y, Jiang Y, Xu B, Yang G, Feng H, Yang X, Yang H, Liu C, Cheng Z, Feng Z (2024) Study on the Estimation of leaf area index in rice based on UAV RGB and multispectral data. Remote Sens. 10.3390/rs16163049

[CR396] Zhao G, Hoffmann H, van Bussel LGJ, Enders A, Specka X, Sosa C, Yeluripati J, Tao F, Constantin JJ, Raynal HH, Teixeira E, Grosz B, Doro L, Zhao Z, Nendel C, Kiese R, Eckersten H, Haas E, Vanuytrecht E, Ewert F (2015) Effect of weather data aggregation on regional crop simulation for different crops, production conditions, and response variables. Clim Res 65:141. 10.3354/cr01301

[CR397] Zhao X, Yuan Y, Song M, Ding Y, Lin F, Liang D, Zhang D (2019) Use of unmanned aerial vehicle imagery and deep learning unet to extract rice lodging. Sensors. 10.3390/s19183859 31500150 10.3390/s19183859 PMC6766838

[CR398] Zhao J, Kumar A, Banoth BN, Marathi B, Rajalakshmi P, Rewald B, Ninomiya S, Guo W (2022) Deep-learning-based multispectral image reconstruction from single natural color RGB image—enhancing UAV-based phenotyping. Remote Sens. 10.3390/rs14051272

[CR399] Zheng Q, Huang W, Xia Q, Dong Y, Ye H, Jiang H, Chen S, Huang S (2023) Remote sensing monitoring of rice diseases and pests from different data sources: a review. Agronomy. 10.3390/agronomy13071851

[CR400] Zhong Y, Xu T, Chen Q, Li K, Zhang Z, Song H, Wang M, Wu X, Lu B (2020) Development and validation of eight cyanogenic glucosides via ultra-high-performance liquid chromatography-tandem mass spectrometry in agri-food. Food Chem 331:127305. 10.1016/j.foodchem.2020.127305 32593038 10.1016/j.foodchem.2020.127305

[CR401] Zhou J, Zhou J, Ye H, Ali ML, Nguyen HT, Chen P (2020) Classification of soybean leaf wilting due to drought stress using UAV-based imagery. Comput Electron Agric 175:105576. 10.1016/j.compag.2020.105576

[CR402] Zhou Y, Kusmec A, Mirnezami SV, Attigala L, Srinivasan S, Jubery TZ, Schnable JC, Salas-Fernandez MG, Ganapathysubramanian B, Schnable PS (2021) Identification and utilization of genetic determinants of trait measurement errors in image-based, high-throughput phenotyping. Plant Cell 33(8):2562–2582. 10.1093/plcell/koab134 34015121 10.1093/plcell/koab134 PMC8408462

[CR403] Zhu X, Leiser WL, Hahn V, Würschum T (2021) Phenomic selection is competitive with genomic selection for breeding of complex traits. Plant Phenome Journal 4(1):e20027. 10.1002/ppj2.20027

